# Gene Expression Pattern of Peyer’s Patch Lymphocytes Exposed to Kagocel Suggests Pattern-Recognition Receptors Mediate Its Action

**DOI:** 10.3389/fphar.2021.679511

**Published:** 2021-08-03

**Authors:** Alexander A. Andreev-Andrievskiy, Roman A. Zinovkin, Mikhail A. Mashkin, Olga Yu. Frolova, Yuriy G. Kazaishvili, Victoria S. Scherbakova, Boris A. Rudoy, Vladimir G. Nesterenko

**Affiliations:** ^1^ Biology Faculty, M.V. Lomonosov Moscow State University, Moscow, Russia; ^2^ MSU Institute for Mitoengineering, Moscow, Russia; ^3^ Institute of Biomedical Problems RAS, Moscow, Russia; ^4^ A.N. Belozersky Institute of Physico-Chemical Biology, M.V. Lomonosov Moscow State University, Moscow, Russia; ^5^ N.F. Gamaleya Federal Research Centre of Epidemiology and Microbiology, Moscow, Russia

**Keywords:** kagocel, gene expression, cytokines, pattern recognition receptors, Peyer’s patch lymphocytes

## Abstract

Kagocel is a synthetic carboxymethylcellulose derivative copolymerized with gossypol. Clinical data evidence its safety and efficiency for the treatment of flu and other viral infections via enhancement of interferon production. The gut-associated lymphoid tissue seems a likely site of kagocel action. The study was aimed to investigate the molecular mechanisms of its action using murine Peyer’s patches lymphocytes as a test system and the cytokines production and gene expression patterns as the primary outcomes. The Peyer’s patches lymphocytes isolated from BALB/c mice were stimulated with concanavalin A, or, to mimic viral infection, with a combination of concanavalin A and TLR3 ligand poly I:C. After 24 h of stimulation the cells were treated with saline, 30, 100, or 300 μg/ml of kagocel, or, as positive controls, 300 μg/ml oats b-D-glucan or 300 μg/ml lentinan. After 24 and 72 h of incubation with these drugs cytokines production was analyzed with ELISA and gene expression pattern was investigated using nCounter Inflammation panel chips followed by bioinformatics analysis. Expression of genes involved in the inflammatory response, antiviral defense, lymphocytes survival and proliferation (C1qa, C2, C3, Ccl21a, Il11, Il1b, Il23a, Il5, Ltb4r2, Alox15, Pla2g4a, Ptger1, Mapkapk5, Hras, Ifna1, Tlr2, Mrc1, Mx2) was upregulated in kagocel-treated Peyer’s patches lymphocytes. A list of plausible transcription factors (CEBPs, IRF, NFκB, RXR, Stat, Tead4, and ZSCAN) and master-regulators has been identified (cIAP, CIKS, dock9, MEKK1, FXR, IKK, IRAK, TRAF, dsRNA:TLR3:TRIF). The changes in gene expression pattern and the outcomes of bioinformatics analysis suggest that pattern recognition receptors, TLRs and dectin-1, are the key mediators of kagocel immunomodulatory action, with the possible involvement of interferon autocrine loop. The genes upregulated with kagocel include diverse components of the innate immune defense system.

## Introduction

The breakdown products of bacteria, fungi, and viruses trigger a rapid reaction of immune cells and some other cell types in the body as a part of an innate immune response (Kieser and Kagan, 2017). Among these breakdown products, (pathogen-associated molecular patterns, PAMPs) polysaccharides of the bacterial and fungal cell wall upon binding to toll-like receptors (Iwasaki and Medzhitov, 2004), dectin-1 receptors, and possibly other receptor types (Brown, 2005) induce inflammation, interferon production, promote immune cells survival and proliferation (Iwasaki and Medzhitov, 2004; Brown, 2005; Kieser and Kagan, 2017). Several polysaccharides of natural origin have been attributed immunomodulatory activity, among them lentinan (Borchers et al., 1999) and plant β-D-glucans (Estrada et al., 1997; Tada et al., 2009) have drawn the most attention as possible therapeutical agents, while others, like zymosan (CARLO and FIORE, 1958) or carrageenan (Necas and Bartosikova, 2013) have proven to be useful research tools as immune response/inflammation inductors. Of note, the immunomodulatory effects of glucans have been reported in diverse species of mammals, birds, fish, and even invertebrates, indicating the involvement of an evolutionary conserved pathway/mechanism (Vos et al., 2007).

The naturally occurring glucans stimulate macrophage activity, adaptive B- and T-cell mediated immune responses, and possess anti-cancer activity (Brown and Gordon, 2003). Some of the glucans are traditionally used as remedies for multiple diseases (Bisen et al., 2010). Kagocel is a synthetic copolymer of modified carboxymethylcellulose and a natural polyphenol, gossypol, which was designed at the Gamaleya Research Institute of Epidemiology and Microbiology and further marketed as an oral interferon inductor in Russia and CIS countries by Nearmedic, LLC. Gossypol, no more than 3% by weight, is covalently bound to the carboxymethylcellulose backbone. The rationale for the inclusion of gossypol into the polymer was its immunomodulatory properties (Ershov et al., 1988), while the covalent binding immobilized the polyphenol and thus reduced its toxicity (Eagle and Castillon, 1948) or possible male fertility effects (Lim et al., 2019). Indeed, gossypol is not released from kagocel upon storage or incubation with gastric or intestinal juice (Sinitsin et al., 2020). No adverse effects were identified in the chronic and reproduction toxicity studies with moderate doses of kagocel (Borovskaya, 2017).

Clinical data corroborate the efficacy of kagocel for influenza treatment (Fazylov et al., 2016; Sologub and Tsvetkov, 2017) and for fighting other viral infections (Galegov et al., 2002; Loginova et al., 2020) as a monotherapy or a part of a combination treatment (Popov et al., 2017b). Analysis of interferon concentrations in plasma of patients with influenza evidence increased cytokine levels upon kagocel administration (Popov et al., 2017a). However, the molecular mechanisms of kagocel action are poorly understood. Aiming to provide cues to the possible mechanisms of kagocel action, we used murine Peyer’s patches lymphocytes as a test system, deliberately avoiding further isolation of specific cell types to preserve cellular interactions. To preferentially stimulate T-lymphocytes, we used concanavalin A as a mitogen, or, to mimic viral infection, a combination of concanavalin A and TLR3 ligand poly I:C. The stimulated lymphocytes were incubated with different concentrations of kagocel or, as a positive control, with well-defined glucans, lentinan, and oats β-D-glucan, and analyzed for cytokine production and gene expression patterns at 24 and 72 h after treatment.

## Results

### Cytokines Production

**FIGURE 1:**
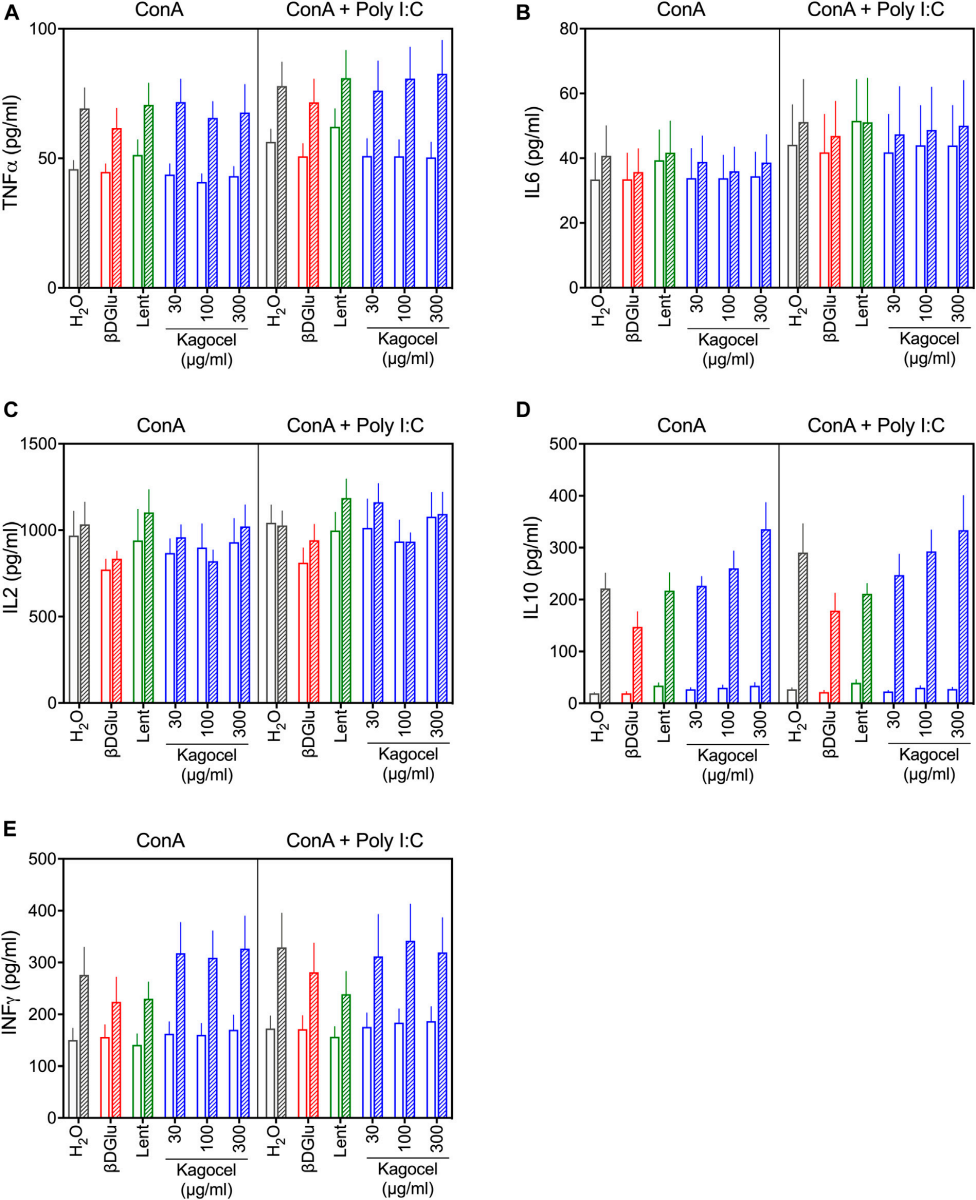
TNFα **(A)**, IL6 **(B)**, IL2 **(C)**, IL10 **(D)** and INFγ **(E)** concentration in the media after 24 (open bars) and 72 h (hatched bars) of cultivation of Peyer’s patch lymphocytes stimulated with concanavalin A (ConA, open bars) in absence or presence of TLR-3 ligand poly I:C. Cells were treated with saline (H_2_O), 300 μg/ml oats β-D Glucan (βDGlu), 300 μg/ml lentinan (Lent) or 30-300 μg/ml kagocel. Results present mean ± s.e.m from 6 independent runs of the experiment. For statistics on drug effects see text.

The cytokines content in the culture media was analyzed after 24 and 72 h cultivation with different polysaccharides (Figure 1). Analysis of variance, expectedly, revealed significant effects of time and mitogen stimulation applied on the concentration of all the cytokines studied, except for IL10, which was not affected by the type of mitogen (Figure 1). Among the polysaccharides, β-D Glucan had significant effects on TNFα (F_(1.000,__10.00)_ = 14.1, *p* = 0.004), IL2 (F_(1.000,__10.00)_ = 5.26, *p* = 0.045), Il10 (F_(1.000,__10.00)_ = 5.78, *p* = 0.037), and INFγ levels (F_(1.000,__10.00)_ = 5.46, *p* = 0.042). Lentinan increased the levels of TNFα (F_(1.000,__10.00)_ = 5.81, *p* = 0.037) and IL6 (F_(1.000,__10.00)_ = 8.68, *p* = 0.015), while INFγ was decreased with this treatment (F_(1.000, 10.00)_ = 15.48, *p* = 0.003). Kagocel had no consistent effect on TNFα, IL2, IL6, and INFγ, however, incubation of lymphocytes with kagocel elevated IL10 concentration (F_(3.000,__30.00)_ = 6.27, *p* = 0.002) in a seemingly dose-dependent manner (Figure 1D). The enhanced IL10 production by kagocel-treated cells might explain the lack of the drug’s effects on pro-inflammatory cytokines production by stimulated lymphocytes.

### Gene Expression

After obtaining the gene expression data expressed as normalized counts, first, we performed the cluster analysis. The derived clustering matched the design of the experiment, with the uppermost two factors in the output hierarchy being time and the mitogen applied (Supplementary Table S1). Thus, we conclude our experimental design had effectively altered the expression patterns for inflammation-related genes in the murine Peyer’s patches lymphocytes.

**FIGURE 2:**
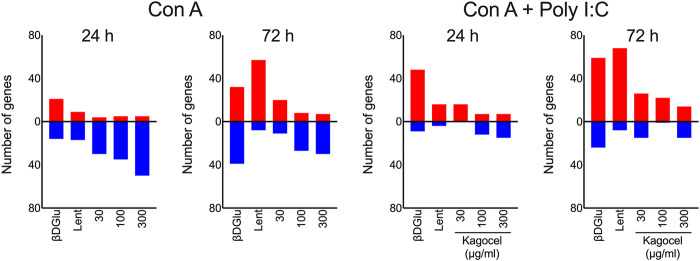
The counts of up- (red) and downregulated (blue) inflammation-related genes in Peyer’s patches lymphocytes stimulated with concanavalin A (ConA) in absence or presence of TLR-3 ligand poly I:C 24 and 72 h after treatment with 300 μg/ml oats β-D Glucan (βDGlu), 300 μg/ml lentinan (Lent) or 30-300 μg/ml kagocel as compared to control, saline-treated, lymphocytes. RNA was collected in 3 independent runs of the experiment.

Next, for a bird’s-eye view of the data, we’ve counted the up- and down-regulated genes, as compared to the matching control cells, for each combination of the drug applied, mitogen stimulation, and time. As shown in Figure 2, upon treatment with polysaccharides, downregulation prevailed in concanavalin A treated lymphocytes at 24 h of incubation, except for lentinan, which upregulated the gene expression. At 72 h of incubation, the number of upregulated genes exceeded the downregulated genes count. Unlike that, when concanavalin A stimulation was complemented with poly I:C as a TLR3 ligand, the number of upregulated genes was generally higher than the number of down-regulated inflammation-related genes. The number of down-regulated genes increased with the concentration of kagocel in the incubation media in concanavalin A stimulated cells. Of note, in presence of poly I:C, no downregulated genes have been identified in kagocel-treated lymphocytes poly I:C at some concentrations of the drug.

**FIGURE 3:**
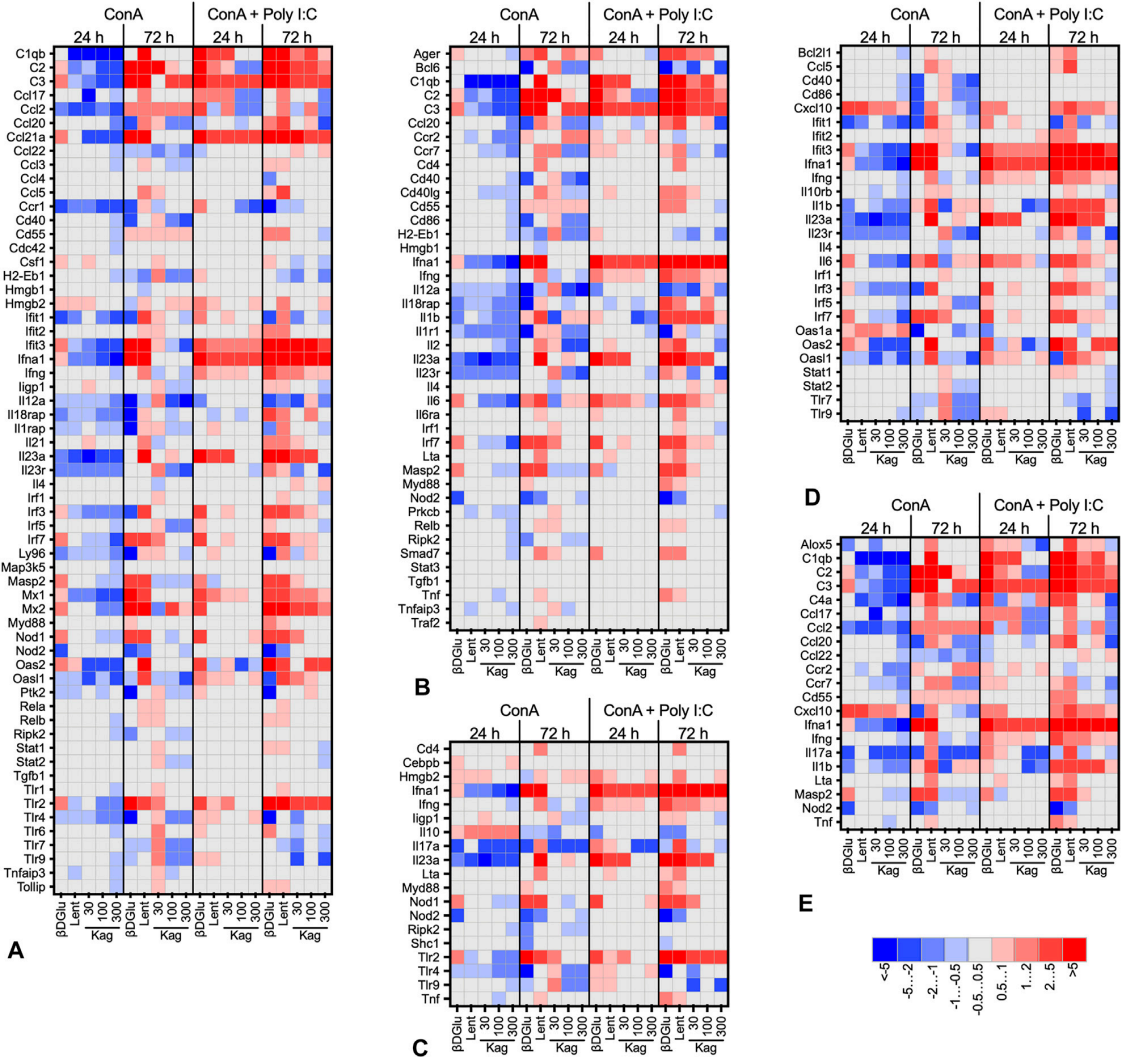
Heat map (log_2_-fold change of expression) of genes involved in innate immune response, GO:0045087 **(A)**, adaptive immune response, GO:0002250 **(B)**, defense response to bacteria, GO:0042742 **(C)**, defense response to virus, GO:0051607 **(D)**, and humoral immune response, GO:0019730 **(E)** in murine Peyer’s patches lymphocytes stimulated with concanavalin A (ConA) in absence or presence of TLR-3 ligand poly I:C after 24 and 72 h after treatment with 300 μg/ml oats β-D Glucan, 300 μg/ml lentinan or 30-300 μg/ml kagocel (Kag) as compared to control, saline-treated, lymphocytes. RNA was collected in 3 independent runs of the experiment.

The heat maps presented in Figure 3 give a more detailed view of the data (see Supplementary Table S2 for the complete list). For this presentation, we have grouped the responding genes by Gene Ontology biological process. Overall, the expression patterns had a similar profile for all the processes analyzed. Downregulation was prevalent in concanavalin A-stimulated cells, while upon a more robust stimulation with a combination of concanavalin A and poly I:C upregulation was more frequent. More pronounced changes of gene expression were observed at 72 h of cultivation with the polysaccharides, while at 24 h the gene expression responses were more temperate. Finally, while expression patterns upon cell treatment with β-D Glucan and lentinan were alike, the effects of kagocel were dissimilar to these two glucans. Kagocel was applied to the cells at three different concentrations; of note, the most pronounced effects on gene expression were observed, counterintuitively, with the lowest concentration of the drug.

The responder genes in kagocel-treated lymphocytes were involved in the regulation of macromolecule synthesis (GO:0031326, GO:0010556, GO:2000112), regulation of transcription (GO:0006355, GO:2001141, GO:1903506), and apoptosis (GO:0006915, GO:0012501, GO:0008219). Specifically, the genes upregulated in Peyer’s patch lymphocytes were those encoding for the components of the complement system (C1qa, C2, C3), cytokine, chemokine, and lipid signaling (Ccl21a, Il11, Il1b, Il23a, Il5, Ltb4r2, Alox15, Pla2g4a, Ptger1), intracellular signal transduction (Mapkapk5, Hras) and, importantly, innate sensing and response to pathogens (Defa-rs1, Ifna1, Tlr2, Mrc1, Mx2).

### Transcription Factors Associated With Gene Response

Aiming to elucidate the possible mechanism of kagocel action, we used the responder genes as an input for the enrichment analysis of the transcription factor binding sites. By cross-tabulating the results of this analysis for different experimental conditions we have identified several transcription factors that could potentially govern the kagocel-induced changes of expression in the Peyer’s patch lymphocytes. Among these factors, the CEBPs, homeobox, interferon regulatory factors, NFκB, retinoid X receptor alpha, Stat, Tead4, and Zinc finger and SCAN domain-containing proteins had the greatest fold enrichment. Of note, virtually identical transcription factors were highlighted for the two control glucans, oats β-D glucan and lentinan. The complete list of identified transcriptional factors is presented in Supplementary Table S3.

### Master Regulators

We extended our search for the mechanisms of kagocel action further and performed the analysis of potential master regulators and pathways. Typically for this type of analysis, the output brought a wide range of possible regulators, rather than a single candidate. To exclude the unmeaningful results, we cross-tabulated the data for the same drug at different experimental conditions (mitogen and time point), while in the case of kagocel we could also include the data for the three doses into this analysis. The regulators and pathways with single or unsystematic entries were disregarded. Thus, we were able to identify cIAP, CIKS, dock9, MEKK1, FXR, IKK, IRAK, TRAF, dsRNA:TLR3:TRIF pathway, and several pathways involving TLR signaling as the potential regulators underlying kagocel action on the lymphocyte’s gene expression. The complete list of identified master regulators is presented in Supplementary Table S4.

## Discussion

Here we report the results of gene expression analysis in murine Peyer’s patch lymphocytes upon treatment with a carboxymethylcellulose and gossypol copolymer, kagocel, or naturally occurring glucans, lentinan, and oats β-D glucan, as positive controls. The polysaccharides induced pronounced changes of inflammation and immune genes expression that highlight pattern-recognition receptors as the plausible targets of kagocel action.

We used the primary culture of murine Peyer’s patch lymphocytes as a test system to investigate the effects of kagocel. There are several reasons for this choice. Firstly, it is well documented that the glucans are effective upon oral administration, and the same is true for the synthetic glucan, kagocel, which is taken orally as an immunostimulant. However, due to the high molecular weight of these polymers ( >10^5^), they are poorly absorbed from the intestine (Vos et al., 2007). The pharmacokinetic data for polysaccharides of a similar molecular mass evidence that their bioavailability upon oral exposure is in the range of hundredths to tenths of percent (Mehvar and Shepard, 1992). In line with that, only about 10% of the kagocel dose is absorbed from the gastrointestinal tract of rats upon oral administration, presumably, the lower-molecular weight fraction of the polymer (Andreev-Andrievskiy et al., 2019). The discrepancy between the apparent effects of the glucans upon the immune system and their poor absorbance can be resolved by observations of specific accumulation of the polysaccharides in the Peyer’s patches after oral exposure. Thus, after ingestion of carrageenan, it has been identified in the Peyer’s patches (Nicklin et al., 1988). Glucans can be internalized by M-cells upon binding to dectin-1 receptor (Smet et al., 2013), and intestinal epitheliocytes might contribute to the uptake of particulate antigens above the Peyer’s patches (Howe et al., 2014). Although kagocel distribution in different tissues of the intestinal wall has not been studied, we speculate that kagocel may penetrate the Peyer’s patches upon oral exposure, which would make them a potential site of its action upon immunity.

The Peyer’s patches lymphocytes are a mixed population, consisting of 60% B-cells, 25% of T-cells, 10% of dendritic cells (CD11c+), and less than 5% of macrophages or polymorphonuclear neutrophils (Lefrançois and Lycke, 1996; Jung et al., 2010). While this is true for the freshly isolated cells, we utilized the stimulation protocol specifically favoring T-cell proliferation (Dwyer and Johnson, 1981). Thus, considering the relatively long period of cultivation with the mitogens before application of the drugs, diverse subsets of T-cells, presumably, comprised the majority of the culture studied.

Substantial evidence accumulated over the recent years suggests that the link between the respiratory tract and the intestinal immune systems is critically important for host antiviral defense. On the one hand, respiratory viruses may induce intestinal injury as seen for influenza (Wang et al., 2014), or, more recently for SARS-CoV-2 (Scaldaferri et al., 2020). The infected epithelial cells can present viral antigens to promote cell-mediated immunity (Nguyen et al., 1998). At the same time, the intestinal immune system, primarily the Peyer’s patch, is an important site of the lymphocytes emigration (Rothkötter et al., 1999; Heidegger et al., 2013). Of note, dendritic cells constitute up to 4% of the immigrating cells (Rothkötter et al., 1999). Basing on the existing links between the microbiota, the intestinal immune system, and overall hosts’ resistivity to infection (Ichinohe et al., 2011; Clemente et al., 2012), several strategies have been suggested employing the modification of a diet or direct introduction of some strains of commensal bacteria (Villena et al., 2012; Goto et al., 2013; Illiano et al., 2020). Interestingly, the outcomes of anti-influenza vaccination rely on commensal microbiota sensing via TLRs (Oh et al., 2014), thus the oral influenza vaccines that are being developed directly utilize TLR ligands as adjuvants (Lycke, 2012). Moreover, some polysaccharides have been suggested as adjuvants for oral vaccines (Smet et al., 2013).

The genes upregulated in the intestinal lymphocytes after incubation with kagocel include several important players of the innate immune response, components of the complement system, antiviral defense systems and chemokines. While the significance of the plasma complement system for immunity is apparent, the importance of the recently reported expression of the complement system components in T-cells is not completely clear, but might be related to cell survival and differentiation (Hansen et al., 2018). The interaction of signaling from TLRs and the receptors to complement anaphylatoxins significantly alters the immune response (Song, 2012). Less is known about the relevance of kininogen expression in the lymphocytes. Hras1, a. k.a. p21, is a negative regulator of the cell cycle (Khanna et al., 2005), capable of inducing cell-cycle arrest following TLR4/TRIF activation as a part of the antiviral response (Mlcochova et al., 2020). Importantly, H-Ras is capable of enhancing interferon signaling (Chen et al., 2017). Heat shock protein 27 kDa, has a variety of functions, of note, after release to extracellular space HSP27 is capable of activating NF-κB in a TLR4 dependent manner (Shi et al., 2019).

Several genes, important for the lymphocytes migration or chemotaxis, were among the responder genes in kagocel-treated lymphocytes. Thus, CCL21, a ligand of CCR7, was upregulated in PP lymphocytes co-stimulated with concanavalin A and Poly I:C, but not upon stimulation with concanavalin A only. Of relevance, disruption of CCL21/CCR7 signaling weakens the immune response to a variety of viruses, by interfering with the cells’ migration and proper interactions during antigen presentation (Comerford et al., 2013). Leukotriene B4 receptor 2 is another up-regulated mediator of cellular migration. Interestingly, the chemotactic effects of TLR ligands are mediated by this receptor (Lefebvre et al., 2010). Upon treatment with kagocel, the PP lymphocytes expressed more RNA for TLR2, a sensor of bacterial lipoproteins, and lipoteichoic acids. One of the diverse ligands of this receptor is zymosan, a fungal β-glucan (Sato et al., 2003). However, TLR2 is known to bind a wide variety of ligands, and to form functional heterodimers with other types of TLR and non-TLR molecules (Oliveira-Nascimento et al., 2012). One of the TLR2 dimerization partners is TLR6, which was also upregulated in kagocel treated cells, along with 7 and 9, however to a lesser extent as compared to TLR2.

IL23 is a pro-inflammatory cytokine with a multi-faceted effect on T-cell differentiation and INFγ production (Guo et al., 2019). As reviewed in (Novelli and Casanova, 2004), IL23 deficiency results in a substantial decrease of resistivity to diverse DNA and RNA viruses, thus induction of IL23 expression is one of the apparent positive effects of kagocel for antiviral defense in the lymphocytes. IFIT3 is an interferon-induced protein, also up-regulated with virus infection and double-stranded RNA, capable of inhibiting viral replication and translational initiation, along with cellular migration and proliferation (Fensterl and Sen, 2011). Overexpression of IFIT3 alone was sufficient to suppress viral replication in a porcine model (Li et al., 2015). Another gene up-regulated in kagocel treated PP lymphocytes is defensin-related sequence 1, a cysteine-rich protein specific to the rodent intestine and not found in the human (Andersson et al., 2012).

Myxovirus resistance proteins 1 and 2 were both up-regulated with kagocel treatment, providing another mechanistic explanation for the efficacy of the drug. These GTPases inhibit different viruses by blocking the early steps of viral replication (Haller et al., 2015). Recombinant Mx-1, modified to facilitate cell penetration, was capable of effectively suppressing influenza infection *in vitro* and *in vivo* (Jung et al., 2019). Interestingly, the MX protein can be induced by both type I and III interferons (Haller et al., 2015). Due to the limitations of the gene expression analysis system, we have no experimental data on type III interferon expression. Considering that both types I and III interferons are concurrently induced by TLR (Ank et al., 2008) and dectin-1 activation (Dutta et al., 2020), it is tempting to speculate that type III interferon was upregulated in kagocel-treated cells in our study and mediated some of the effects of the drug. Yet another component of the cellular anti-viral defense systems responsive to kagocel application was 2–5′ Oligoadenylate synthetase 2, an enzyme capable of interrupting viral replication via activation of rnase L and in an oligoadenylate-independent manner (Kristiansen et al., 2010).

Ifna1 was one of the genes with the highest fold-change ( >5) in kagocel-treated cells as compared to control PP lymphocytes. A similar increase in interferon production induced by kagocel has been reported previously using human cell lines (Vershinina et al., 2002). The antiviral properties of interferon are subject to years of scrutiny and a PubMed search “interferon alpha AND virus” yields more than 23 thousand references. Yet more information is available on the use of interferons for the treatment of diverse viral infectious diseases. Importantly, the potentiating effect of kagocel on Ifna expression was evident in presence of TLR-3 ligand poly I:C, evidencing the involvement of additional mechanisms of interferon induction. Both dectin-1 (Dragicevic et al., 2012) and TLR2 receptors (Vanhoutte et al., 2008) have been reported to interact with TLR-3 signaling to significantly modify lymphocyte functions.

Noteworthy, the majority of kagocel-responder genes involved in anti-viral defense were up-regulated only in concanavalin A and poly I:C co-stimulated lymphocytes, while in absence of dsRNA these genes were down-regulated in kagocel-treated cells as compared to control lymphocytes. Among them, Oasl, Il17, Ifit1, and Ccr1 should be listed. On the contrary, Il5 was up-regulated by kagocel in concanavalin A stimulated PP lymphocytes, but no effect upon this cytokine expression was found in Con A and poly I:C co-stimulated cells. Another intriguing possibility is the involvement of type I interferon autocrine loop in the effects of kagocel. Upon activation of TLR receptors, INFα can stimulate the cell in an autocrine manner via INFAR1 (Song et al., 2015), and, importantly, this mechanism has been reported to be involved in the effects of β-D-glucans (Hassanzadeh-Kiabi et al., 2017). Furthermore, type I interferon is capable to increase IL10 production by diverse types of immune cells stimulated with TLR ligands (Gabryšová et al., 2014). Thus, increased IL10 production upon treatment of Peyer’s patch lymphocytes with kagocel (Figure 1D) supports involvement of INFα in the realization of kagocel effects.

Prior to commencing the study, we hypothesized that either Toll-like receptors or the dectin-1 receptor could mediate the immunomodulatory effects of kagocel. In this study, we have performed the search of master molecules mediating the changes in gene expression patterns. Among the candidates for mediating the kagocel action, the key components of TLR-signaling pathways TRAF, Tab, TAK, IKKα and IKKβ, RIP, and IRFs have been identified with high scores and low FDRs, and IRF3 and IFF7 expression was moderately up-regulated with kagocel (Figure 3). Identification of IFNα, IFNβ/IFNAR/Tyk2/Jak pathway, and its separate components among the candidates, supports the hypothesis of interferon autocrine loop involvement in kagocel action upon the PP lymphocytes. Next, our findings support the possibility of dectin-1 receptor involvement in the realization of kagocel effects. Despite dectin-1 signaling is less fully elucidated as compared to other PRRs (Tang et al., 2018), it is apparent that dectin and TLR2 signaling closely interact to mediate glucan-activated anti-fungal immunity (Brown, 2005). In support of the possibility of dectin-1 activation by kagocel, SYK and ZAP-70 have been identified among the master-molecule candidates. Moreover, the signaling from both possible kagocel receptors is convergent in orchestrating the inflammatory response (Gantner et al., 2003). Finally, co-stimulation of TLR and Dectin-1 receptors enhances IL-10 production (Gabryšová et al., 2014), providing one of the possible explanations for enhanced IL10 production observed in kagocel-treated lymphocytes in this study (Figure 1D).

**FIGURE 4:**
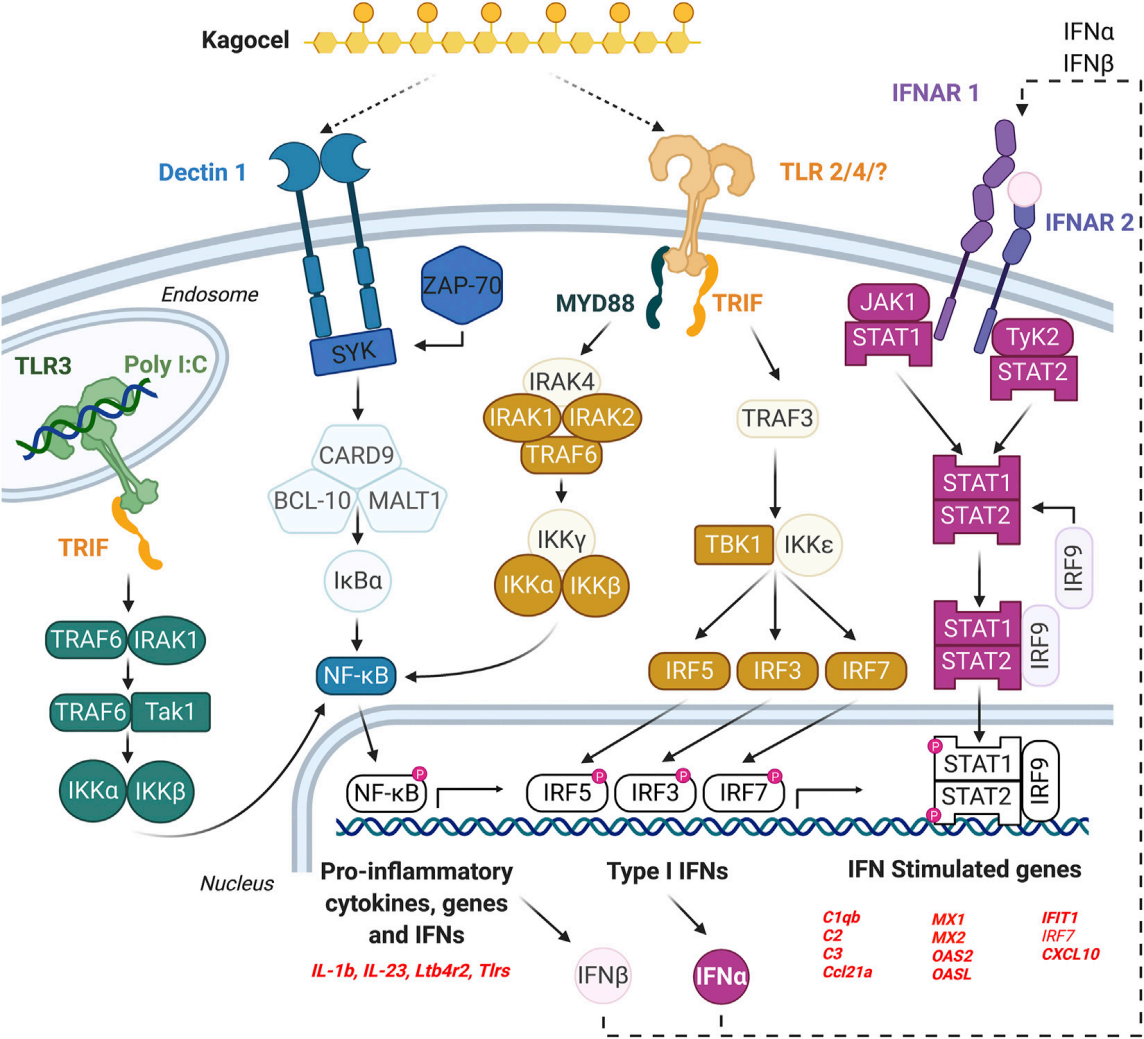
The possible mechanism of kagocel action, as summarized from the study findings. The elements of the network highlighted with bold colors are either genes up-regulated in Peyer’s patch lymphocytes, or the transcription factors/master regulators identified using bioinformatics tools.

The findings of our study are summarized in Figure 4. We conclude that the Peyer’s patches lymphocytes, as important players in the antiviral defense, might be accessible for kagocel upon oral administration, which makes gut-associated lymphoid tissue the likely site of kagocel action. When the mixed population of PP lymphocytes is exposed to kagocel, the drug presumably binds to TLR2 and/or dectin-1 receptors to induce downstream signaling and activation of the innate antiviral response with possible involvement of interferon autocrine loop. These genes up-regulated by kagocel treatment are involved in different stages of antiviral and antibacterial immunity, ranging from sensing the pathogen and the immune response modulation to the effector proteins. Further research is needed to verify the involvement of these mechanisms in the immunomodulatory action of kagocel.

### Materials and Methods

#### Isolation of Peyer’s Patches Lymphocytes

Peyer’s patches lymphocytes were isolated from male BALB/c mice as described previously (Lefrançois and Lycke, 1996). Briefly, mice were humanely killed, the small intestines were excised and placed on ice. The intestinal lumen was flushed with 20 ml ice-cold PBS and the Peyer’s patches were microdissected under the microscope and rinsed thrice with ice-cold PBS supplemented with gentamicin (100 μg/ml). Patches from 6-8 mice were pooled.

To facilitate tissue dissociation, the patches were incubated in CMF (85% HBSS without Mg^2+^, Ca^2+^, and NaHCO_3_; 1xHEPES-bicarbonate buffer and 5% FBS) on a rotation shaker for 30 min at 37°С. After that the tissue was forced through a 70 μm cell strainer, the suspension was pelleted (5 min at 500 rcf) and washed thrice with CMF. Lymphocytes were separated by centrifugation in 44% Percoll layered over 80% Percoll for 20 min at 600 rcf, washed four times in HBSS, and resuspended to 1×10^6^/ ml in RPMI 1640 supplemented with l-glutamine, 10% FBS, and 100 μg/ml gentamicin. To verify the number of viable lymphocytes, cells were differentially stained with propidium iodide (10 µM) and SYBR Green (1 µM), and the number of living cells counted from the microphotographs. In six separate experiments, on average 90 ± 3% of the cells were viable.

#### Lymphocyte Culture

The isolated lymphocytes were split into two aliquots and stimulated with 10 μg/ml concanavalin A or a combination of 10 μg/ml concanavalin A and 10 μg/ml of TLR-3 agonist poly I:C. To ensure proper annealing, poly I:C was heated for 5 min at 55°С and cooled to room temperature for 1 h. The stimulated lymphocytes were seeded into 24-well plates (SPL life sciences, South Korea), 1.5 × 10^6^ cells per well, and incubated at 37°С in 5% CO_2_ atmosphere (MCO-5AC incubator, Sanyo, Japan).

After 24 h of cultivation with the mitogens, water (H_2_O) or 30, 100, or 300 μg/ml kagocel (Kag) were added to the cells in a volume of 15 µL (1:100 v:v). Positive control lymphocytes were treated with 300 μg/ml lentinan (Lent) or 300 μg/ml oats β-D-glucan (βDGlu).

Samples were collected after 24 and 72 h of incubation with the drugs, one well per treatment, at each of the two time points. The cells were pelleted by centrifugation at 500 rcf for 5 min. The culture media was collected and frozen at 80°С for subsequent analysis of cytokines content, while the cells were immediately lysed with 500 µL TRI Reagent for 20 min on a rotation shaker at room temperature.

Thus, expression patterns and the cytokines production for each of the drugs were studied at two time points for each of the two mitogen stimulation protocols. A total of six independent replications of this design were performed. Cytokine concentration was measured in all the runs, while the gene expression pattern was investigated in the three last runs only.

#### ELISA

The culture media were analyzed for INFα, INFγ, IL2, IL10, and TNFα content using RnD kits according to the manufacturer’s instructions.

The statistical analysis of the cytokine concentrations was performed separately for each of the tested drugs using three-way ANOVA (factors “Time,” “Mitogen” and “Drug”) with Prism (v. 8.0, GraphPad, United States). Only the main effects and interactions were analyzed and considered significant at *p* < 0.05.

#### RNA Isolation

To achieve phase separation, the TRI Reagent lysates were treated with 1 bromo-3-chloropropane (1:10 v:v), thoroughly mixed, and centrifuged at 12,000 rcf and 4°C for 15 min. The aqueous phase was collected and further purified using RNeasy micro kits (Qiagen, United States) according to the manufacturer’s instructions. The average yield was ≈800 ng per sample, as measured with Qubit 4 Fluorometer (Invitrogen, United States). RNA preparation purity was analyzed with NanoDrop (Thermo Scientific, United States) and preparations with suboptimal quality (A_260_/A_280_ < 1.8, A_260_/A_230_ < 1.8) were excluded from further analysis. The replicate samples from three independent runs were mixed at equal quantities and stored at −80°С until subsequent analysis.

#### Gene Expression Analysis

Analysis of gene expression was performed using murine nCounter Inflammation panel chips and analyzed using nCounter software (NanoStiring Technologies, United States) according to the manufacturer’s instructions (Kulkarni, 2011). Briefly, the raw counts were thresholded using the mean plus 2 standard deviations of the in-built negative controls counts, individually for each chip. Next, the counts were normalized to the coefficient derived from the in-built positive controls counts. Finally, the second normalization to the geometric mean of the housekeeping genes expression for individual samples was applied.

#### Prediction of Transcription Factors

As a next step, separately for each of the substances tested, fold-change was calculated for each of the target genes against expression level in the control (saline-treated) sample at the corresponding time point and expressed as log_2_. Genes with a twofold change of expression as compared to the time-matched control (saline-treated) were referred to as responder genes (RG) and included into further analysis. The transcription factors that could potentially govern the expression of the RGs were identified using the position weight matrix method. To this end, the transcription factor binding sites in the promoter region (−1,500 to +500 bp relative to the transcriptional start site) of the RGs were found. For this, the coordinates of the transcription start site of each investigated gene were identified by 'biomaRt' package in the R environment using the coordinates of the 5′UTR of the most abundant mRNA isoform evaluated by Cufflinks (v. 2.2.1, NIH) basing on the RNA sequencing data of Peyer’s patch CD^4+^ T-lymphocytes (Visekruna et al., 2019). The search of the potential transcription factors binding sites and binding site enrichment analysis in the promoter regions was performed for the up- and downregulated genes separately relative to a set of random 5,000 protein-encoding genes promoters using the GeneExplain platform (“Search enriched TBFS in tracks” function) and TRANSFAC v. 2020.2 database of transcription factors binding sites (TBFS) (Koschmann et al., 2015). Briefly, the algorithm searches for the TBFSs in the promoter region of the investigated and the reference genes. Then the number of promoter regions containing specific TBFS is calculated for the investigated and the reference genes, followed by statistical analysis with the exact Fisher’s test. During the TFBS search, the positional weight matrix (PWM) cut-off is optimized to maximize the adjusted fold enrichment (odds ratio with a 99% confidence interval). The transcription factors binding sites with the adjusted fold enrichment (odds ratio with a 99% confidence interval) of 1.1 and FDR <0.1 (Fisher’s test) were considered to be meaningful.

#### Gene Ontology Analysis

The functional enrichment of the responder genes was examined relative to the entire set of analyzed genes (248 genes) by the PANTHER Overrepresentation Test (Released 2020-07-28) tool using the GO biological process complete and PANTHER Pathways databases. Gene Ontology terms with *p* < 0.05 (Bonferroni corrected binomial test) were considered significantly enriched.

#### Prediction of Upstream Signaling Pathways and Master-Regulators

The transcription factor sets obtained for up- and downregulated genes for a given combination of the tested substance and time point were used as an input for analysis of upstream signaling pathways and master-regulators (GeneExplain platform with Transpath v. 2020.2 database and workflow “Find master regulators in networks”) as described in (Koschmann et al., 2015). The search was done with a maximum radius of 10 steps upstream of an input gene set, and potential master regulators filtered with cutoffs: score >0.2, FDR <0.05, and Z score >1.0 (where score reflects the degree of a regulator molecule connectivity with other molecules in the database and with the input list; Z-score reflects the specificity of each master molecule for the input list; FDR represents the probability of the molecule to occupy the observed or a higher rank by random chance; FDR and Z-score is calculated on 1,000 random results).

#### Materials and Reagents

Gentamicin, Concanavalin A, l-glutamine supplemented RPMI-1640, HBSS, 10 × PBS, and 1 M HEPES sodium salt solution were purchased from PanEco (Russia). Percoll^®^, Propidium iodide, β-D-Glucan, and Polyinosinic–polycytidylic acid potassium salt (Poly I:C) were purchased from Sigma-Aldrich (Russia). SYBRGreen was purchased from Lumiprobe (Russia). TRI REAGENT^®^ was purchased from Molecular Research Center, Inc. (United States). Fetal Bovine Serum was purchased from BioSera (France). Lentinan was purchased from Toronto Research Chemicals (Canada). RNeasy Micro Kit was purchased from Qiagen (United States). Kagocel was provided by NearMedic Plus (Russia).

## Data Availability

The original contributions presented in the study are included in the article/Supplementary Material, further inquiries can be directed to the corresponding author.
