# Supplementary material for: Gene Expression Pattern of Peyer’s Patch Lymphocytes Exposed to Kagocel Suggests Pattern-Recognition Receptors Mediate Its Action
Source: Front Pharmacol. 2021 Aug 3;12:679511. doi: 10.3389/fphar.2021.679511 (PMC8369352; doi:10.3389/fphar.2021.679511)
Supplement: Supplementary file 1 [file Table1.DOCX]

**Log2-fold change of inflammation-related genes expression**

| **Gene** | **ConA** | | | | | | | | | | **ConA + Poly I:C** | | | | | | | | | |
| --- | --- | --- | --- | --- | --- | --- | --- | --- | --- | --- | --- | --- | --- | --- | --- | --- | --- | --- | --- | --- |
|  | **24** | | | | | **72** | | | | | **24** | | | | | **72** | | | | |
|  | **BDGlu** | **Lent** | **Kag30** | **Kag100** | **Kag300** | **BDGlu** | **Lent** | **Kag30** | **Kag100** | **Kag300** | **BDGlu** | **Lent** | **Kag30** | **Kag100** | **Kag300** | **BDGlu** | **Lent** | **Kag30** | **Kag100** | **Kag300** |
| *Ager* | 0.6 | -0.3 | -0.2 | -0.5 | -1.0 | 1.2 | 2.4 | 0.4 | 1.1 | 0.7 | 1.8 | -0.1 | -0.1 | -0.3 | -0.5 | 2.4 | 2.7 | 1.1 | 1.1 | 0.4 |
| *Alox5* | -2.0 | 0.5 | -1.3 | -0.2 | -0.4 | 0.4 | 1.4 | 0.5 | 0.3 | 0.3 | 1.7 | 0.9 | 0.6 | -0.5 | -3.5 | -0.2 | 2.1 | 0.7 | 0.7 | -0.9 |
| *Areg* | 0.0 | 1.7 | 0.0 | 0.0 | 0.0 | 0.0 | 0.0 | 0.0 | 4.2 | 3.0 | -2.1 | -0.1 | 1.3 | -2.1 | -2.1 | 0.0 | 0.0 | 2.0 | 1.7 | 2.2 |
| *Atf2* | -0.4 | -0.4 | -0.2 | -0.4 | -0.6 | 0.0 | 0.1 | 0.5 | -0.6 | -0.6 | 0.0 | 0.1 | 0.0 | 0.1 | 0.1 | 0.4 | 0.5 | -0.1 | 0.1 | -0.3 |
| *Bcl2l1* | -0.1 | -0.3 | -0.2 | -0.4 | -0.7 | 0.0 | 0.9 | 0.3 | 0.0 | -0.4 | 0.3 | 0.2 | 0.0 | 0.0 | 0.0 | 0.8 | 1.0 | 0.2 | 0.3 | 0.0 |
| *Bcl6* | -0.4 | -0.5 | -0.4 | 0.1 | -0.1 | -7.0 | -0.1 | 1.1 | -1.4 | -1.3 | -0.2 | 0.0 | 0.0 | 0.0 | -0.2 | -6.8 | -0.6 | -2.0 | -0.3 | -3.2 |
| *Birc2* | 0.0 | -0.1 | 0.0 | -0.1 | -0.3 | 0.1 | 0.5 | 0.7 | -0.3 | -0.3 | 0.4 | 0.0 | 0.0 | 0.0 | 0.0 | 0.9 | 0.7 | 0.0 | 0.2 | -0.2 |
| *C1qb* | -0.1 | -5.4 | -5.4 | -5.4 | -5.4 | 0.0 | 6.5 | 0.0 | 0.0 | 0.0 | 5.8 | 2.4 | 3.1 | 0.0 | 0.0 | 7.1 | 5.8 | 2.0 | 2.6 | 0.7 |
| *C1s* | -0.4 | -1.0 | -0.8 | -0.3 | -0.2 | -6.4 | 1.2 | -0.9 | -1.2 | -1.0 | -0.2 | -0.7 | 0.0 | 0.0 | 0.0 | 1.7 | 1.5 | 1.3 | 0.6 | 0.7 |
| *C2* | 0.9 | -1.2 | -0.6 | -2.8 | -3.1 | 6.8 | 7.0 | 5.1 | 0.8 | 0.0 | 5.4 | 1.7 | 0.6 | -1.1 | -1.8 | 6.8 | 5.2 | 2.3 | 2.6 | 1.6 |
| *C3* | 2.0 | -0.8 | -1.9 | -3.6 | -5.0 | 6.9 | 7.2 | 0.0 | 3.0 | 4.6 | 7.5 | 3.5 | 3.9 | 2.3 | 2.1 | 8.4 | 6.3 | 4.5 | 3.7 | 2.4 |
| *C4a* | 0.2 | -2.0 | -1.5 | -4.5 | -5.2 | 0.8 | 3.5 | 1.6 | -1.6 | -3.6 | 4.2 | -1.8 | 0.8 | -0.5 | 1.5 | 3.3 | 3.1 | -0.3 | 1.0 | -3.5 |
| *Ccl17* | -0.2 | -0.2 | -5.6 | -0.3 | -0.9 | 0.3 | 2.0 | 0.3 | 0.5 | -0.3 | 1.3 | 1.1 | 1.2 | -1.7 | -1.8 | -0.2 | 1.6 | -0.2 | -0.2 | -1.6 |
| *Ccl2* | -1.5 | -4.0 | -4.0 | -1.8 | -4.0 | 1.3 | 1.5 | 1.3 | 1.5 | 1.5 | 2.6 | -0.8 | 1.5 | -1.1 | -1.1 | -0.1 | 2.0 | -0.6 | 1.2 | -1.6 |
| *Ccl20* | -0.2 | -0.5 | -0.5 | 0.1 | -1.3 | -4.7 | 1.8 | 0.7 | -1.4 | -1.7 | -0.9 | -0.5 | -0.4 | -0.7 | 0.0 | 1.3 | 2.0 | 0.1 | 0.6 | -3.7 |
| *Ccl21a* | 1.8 | -0.3 | -2.2 | -4.0 | -3.0 | 7.6 | 8.0 | 0.0 | 0.0 | 0.0 | 7.9 | 4.8 | 4.2 | 3.1 | 3.3 | 8.4 | 7.7 | 5.3 | 4.3 | 3.1 |
| *Ccl22* | -0.5 | -0.3 | -0.4 | 0.0 | -0.6 | -0.7 | -0.5 | -1.8 | -0.9 | -1.6 | -0.5 | 0.5 | 0.0 | 0.1 | 0.3 | 0.1 | 0.1 | 0.7 | 0.2 | 0.6 |
| *Ccl3* | 0.1 | -0.1 | -0.1 | -0.2 | -0.6 | 0.0 | 0.7 | 0.4 | -0.7 | -0.8 | 0.0 | 0.1 | -0.1 | -0.2 | -0.2 | 0.8 | 1.0 | 0.5 | 0.3 | 0.0 |
| *Ccl4* | 0.1 | 0.4 | -0.1 | -0.2 | -0.4 | -0.1 | 0.5 | -0.1 | 0.3 | 0.4 | 0.2 | 0.2 | -0.1 | -0.1 | -0.2 | -1.2 | 0.3 | 0.2 | -0.1 | 0.1 |
| *Ccl5* | -0.1 | 0.3 | 0.4 | 0.0 | -0.4 | 0.3 | 1.8 | 0.7 | -0.1 | 0.0 | -0.2 | 0.2 | 0.0 | 0.1 | -0.1 | 0.8 | 2.0 | 0.1 | 0.2 | -0.4 |
| *Ccr1* | -4.1 | -1.6 | -1.1 | -4.1 | -4.1 | -4.7 | 0.6 | -0.9 | 0.0 | -0.2 | 1.6 | 0.2 | 0.3 | -1.3 | -3.0 | -6.0 | -1.0 | -0.8 | -0.3 | 0.0 |
| *Ccr2* | 0.3 | -0.6 | -0.4 | -1.1 | -1.6 | 0.5 | -0.4 | 0.0 | 1.1 | 1.4 | 0.8 | -0.3 | 0.8 | 0.3 | 0.9 | 0.0 | -0.9 | -0.4 | -0.3 | -0.1 |
| *Ccr4* | -0.3 | 0.1 | 0.1 | 0.3 | 0.5 | 0.1 | 0.8 | -0.2 | 0.3 | 0.3 | -0.7 | 0.0 | 0.0 | -0.2 | 0.0 | 0.5 | 0.8 | 0.4 | 0.2 | 0.1 |
| *Ccr7* | -0.3 | -0.4 | -0.6 | -0.8 | -1.3 | 0.1 | 1.2 | 1.2 | -1.2 | -1.4 | 0.9 | 0.7 | 0.1 | 0.1 | 0.0 | 0.9 | 1.6 | -0.6 | 0.4 | -1.3 |
| *Cd4* | -0.2 | 0.1 | 0.1 | -0.1 | -0.3 | 0.2 | 1.0 | 0.4 | 0.0 | 0.0 | -0.2 | 0.0 | -0.1 | -0.1 | 0.0 | 0.5 | 1.0 | 0.2 | 0.1 | -0.4 |
| *Cd40* | 0.1 | -0.2 | 0.0 | -0.3 | -0.6 | -3.1 | 0.2 | 0.6 | -1.7 | -2.2 | 0.5 | 0.2 | 0.0 | 0.3 | 0.1 | 0.5 | 0.3 | 0.1 | 0.2 | 0.0 |
| *Cd40lg* | -0.3 | -0.2 | -0.5 | -0.7 | -0.9 | -0.2 | 1.3 | 0.6 | -0.7 | -1.0 | 0.4 | 0.5 | 0.2 | 0.2 | 0.1 | 1.4 | 1.6 | 0.6 | 0.4 | -0.3 |
| *Cd55* | -0.4 | -0.4 | -0.4 | -0.4 | -0.9 | 0.6 | 0.8 | 0.9 | 0.6 | 0.7 | -0.1 | 0.2 | 0.0 | -0.1 | -0.1 | 1.3 | 1.3 | 0.0 | 0.2 | -0.7 |
| *Cd86* | 0.1 | -0.1 | 0.0 | -0.3 | -0.7 | -2.3 | -0.5 | 0.6 | -1.5 | -1.8 | 0.2 | 0.0 | 0.0 | -0.1 | 0.1 | 0.0 | -0.2 | 0.2 | 0.3 | -0.1 |
| *Cdc42* | -0.3 | -0.3 | -0.2 | -0.3 | -0.6 | 0.0 | 0.2 | 0.4 | 0.2 | 0.2 | 0.0 | 0.1 | 0.1 | 0.0 | 0.1 | 0.3 | 0.3 | -0.1 | 0.3 | -0.1 |
| *Cebpb* | 0.7 | 0.4 | 0.4 | 0.3 | 0.5 | 0.3 | 0.4 | -0.4 | 0.5 | 0.4 | 0.5 | -0.2 | -0.1 | 0.0 | -0.1 | 0.5 | 0.2 | 0.2 | 0.0 | 0.0 |
| *Cfl1* | 0.0 | -0.1 | 0.0 | 0.0 | 0.0 | -0.2 | -0.2 | 0.0 | 0.2 | 0.3 | -0.2 | -0.1 | 0.0 | 0.0 | 0.0 | 0.0 | -0.4 | -0.1 | 0.0 | 0.0 |
| *Creb1* | -1.6 | -0.7 | -0.9 | -0.6 | -1.3 | -4.9 | -0.1 | 0.5 | -0.8 | -1.0 | 0.9 | 0.0 | 0.2 | -0.5 | -0.3 | -4.3 | 1.1 | 0.4 | 0.5 | 0.1 |
| *Csf1* | 0.5 | 0.1 | 0.8 | 0.2 | 0.0 | 0.1 | -0.2 | 0.9 | -0.3 | -0.4 | 0.2 | -0.1 | -0.1 | 0.0 | 0.0 | 0.6 | 0.0 | -0.5 | 0.2 | -0.8 |
| *Csf2* | -0.3 | -0.2 | -0.6 | -0.7 | -0.9 | -7.3 | 0.1 | -0.1 | -0.5 | -1.0 | 1.1 | 0.8 | 0.7 | 0.1 | 0.4 | -0.2 | 0.8 | -0.1 | 0.4 | -0.3 |
| *Cxcl10* | 2.2 | 2.2 | 1.9 | 1.6 | 0.9 | -4.5 | 1.9 | 0.0 | -0.7 | -1.9 | 1.0 | 1.0 | 0.1 | 0.1 | 0.0 | 1.4 | 2.9 | 1.8 | 1.5 | 0.6 |
| *Cxcr4* | -0.4 | -0.1 | -0.6 | -0.1 | -0.6 | 0.0 | -0.1 | 1.1 | -0.7 | -0.7 | 0.4 | 0.5 | 0.0 | 0.1 | 0.0 | 0.1 | 0.0 | -1.3 | -0.1 | -0.9 |
| *Cysltr1* | -1.6 | -0.3 | -5.2 | -0.3 | -0.2 | -7.6 | -1.8 | -1.7 | 0.4 | 0.7 | -1.4 | -0.5 | -0.9 | -0.5 | -0.1 | -8.4 | -2.6 | -0.6 | 0.0 | 0.8 |
| *Cysltr2* | -0.3 | -0.4 | 0.0 | -0.3 | -1.5 | 0.0 | 0.5 | -0.3 | -0.7 | -0.4 | 0.9 | -0.8 | -0.1 | -0.2 | -0.2 | 0.5 | 0.6 | 0.5 | 0.1 | 0.0 |
| *Daxx* | -0.1 | -0.2 | -0.3 | -0.2 | -0.2 | -1.9 | 0.4 | 0.4 | -0.2 | -0.1 | -0.7 | 0.0 | 0.4 | 0.0 | 0.1 | -1.0 | 0.4 | -0.2 | 0.3 | -0.1 |
| *Ddit3* | -0.6 | -0.6 | -0.4 | -0.5 | -0.9 | 0.0 | 0.6 | 0.9 | -0.1 | -0.3 | 0.2 | -0.1 | 0.0 | 0.0 | 0.0 | 0.6 | 1.0 | -0.2 | 0.2 | -0.5 |
| *Defa-rs1* | -1.6 | -4.6 | -6.3 | -6.3 | -6.3 | 0.0 | 6.5 | 0.0 | 0.0 | 0.0 | 5.8 | 0.9 | 0.5 | 0.0 | 0.0 | 0.0 | 6.0 | 2.9 | 3.2 | 2.8 |
| *Elk1* | -1.1 | -0.7 | -0.6 | -0.6 | -0.2 | -4.9 | 1.2 | 0.1 | -0.8 | -0.7 | -1.3 | -0.3 | 0.2 | 0.3 | -0.2 | 0.4 | 0.7 | 0.4 | 0.4 | 0.2 |
| *Fasl* | -0.4 | -0.2 | -0.7 | -0.3 | -0.6 | -4.5 | 1.0 | 0.2 | -0.9 | 0.0 | -1.4 | 2.5 | 3.1 | 2.3 | 0.0 | -3.5 | 2.2 | -1.1 | 0.0 | 0.0 |
| *Fos* | -0.8 | -1.0 | -1.0 | -0.8 | -1.0 | 0.8 | 0.6 | 0.9 | 0.3 | 0.6 | 0.3 | 0.1 | 0.0 | 0.1 | 0.0 | 0.2 | 1.5 | 0.0 | 0.5 | -0.3 |
| *Gnaq* | -0.6 | -1.0 | -0.4 | -0.6 | -1.0 | -0.5 | 0.0 | 0.6 | -0.2 | -0.3 | 0.3 | -0.1 | 0.3 | 0.1 | 0.1 | 0.7 | 0.1 | -0.3 | 0.1 | -0.2 |
| *Gnas* | -0.1 | -0.2 | -0.1 | -0.1 | -0.2 | 0.0 | -0.1 | 0.3 | 0.0 | 0.1 | 0.0 | 0.0 | 0.0 | 0.0 | 0.0 | 0.2 | -0.1 | -0.1 | -0.1 | -0.2 |
| *Gnb1* | -0.2 | -0.3 | 0.0 | -0.2 | -0.3 | -0.1 | 0.1 | 0.3 | -0.2 | -0.1 | 0.0 | 0.0 | 0.0 | 0.0 | 0.1 | 0.2 | 0.2 | -0.1 | 0.0 | -0.2 |
| *Grb2* | -0.1 | -0.2 | -0.1 | -0.3 | -0.5 | -0.3 | -0.2 | 1.0 | -0.6 | -0.5 | 0.3 | 0.1 | -0.1 | 0.1 | 0.2 | 0.3 | 0.0 | -0.5 | 0.1 | -0.5 |
| *H2-Ea-ps* | -0.2 | -0.5 | -0.4 | -0.5 | -0.8 | -0.8 | -1.1 | 1.4 | -1.9 | -2.0 | 0.4 | 0.1 | 0.0 | 0.1 | 0.0 | 0.1 | -0.6 | -1.8 | 0.2 | -1.8 |
| *H2-Eb1* | -0.5 | -0.1 | -0.4 | -0.4 | -0.6 | -0.9 | -1.1 | 1.3 | -1.7 | -1.7 | 0.5 | 0.3 | 0.0 | 0.0 | 0.0 | -0.3 | -0.7 | -1.6 | 0.1 | -1.7 |
| *Hdac4* | -0.3 | -0.3 | 0.0 | -0.4 | -0.5 | 0.8 | 1.0 | 0.5 | 0.2 | 0.2 | 0.6 | 0.0 | 0.0 | 0.1 | 0.0 | 0.7 | 0.7 | 0.1 | -0.1 | -0.5 |
| *Hif1a* | -0.1 | -0.2 | 0.0 | -0.2 | -0.4 | 0.1 | 0.1 | 0.1 | 0.1 | 0.2 | -0.2 | -0.1 | -0.1 | 0.0 | 0.0 | 0.3 | 0.3 | 0.2 | 0.3 | 0.3 |
| *Hmgb1* | -0.2 | -0.5 | -0.1 | -0.1 | -0.2 | -0.1 | -0.6 | 0.2 | 0.2 | 0.3 | -0.3 | -0.2 | 0.0 | 0.1 | 0.2 | 0.0 | -0.5 | -0.1 | 0.1 | 0.1 |
| *Hmgb2* | 0.6 | 0.7 | 0.7 | -0.1 | 0.4 | -0.6 | 0.7 | 0.5 | 0.7 | 0.6 | 1.5 | 0.8 | 0.4 | 0.5 | 0.8 | 0.0 | 1.0 | -0.1 | 0.5 | 0.7 |
| *Hmgn1* | 0.1 | -0.5 | -0.5 | -0.5 | -0.5 | 1.5 | 0.8 | 0.3 | -0.3 | -0.3 | 0.8 | 0.3 | 0.1 | 0.1 | 0.1 | 2.1 | 1.0 | 0.4 | 0.3 | 0.0 |
| *Hras1* | 1.7 | -1.0 | 0.3 | -2.6 | -3.9 | 7.9 | 7.9 | 0.0 | 0.0 | 1.3 | 3.9 | 0.5 | 1.4 | 1.3 | 0.4 | 5.8 | 4.3 | 2.7 | 1.8 | 1.4 |
| *Hsh2d* | -0.5 | -0.3 | -0.2 | -0.6 | -0.1 | -5.1 | 1.7 | 0.3 | 0.2 | 0.3 | 0.0 | 0.2 | 0.5 | 0.4 | 0.6 | 1.4 | 1.5 | -0.1 | 0.0 | -0.1 |
| *Hspb1* | 1.9 | -0.9 | -1.3 | -5.3 | -5.3 | 6.0 | 5.7 | 1.5 | -2.1 | -2.1 | 5.2 | 1.9 | 1.6 | -2.1 | -2.1 | 8.6 | 7.5 | 4.2 | 4.5 | 3.6 |
| *Ifi27l2a* | 0.2 | 0.0 | 0.1 | 0.0 | -0.4 | -0.1 | 0.2 | 0.5 | 0.2 | 0.1 | 0.0 | 0.2 | 0.1 | 0.1 | -0.1 | 0.3 | 0.5 | -0.2 | 0.1 | -0.3 |
| *Ifi44* | 0.6 | 1.2 | -0.9 | -0.4 | -0.4 | -3.1 | 2.1 | 0.5 | -3.1 | -3.1 | 0.2 | -0.6 | -0.3 | -0.5 | -1.1 | -4.0 | 0.1 | -0.5 | -1.0 | -0.5 |
| *Ifit1* | -4.8 | -1.2 | -0.4 | -1.0 | -3.8 | -3.9 | 2.1 | 0.6 | -0.4 | -0.9 | 1.1 | 0.3 | 0.6 | 0.0 | -0.3 | -3.5 | 1.5 | -1.1 | -0.2 | -0.7 |
| *Ifit2* | 0.3 | 0.3 | 0.3 | 0.1 | -0.4 | 0.0 | 0.9 | 0.7 | 0.3 | 0.1 | 0.4 | 0.4 | 0.2 | 0.4 | 0.5 | 1.3 | 1.1 | 0.2 | 0.4 | -0.3 |
| *Ifit3* | 1.8 | -0.6 | -1.1 | -2.9 | -2.5 | 6.0 | 5.3 | 1.0 | -0.6 | -3.5 | 4.9 | 1.9 | 1.1 | 1.5 | 1.2 | 9.6 | 8.4 | 6.0 | 5.5 | 4.9 |
| *Ifna1* | 0.9 | -1.5 | -1.8 | -4.7 | -6.3 | 10.4 | 9.5 | 0.0 | 0.0 | 0.0 | 7.2 | 3.0 | 3.1 | 2.7 | 2.8 | 10.8 | 9.3 | 6.9 | 6.5 | 5.5 |
| *Ifng* | 0.2 | -0.3 | -0.3 | -0.4 | -0.7 | 0.3 | 1.2 | -0.6 | -0.2 | -0.8 | 1.6 | 0.7 | 0.6 | 0.5 | 0.1 | 2.0 | 1.7 | 1.2 | 0.7 | 0.5 |
| *Iigp1* | 0.3 | 0.2 | 0.5 | 0.4 | -0.1 | 0.0 | 0.3 | 0.9 | -0.6 | -0.8 | -0.3 | -0.1 | 0.0 | 0.1 | 0.0 | 0.7 | 0.6 | -0.2 | 0.2 | -0.8 |
| *Il10* | 0.9 | 1.1 | 1.4 | 1.5 | 1.3 | -0.8 | -0.9 | -1.7 | 0.4 | 0.5 | -1.2 | 0.4 | 0.1 | -0.2 | -0.2 | -1.5 | -1.9 | -0.4 | -0.1 | 0.4 |
| *Il10rb* | -0.1 | -0.4 | -0.5 | -0.3 | -0.7 | -0.3 | 0.6 | 1.0 | -0.3 | -0.3 | 0.5 | 0.3 | 0.1 | 0.1 | 0.2 | 0.5 | 0.8 | -0.4 | 0.2 | -0.6 |
| *Il12a* | -0.5 | -0.5 | -0.8 | -0.8 | -1.1 | -5.4 | -0.9 | 1.7 | -3.7 | -5.4 | -1.3 | 0.3 | -0.3 | 0.0 | 0.2 | -4.7 | -1.4 | -4.7 | 0.6 | -1.7 |
| *Il13* | 0.0 | -0.1 | -2.4 | -0.6 | -0.2 | -0.4 | -1.1 | -3.3 | 0.5 | 0.4 | 0.3 | -0.2 | 0.4 | 0.1 | -1.7 | -1.0 | -1.3 | 0.0 | 0.0 | 0.5 |
| *Il17a* | -3.5 | 0.0 | -3.5 | -3.5 | -3.5 | -3.3 | 1.9 | -3.3 | -3.3 | -3.3 | 1.1 | 0.8 | 0.1 | -2.1 | -3.0 | -0.9 | 4.6 | -0.9 | -0.4 | -0.9 |
| *Il18rap* | -1.4 | -0.9 | -0.9 | -1.2 | -1.3 | -5.7 | 0.8 | 0.5 | -0.7 | -0.1 | -0.4 | -0.6 | -0.2 | -0.9 | -0.5 | 2.3 | 1.5 | 0.4 | 1.1 | 0.0 |
| *Il1b* | -1.5 | -0.3 | -0.5 | -1.6 | -1.8 | 0.9 | 3.4 | -2.7 | 1.0 | 0.5 | 0.9 | -0.3 | 0.1 | -2.5 | -1.0 | 4.1 | 3.7 | 2.2 | 2.4 | 0.7 |
| *Il1r1* | -0.8 | -1.3 | -1.3 | -1.1 | -1.9 | -6.6 | 0.5 | 1.0 | -1.6 | -1.2 | 0.3 | -0.2 | 0.2 | -0.3 | -0.8 | -5.9 | 0.9 | -0.7 | 0.4 | -0.5 |
| *Il1rap* | -0.5 | -0.7 | 0.0 | -0.6 | -0.5 | -6.8 | 0.6 | 0.5 | -0.5 | -0.7 | 0.5 | 0.5 | 0.1 | -0.1 | 0.1 | -1.6 | 1.3 | 0.3 | 0.3 | -0.5 |
| *Il2* | -0.1 | -0.1 | -0.1 | -0.6 | -1.2 | 0.0 | 1.5 | 0.7 | -2.8 | -3.3 | 0.2 | 0.7 | 0.0 | 0.1 | -0.1 | 1.1 | 2.0 | 0.1 | 0.3 | -1.3 |
| *Il21* | 0.3 | 0.3 | 0.7 | 0.1 | 0.0 | 0.2 | 1.2 | -0.6 | -0.2 | -0.3 | 0.0 | 0.1 | -0.3 | -0.1 | -0.1 | 1.1 | 1.5 | 0.9 | 0.4 | 0.5 |
| *Il22* | 1.7 | 2.2 | -0.2 | 0.0 | 0.0 | -5.7 | 1.9 | -0.3 | -2.0 | -2.6 | 0.3 | 1.3 | -0.6 | -0.3 | -0.6 | -5.5 | 2.6 | 0.3 | 0.2 | -0.2 |
| *Il23a* | -1.3 | -3.7 | -5.4 | -4.0 | -4.4 | 0.0 | 6.6 | 0.0 | 0.8 | 0.0 | 6.3 | 3.5 | 2.4 | 0.0 | 0.0 | 6.4 | 5.8 | 3.5 | 4.1 | 0.0 |
| *Il23r* | -1.4 | -1.3 | -1.0 | -1.5 | -1.8 | -0.4 | -0.2 | 1.3 | -1.2 | -2.0 | 0.3 | 0.4 | 0.4 | -0.2 | -0.7 | 1.5 | 0.7 | -1.6 | 0.4 | -2.9 |
| *Il4* | -0.3 | 0.0 | -0.2 | 0.0 | 0.1 | -0.2 | 0.3 | -0.9 | 0.4 | 0.1 | -0.2 | 0.3 | -0.4 | 0.1 | 0.0 | 0.5 | 0.4 | 0.5 | 0.2 | 0.6 |
| *Il5* | 0.0 | 3.7 | 3.1 | 4.7 | 4.9 | -5.0 | -5.0 | -5.0 | 1.3 | 1.1 | -4.0 | -1.1 | 0.3 | -1.8 | -4.0 | -6.2 | -3.2 | -1.3 | 0.1 | 0.9 |
| *Il6* | 1.2 | -0.2 | -1.1 | -1.3 | -2.5 | 1.4 | 2.3 | -1.0 | 0.8 | 0.5 | 3.3 | 1.4 | 1.3 | 0.7 | -0.7 | 4.0 | 3.2 | 1.1 | 0.9 | 0.2 |
| *Il6ra* | -0.2 | -0.1 | -0.4 | -0.2 | -0.1 | 0.4 | 0.9 | 0.3 | 0.2 | -0.1 | 0.6 | 0.2 | -0.2 | -0.2 | -0.1 | 0.3 | 1.4 | -0.1 | 0.2 | -0.2 |
| *Il9* | 0.8 | -0.2 | 0.0 | 0.0 | 0.1 | 2.1 | 2.9 | -2.3 | -0.7 | -0.9 | 1.8 | 0.0 | 0.4 | 0.3 | 0.1 | 2.2 | 2.6 | 0.9 | 0.5 | 0.1 |
| *Irf1* | -0.2 | -0.3 | -0.2 | -0.2 | -0.4 | 0.2 | 0.3 | 0.8 | -0.3 | -0.3 | 0.2 | 0.2 | 0.1 | 0.2 | 0.1 | 0.5 | 0.6 | -0.3 | 0.2 | -0.6 |
| *Irf3* | 0.7 | -0.7 | -1.5 | -1.2 | -1.1 | -2.7 | 3.8 | 0.2 | 0.5 | 0.1 | 2.3 | -0.1 | 0.8 | -0.2 | 0.1 | 4.3 | 2.7 | 1.1 | 0.6 | -0.2 |
| *Irf5* | 0.0 | -0.2 | -0.2 | -0.5 | -0.7 | -0.3 | 0.0 | 1.0 | -1.4 | -1.4 | 0.6 | 0.3 | 0.1 | 0.1 | -0.2 | 0.7 | 0.1 | -0.5 | 0.1 | -0.6 |
| *Irf7* | 1.4 | -0.3 | -0.8 | -0.9 | -2.2 | 3.3 | 2.9 | 1.2 | 0.3 | 0.2 | 2.9 | 0.1 | 0.6 | 0.3 | 0.3 | 3.9 | 2.6 | 0.7 | 0.7 | -0.1 |
| *Itgb2* | -0.1 | -0.1 | 0.0 | -0.2 | -0.2 | 0.2 | 0.1 | 0.2 | 0.2 | 0.4 | 0.1 | 0.0 | 0.0 | 0.1 | 0.0 | 0.1 | -0.1 | -0.1 | -0.1 | -0.1 |
| *Jun* | -0.1 | -0.2 | -0.1 | 0.0 | -0.2 | -0.1 | 0.0 | 0.9 | -1.0 | -1.0 | 0.0 | 0.0 | 0.1 | 0.1 | 0.0 | 0.4 | 0.4 | -1.0 | 0.1 | -1.0 |
| *Keap1* | 0.0 | -0.4 | -0.2 | -0.3 | -0.6 | 0.6 | 0.7 | 0.5 | -0.1 | 0.0 | 0.7 | -0.3 | 0.0 | 0.0 | -0.2 | 0.5 | 0.5 | -0.1 | 0.2 | 0.2 |
| *Kng1* | 1.0 | -1.4 | -1.4 | -5.8 | -5.8 | 7.5 | 6.9 | 0.0 | 0.0 | 0.0 | 6.1 | 1.5 | 1.9 | -1.4 | 1.3 | 7.8 | 6.4 | 3.9 | 3.3 | 2.6 |
| *Limk1* | 0.0 | 0.2 | 0.2 | -0.2 | -0.2 | -2.7 | 0.1 | 0.2 | -0.2 | -0.2 | -0.1 | 0.0 | 0.4 | 0.0 | -0.1 | 0.4 | 0.6 | 0.0 | 0.2 | -0.1 |
| *Lta* | -0.2 | 0.3 | -0.3 | 0.0 | 0.0 | 0.1 | 1.1 | 0.4 | -0.4 | -0.4 | 0.1 | 0.5 | 0.1 | 0.2 | 0.0 | 0.8 | 1.7 | 0.4 | 0.4 | -0.2 |
| *Ltb* | 0.0 | 0.0 | -0.2 | -0.2 | -0.4 | 0.4 | 0.6 | 0.5 | 0.4 | 0.4 | 0.1 | 0.3 | 0.0 | 0.0 | 0.1 | 0.6 | 0.9 | -0.1 | 0.2 | -0.3 |
| *Ltb4r1* | 0.5 | -0.8 | -0.6 | -1.5 | -2.5 | 1.4 | 1.2 | 0.2 | -0.4 | 0.2 | 3.2 | 0.9 | 0.8 | 0.5 | 0.1 | 1.5 | 0.0 | -0.2 | -0.3 | -0.2 |
| *Ltb4r2* | 2.7 | -0.7 | -2.4 | -3.6 | -5.4 | 8.8 | 8.3 | 0.0 | 0.0 | 0.0 | 6.7 | 3.4 | 2.6 | 2.7 | 0.8 | 9.1 | 7.8 | 4.6 | 4.5 | 4.4 |
| *Ly96* | -1.1 | -0.5 | -0.8 | -0.6 | -1.4 | -6.1 | 0.8 | 0.7 | -0.4 | -0.8 | -0.1 | 0.9 | 0.0 | 0.4 | -0.2 | -5.5 | 1.5 | -0.6 | 0.5 | -0.4 |
| *Maff* | 0.6 | -0.9 | -0.4 | -1.4 | -1.3 | 2.0 | 1.9 | 0.6 | -1.3 | -1.4 | 1.6 | 0.1 | 0.4 | 0.0 | 0.0 | 2.8 | 2.2 | 0.7 | 0.4 | -0.1 |
| *Mafg* | 0.5 | -0.4 | -0.4 | -0.2 | -0.7 | 1.0 | 1.3 | 0.4 | -0.9 | -0.8 | 1.3 | 0.4 | 0.3 | 0.3 | 0.3 | 2.0 | 1.4 | 0.5 | 0.4 | 0.1 |
| *Mafk* | 0.2 | -0.1 | -0.4 | -0.4 | -0.2 | 0.5 | 0.9 | 0.7 | -0.7 | -0.7 | 0.6 | -0.1 | 0.1 | -0.2 | -0.2 | 1.3 | 0.9 | 0.1 | -0.1 | -0.5 |
| *Map2k1* | -0.2 | -0.3 | -0.3 | -0.3 | -0.5 | -0.1 | 0.3 | 0.3 | -0.4 | -0.4 | 0.0 | 0.2 | 0.2 | 0.1 | 0.1 | 0.2 | 0.5 | -0.1 | 0.1 | 0.0 |
| *Map2k4* | -0.5 | -0.4 | -0.5 | -0.2 | -0.6 | -0.6 | 0.4 | 0.5 | -0.1 | -0.1 | -0.4 | 0.2 | -0.2 | 0.1 | 0.2 | 0.0 | 0.8 | 0.0 | 0.3 | 0.0 |
| *Map2k6* | -2.5 | 1.8 | 0.6 | 2.1 | 1.5 | -4.8 | -1.0 | -0.4 | 1.6 | 1.9 | -0.6 | -1.2 | -0.3 | -1.2 | -2.1 | -5.8 | -1.3 | -0.3 | -0.2 | 0.5 |
| *Map3k1* | -0.1 | -0.3 | -0.2 | -0.2 | -0.4 | -0.5 | -0.6 | 0.8 | -0.6 | -0.4 | -0.2 | 0.0 | 0.0 | 0.0 | 0.0 | 0.1 | 0.0 | -0.5 | 0.1 | -0.3 |
| *Map3k5* | -0.3 | -0.2 | 0.0 | -0.2 | -0.5 | -0.3 | -0.2 | 0.4 | 0.2 | 0.3 | 0.2 | 0.0 | 0.0 | 0.1 | 0.1 | -0.1 | -0.2 | -0.1 | 0.0 | 0.0 |
| *Map3k7* | -0.3 | -0.4 | 0.0 | -0.2 | -0.5 | 0.1 | 0.6 | 0.4 | -0.2 | -0.3 | 0.2 | 0.2 | 0.0 | 0.0 | 0.1 | 0.9 | 1.0 | 0.2 | 0.3 | -0.1 |
| *Map3k9* | 1.4 | -1.0 | -1.8 | -3.1 | -1.3 | 5.8 | 6.6 | 0.0 | 0.0 | 0.0 | 2.7 | 0.1 | 0.7 | -0.5 | -0.9 | 5.3 | 3.4 | 0.8 | 0.5 | -2.4 |
| *Mapk1* | -0.1 | -0.2 | -0.4 | -0.2 | -0.2 | -0.4 | 0.3 | 0.3 | 0.0 | 0.0 | 0.0 | 0.3 | 0.0 | 0.0 | -0.1 | -0.5 | 0.4 | -0.1 | 0.3 | 0.0 |
| *Mapk14* | 0.3 | 0.1 | -0.1 | 0.1 | -0.1 | -0.1 | 0.4 | 0.3 | 0.1 | 0.1 | 0.3 | 0.2 | 0.1 | 0.1 | 0.2 | 0.3 | 0.6 | 0.0 | 0.2 | 0.0 |
| *Mapk3* | 0.2 | 0.1 | -0.4 | -0.1 | -0.2 | 0.1 | 0.4 | 0.2 | 0.0 | 0.1 | 0.2 | 0.2 | 0.1 | 0.1 | -0.2 | 0.5 | 0.4 | 0.0 | 0.2 | 0.0 |
| *Mapk8* | -0.4 | -0.3 | -0.4 | -0.3 | -0.6 | -0.6 | 0.1 | 0.7 | -0.1 | -0.3 | 0.0 | 0.3 | 0.1 | 0.1 | 0.3 | -0.2 | 0.6 | -0.6 | 0.1 | -0.6 |
| *Mapkapk2* | -0.2 | -0.1 | -0.1 | -0.1 | -0.1 | 0.0 | 0.4 | 0.0 | -0.1 | 0.0 | -0.1 | -0.1 | -0.1 | -0.1 | 0.0 | 0.2 | 0.2 | 0.1 | 0.0 | -0.1 |
| *Masp2* | 1.8 | -0.5 | -0.2 | -0.8 | -0.6 | 2.9 | 2.6 | -0.9 | -0.8 | -0.8 | 1.7 | -0.7 | -0.1 | -0.3 | -0.4 | 3.5 | 2.3 | 1.0 | 0.4 | 0.4 |
| *Max* | 0.2 | 0.1 | 0.1 | 0.1 | 0.1 | 0.2 | 0.3 | 0.6 | -0.5 | -0.3 | 0.3 | 0.0 | -0.1 | 0.0 | 0.0 | -0.2 | 0.6 | -0.3 | 0.2 | -0.4 |
| *Mef2a* | -0.5 | -0.8 | -0.6 | -0.8 | -1.6 | -2.5 | -0.3 | 0.8 | -0.8 | -0.6 | 0.5 | 0.0 | 0.3 | -0.1 | -0.1 | 0.6 | 0.1 | -0.5 | 0.3 | -0.7 |
| *Mef2b* | 0.4 | -0.3 | 0.0 | 0.0 | 0.1 | -4.1 | 1.7 | 0.7 | -0.2 | -1.8 | 0.8 | 0.1 | 0.5 | 0.3 | 0.1 | 2.7 | 0.3 | -0.6 | 0.2 | -0.5 |
| *Mef2c_Mm* | -0.3 | -0.6 | -0.3 | -0.3 | -0.6 | -1.2 | -1.5 | 1.2 | -1.7 | -1.7 | 0.1 | 0.0 | 0.1 | 0.1 | 0.2 | -0.3 | -1.2 | -1.4 | 0.0 | -1.1 |
| *Mef2d* | 0.1 | 0.0 | 0.1 | 0.0 | 0.0 | 0.0 | 0.0 | 0.8 | -0.3 | -0.1 | 0.2 | -0.1 | -0.3 | -0.1 | 0.0 | 0.9 | 0.2 | -0.4 | 0.0 | -0.6 |
| *Mknk1* | 0.0 | 0.0 | -0.2 | -0.3 | 0.0 | -1.2 | -0.1 | 0.6 | -0.4 | -0.4 | 0.0 | 0.1 | 0.1 | 0.2 | 0.0 | -1.2 | 0.6 | -0.4 | 0.3 | 0.1 |
| *Mx1* | 0.7 | -1.7 | -1.6 | -2.9 | -3.3 | 5.0 | 3.9 | 0.0 | -0.1 | 0.1 | 4.4 | 1.1 | 0.9 | 0.6 | 0.1 | 4.9 | 3.5 | 1.8 | 1.1 | 0.7 |
| *Mx2* | 1.7 | -0.5 | -0.2 | -1.7 | -1.7 | 6.7 | 6.2 | -1.2 | 2.3 | 0.9 | 2.4 | -0.2 | 0.3 | -0.2 | -0.4 | 6.9 | 6.0 | 2.6 | 3.3 | 1.6 |
| *Myc* | 0.0 | -0.2 | 0.0 | -0.1 | -0.2 | -0.3 | 0.2 | -0.1 | -0.4 | -0.4 | -0.2 | -0.1 | -0.1 | 0.0 | 0.0 | 0.3 | 0.3 | 0.3 | 0.1 | 0.1 |
| *Myd88* | 0.1 | -0.3 | -0.3 | -0.3 | -0.4 | 0.6 | 0.5 | 0.4 | -0.3 | -0.2 | 0.3 | 0.0 | -0.2 | -0.2 | -0.1 | 1.1 | 0.5 | 0.0 | -0.1 | -0.3 |
| *Nfatc3* | -0.4 | -0.2 | -0.3 | -0.4 | -0.5 | -0.5 | 0.1 | 0.8 | -0.1 | 0.1 | 0.1 | -0.1 | 0.0 | -0.1 | 0.0 | -0.2 | 0.1 | -0.2 | 0.1 | -0.4 |
| *Nfe2l2* | -0.7 | -0.6 | -0.2 | -0.3 | -0.7 | -0.4 | 0.1 | 0.9 | -0.5 | -0.6 | -0.6 | -0.2 | -0.1 | -0.1 | 0.0 | 0.2 | 0.5 | -0.5 | 0.2 | -0.8 |
| *Nfkb1* | -0.1 | -0.2 | -0.2 | -0.2 | -0.3 | 0.1 | 0.3 | 0.5 | -0.2 | -0.1 | 0.1 | 0.1 | 0.0 | 0.0 | 0.0 | 0.2 | 0.4 | -0.1 | 0.0 | -0.2 |
| *Nod1* | 1.7 | -0.1 | -0.4 | -0.9 | -0.8 | 3.4 | 3.3 | -0.3 | -0.9 | 0.1 | 2.4 | 0.3 | 0.4 | 0.1 | 0.7 | 3.9 | 2.5 | 1.1 | 0.3 | 0.0 |
| *Nod2* | -2.1 | 0.2 | 0.0 | 0.3 | 0.5 | -4.9 | -1.1 | -0.5 | -0.1 | -0.5 | -0.2 | -0.3 | -0.4 | -0.5 | -0.3 | -5.2 | -1.1 | -0.5 | 0.3 | 0.2 |
| *Nr3c1* | -0.1 | -0.4 | -0.2 | -0.2 | -0.8 | -0.2 | 0.3 | 0.8 | -0.3 | -0.4 | 0.3 | 0.1 | 0.1 | 0.0 | 0.0 | 0.6 | 0.6 | -0.3 | 0.2 | -0.6 |
| *Oas1a* | 0.6 | 1.2 | 1.3 | 0.8 | 1.0 | -6.2 | -0.1 | 0.2 | -1.2 | -0.9 | -1.1 | -0.3 | 0.2 | 0.0 | 0.0 | -0.4 | 0.5 | -0.6 | 0.1 | -0.3 |
| *Oas2* | 1.7 | 0.6 | -2.9 | -2.9 | -2.9 | 0.0 | 6.1 | 0.0 | 0.0 | 0.0 | 3.2 | -0.5 | 0.7 | -2.2 | -0.5 | 6.4 | 5.0 | 0.0 | 2.3 | 2.4 |
| *Oasl1* | -0.7 | -0.9 | -2.1 | -1.0 | -2.5 | -0.7 | 2.6 | -0.7 | -0.6 | -3.3 | 1.2 | 0.7 | 0.6 | 0.3 | 0.8 | -3.2 | 2.9 | 1.8 | 0.7 | 0.3 |
| *Pdgfa* | 0.7 | -0.7 | -1.3 | -1.0 | -1.3 | 0.3 | 1.0 | 0.3 | -2.2 | -2.2 | 2.4 | 0.0 | 0.1 | -0.4 | -4.6 | 2.0 | 1.3 | 0.7 | 0.5 | 0.2 |
| *Ppp1r12b* | 0.0 | 4.6 | 0.0 | 4.4 | 4.8 | -5.0 | -0.1 | 0.1 | -0.5 | -0.6 | -3.5 | 0.6 | 0.4 | 0.5 | 1.4 | -4.5 | 0.9 | -0.1 | 0.4 | -0.2 |
| *Prkca* | -0.3 | -0.3 | -0.4 | -0.4 | -0.7 | -0.6 | 0.8 | 1.0 | -0.7 | -0.6 | 0.3 | 0.1 | -0.1 | -0.1 | 0.1 | 1.1 | 1.3 | 0.0 | 0.4 | -0.5 |
| *Prkcb* | -0.4 | -0.8 | -0.3 | -0.5 | -0.8 | 0.0 | -0.1 | 0.9 | -0.4 | -0.3 | 0.1 | -0.1 | 0.0 | 0.1 | 0.1 | 0.3 | 0.3 | -0.3 | 0.2 | -0.6 |
| *Ptger2* | -0.3 | -0.4 | -0.2 | -0.2 | -0.5 | 0.0 | 0.7 | 0.1 | -0.3 | -0.5 | 0.4 | 0.0 | 0.0 | 0.0 | 0.0 | 0.9 | 1.1 | 0.4 | 0.3 | -0.2 |
| *Ptger4* | -0.2 | -0.2 | -0.3 | -0.4 | -0.6 | 0.2 | 0.7 | 0.5 | -0.4 | -0.3 | 0.7 | 0.4 | 0.0 | 0.1 | 0.0 | 1.0 | 0.8 | 0.1 | 0.0 | -0.1 |
| *Ptgir* | 1.4 | 0.3 | -1.5 | -0.9 | -0.9 | 1.5 | 1.1 | -0.5 | 0.4 | 0.3 | 1.9 | 0.2 | 0.6 | 0.2 | 0.1 | 1.8 | 0.5 | 0.2 | -0.1 | 0.0 |
| *Ptgs1* | 1.7 | -0.6 | -0.7 | -1.0 | -0.9 | 2.4 | 2.8 | -3.0 | -1.0 | -3.7 | 2.7 | -1.0 | 0.2 | -0.4 | -1.2 | 2.1 | 1.6 | 0.1 | 0.0 | -0.1 |
| *Ptk2* | -0.7 | -0.6 | -0.3 | -0.7 | -0.2 | -6.1 | 0.4 | 0.8 | -0.7 | -0.9 | 0.7 | 0.3 | 0.3 | 0.2 | 0.4 | -5.5 | 0.2 | -0.3 | 0.5 | 0.1 |
| *Rac1* | -0.1 | -0.3 | 0.0 | -0.3 | -0.3 | -0.1 | -0.2 | 0.2 | 0.0 | 0.1 | -0.1 | -0.1 | -0.1 | 0.0 | 0.1 | 0.0 | 0.0 | 0.0 | 0.1 | 0.1 |
| *Raf1* | -0.1 | -0.4 | 0.0 | 0.0 | -0.4 | -0.6 | 0.2 | 0.4 | -0.3 | -0.3 | 0.0 | 0.1 | 0.1 | 0.0 | 0.1 | 0.4 | 0.5 | -0.2 | 0.2 | -0.2 |
| *Rapgef2* | -0.7 | -0.6 | -1.2 | -1.0 | -1.4 | 1.2 | 0.9 | 0.4 | 0.4 | 0.4 | 0.9 | 0.3 | 0.1 | 0.0 | 0.2 | 1.4 | 0.9 | -0.1 | 0.2 | -0.1 |
| *Rela* | -0.2 | -0.1 | -0.2 | 0.0 | -0.4 | -0.3 | 0.5 | 0.5 | -0.4 | -0.4 | -0.1 | 0.1 | 0.0 | 0.1 | 0.0 | 0.1 | 0.7 | -0.2 | 0.1 | -0.3 |
| *Relb* | 0.0 | -0.4 | -0.3 | -0.4 | -0.6 | 0.4 | 0.6 | 0.7 | -0.1 | -0.2 | 0.2 | 0.1 | 0.0 | 0.0 | -0.1 | 0.9 | 1.0 | -0.1 | 0.2 | -0.4 |
| *Rhoa* | 0.1 | -0.4 | -0.2 | -0.3 | -0.4 | 0.5 | 0.0 | 0.6 | 0.0 | 0.0 | 0.5 | 0.1 | 0.1 | 0.2 | 0.3 | 0.8 | 0.2 | 0.0 | 0.0 | -0.2 |
| *Ripk1* | -0.4 | -0.5 | -0.3 | -0.7 | -0.8 | 1.5 | 0.8 | 0.7 | 0.0 | 0.3 | 0.4 | 0.0 | -0.2 | 0.0 | 0.3 | 1.8 | 0.6 | -0.4 | 0.0 | -0.1 |
| *Ripk2* | 0.2 | -0.2 | -0.1 | -0.1 | -0.6 | -1.0 | 0.0 | 0.4 | -0.8 | -0.5 | 0.0 | -0.1 | 0.0 | 0.0 | -0.2 | -0.2 | 0.0 | 0.0 | 0.1 | 0.2 |
| *Rock2* | -0.3 | 0.0 | 0.0 | -0.1 | -0.3 | -0.4 | 0.4 | 0.5 | -0.4 | -0.2 | 0.4 | 0.1 | 0.0 | 0.0 | -0.1 | -0.6 | 0.2 | -0.2 | -0.1 | -0.2 |
| *Rps6ka5* | -0.4 | -0.6 | -1.9 | -0.7 | -0.7 | 0.7 | 0.2 | 0.3 | -0.2 | 0.2 | 0.5 | 0.0 | 0.2 | -0.4 | -0.7 | -0.2 | -0.7 | -0.6 | 0.1 | 0.2 |
| *Shc1* | -0.3 | -0.2 | 0.1 | 0.0 | -0.1 | -1.0 | -0.1 | 0.3 | 0.1 | 0.1 | 0.1 | -0.2 | 0.0 | 0.1 | 0.0 | -0.7 | -0.1 | -0.2 | 0.0 | -0.3 |
| *Smad7* | 0.0 | -0.1 | -0.4 | -0.4 | -0.7 | 0.3 | 1.0 | 0.6 | -0.2 | -0.1 | 1.1 | 0.4 | 0.0 | 0.0 | -0.1 | 1.4 | 1.1 | 0.1 | 0.3 | -0.1 |
| *Stat1* | -0.2 | -0.3 | -0.2 | -0.2 | -0.5 | 0.2 | 0.2 | 0.9 | -0.1 | -0.2 | 0.2 | 0.2 | 0.1 | 0.1 | 0.1 | 0.6 | 0.6 | -0.2 | 0.2 | -0.6 |
| *Stat2* | 0.1 | 0.1 | 0.4 | 0.1 | -0.2 | -0.4 | 0.2 | 0.7 | -0.7 | -0.6 | 0.0 | -0.1 | -0.1 | -0.1 | 0.0 | 0.2 | 0.2 | -0.2 | 0.1 | -0.6 |
| *Stat3* | 0.0 | -0.1 | -0.1 | -0.2 | -0.2 | -0.2 | 0.1 | 0.4 | -0.5 | -0.4 | -0.1 | 0.1 | 0.1 | 0.1 | 0.1 | -0.1 | 0.3 | -0.1 | 0.2 | 0.0 |
| *Tbxa2r* | 1.7 | -0.6 | -0.3 | -1.5 | -2.0 | 4.3 | 4.1 | -0.5 | 0.4 | 0.8 | 2.9 | 0.3 | 0.2 | -0.1 | 0.1 | 4.6 | 3.2 | 1.6 | 0.6 | 0.4 |
| *Tcf4* | -0.4 | -0.7 | -0.5 | -0.6 | -1.0 | -1.0 | -1.3 | 1.0 | -1.6 | -1.5 | 0.2 | 0.0 | 0.0 | 0.1 | 0.0 | -0.2 | -0.6 | -0.9 | 0.1 | -0.5 |
| *Tgfb1* | 0.0 | 0.0 | 0.0 | -0.2 | -0.1 | 0.0 | 0.1 | 0.2 | 0.1 | 0.1 | 0.2 | 0.1 | 0.0 | 0.0 | -0.1 | 0.1 | 0.2 | 0.0 | 0.1 | -0.1 |
| *Tgfb3* | 0.5 | -0.4 | -0.7 | 0.1 | -0.7 | -0.8 | 0.5 | 1.0 | -0.5 | -0.1 | 1.6 | 0.2 | 0.0 | 0.0 | 0.1 | 1.2 | -0.2 | -1.2 | 0.1 | -0.1 |
| *Tgfbr1* | -0.1 | -0.5 | -0.5 | -0.5 | -0.8 | 0.1 | 0.7 | 0.6 | 0.0 | 0.1 | 0.5 | 0.0 | 0.0 | 0.1 | 0.0 | 1.4 | 0.9 | -0.2 | 0.0 | -0.3 |
| *Tlr1* | 0.0 | 0.0 | -0.3 | -0.1 | -0.6 | -0.4 | 0.4 | 0.9 | 0.3 | 0.4 | 0.0 | 0.1 | -0.1 | 0.0 | -0.1 | -0.3 | 0.5 | -0.3 | 0.2 | -0.3 |
| *Tlr2* | 1.8 | -0.6 | 0.0 | -1.2 | -1.8 | 5.0 | 4.9 | 1.1 | 0.4 | -0.4 | 3.1 | 0.6 | 1.3 | 0.1 | 0.3 | 5.7 | 5.2 | 2.9 | 2.6 | 2.2 |
| *Tlr4* | -0.6 | -0.6 | -0.5 | -1.1 | -1.2 | -6.5 | 0.1 | 1.0 | -1.0 | -1.1 | 0.9 | 0.5 | 0.2 | 0.3 | 0.6 | -6.6 | 0.2 | -1.2 | 0.4 | 0.0 |
| *Tlr6* | 0.0 | 0.0 | -0.1 | -0.2 | -0.6 | -0.1 | 0.4 | 1.1 | 0.0 | 0.2 | 0.7 | 0.2 | 0.4 | 0.4 | 0.4 | 1.0 | 0.5 | -0.7 | 0.2 | -0.6 |
| *Tlr7* | 0.2 | -0.1 | 0.4 | 0.1 | -0.3 | -0.9 | -0.7 | 1.4 | -1.2 | -1.4 | -0.1 | 0.1 | 0.0 | 0.1 | 0.1 | -0.3 | -0.7 | -1.5 | 0.2 | -0.9 |
| *Tlr9* | -0.2 | -0.1 | -0.2 | -0.4 | -0.5 | -0.6 | -0.4 | 1.6 | -1.5 | -1.7 | 0.5 | 0.5 | 0.1 | 0.1 | 0.1 | 0.2 | 0.4 | -2.8 | 0.3 | -2.5 |
| *Tnf* | -0.2 | 0.0 | -0.4 | -0.5 | -0.4 | -0.4 | 0.6 | 0.3 | -0.3 | -0.2 | 0.4 | 0.1 | -0.1 | 0.0 | -0.1 | 1.1 | 0.8 | 0.4 | 0.3 | -0.2 |
| *Tnfaip3* | -0.2 | -0.6 | -0.4 | -0.5 | -0.7 | 0.2 | 0.4 | 0.6 | -0.7 | -0.5 | 0.2 | 0.0 | 0.0 | 0.1 | 0.0 | 0.5 | 0.4 | -0.3 | -0.1 | -0.3 |
| *Tnfsf14* | -0.3 | -0.5 | -0.1 | -0.7 | -1.2 | 1.1 | 1.0 | 0.2 | -0.5 | -0.4 | 1.1 | 0.3 | 0.1 | 0.1 | 0.4 | 1.2 | 1.3 | 0.6 | 0.3 | -0.2 |
| *Tollip* | -0.2 | -0.2 | -0.2 | -0.4 | -0.6 | 0.1 | 0.5 | 0.5 | -0.4 | -0.3 | 0.2 | 0.0 | 0.0 | 0.0 | 0.0 | 0.7 | 0.8 | -0.2 | 0.3 | -0.2 |
| *Tradd* | 0.9 | -0.1 | -0.1 | -0.3 | -0.5 | 1.7 | 1.7 | 0.2 | -0.2 | -0.2 | 1.3 | 0.5 | 0.3 | 0.0 | 0.1 | 1.7 | 1.3 | 0.4 | 0.1 | 0.0 |
| *Traf2* | -0.5 | -0.1 | 0.2 | -0.3 | -0.3 | -0.5 | 0.5 | 0.3 | -0.3 | -0.2 | 0.0 | 0.0 | 0.0 | 0.0 | 0.1 | 0.1 | 0.3 | -0.1 | 0.0 | -0.4 |
| *Tyrobp* | 1.2 | -0.3 | -0.4 | -0.9 | -1.3 | 2.3 | 2.0 | 1.1 | 0.0 | 0.0 | 2.0 | 0.2 | 0.1 | 0.2 | 0.2 | 3.1 | 2.2 | 0.4 | 0.7 | -0.3 |
