# Supplementary figures and images for: Gene Expression Pattern of Peyer’s Patch Lymphocytes Exposed to Kagocel Suggests Pattern-Recognition Receptors Mediate Its Action
Source: Front Pharmacol. 2021 Aug 3;12:679511. doi: 10.3389/fphar.2021.679511 (PMC8369352; doi:10.3389/fphar.2021.679511)

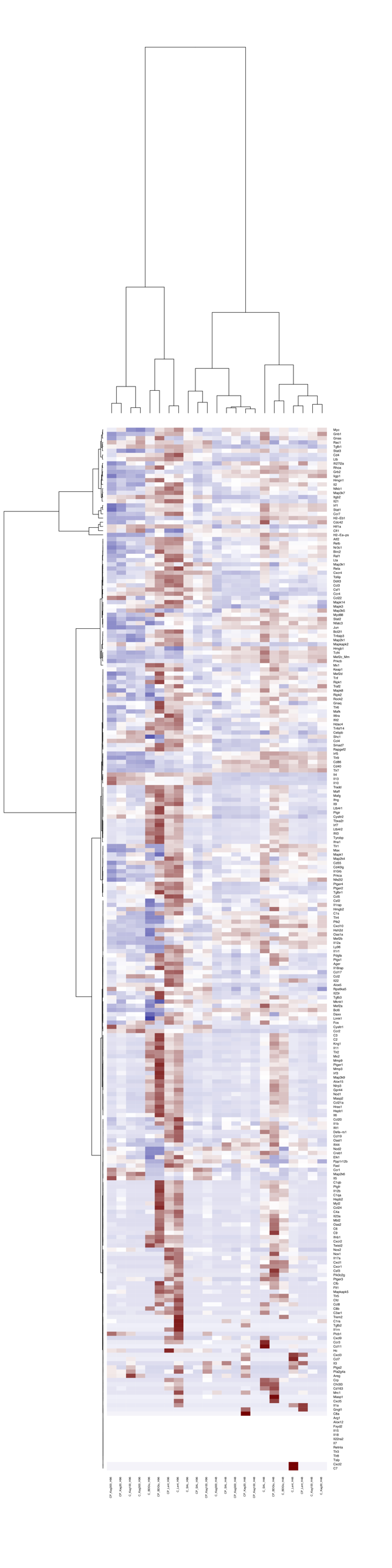

Supplement: Supplementary file 2 [file Image1.JPEG]
