# Supplementary material for: Gene Expression Pattern of Peyer’s Patch Lymphocytes Exposed to Kagocel Suggests Pattern-Recognition Receptors Mediate Its Action
Source: Front Pharmacol. 2021 Aug 3;12:679511. doi: 10.3389/fphar.2021.679511 (PMC8369352; doi:10.3389/fphar.2021.679511)
Supplement: Supplementary file 3 [file Table2.DOCX]

**List of transcriptional factors identified with GeneExplain Transfac**

| **Mitogen** | **Drug** | **Time** | **Transcription factor** | **Adj seq FE** | **Seq FDR** |
| --- | --- | --- | --- | --- | --- |
| ConA | BDGlu | 24 | Cebpe | 1.1196 | 0.0234 |
| ConA | BDGlu | 24 | Elf3 | 1.2137 | 0.0342 |
| ConA | BDGlu | 24 | Elf5 | 1.2751 | 0.0177 |
| ConA | BDGlu | 24 | Fos | 1.1236 | 0.0394 |
| ConA | BDGlu | 24 | Fosl2 | 1.2058 | 0.0355 |
| ConA | BDGlu | 24 | Glis3 | 1.1000 | 0.0410 |
| ConA | BDGlu | 24 | Hsf2 | 1.1175 | 0.0358 |
| ConA | BDGlu | 24 | Irf2 | 1.9277 | 0.0295 |
| ConA | BDGlu | 24 | Jund | 1.4897 | 0.0313 |
| ConA | BDGlu | 24 | Lef1 | 1.2913 | 0.0215 |
| ConA | BDGlu | 24 | Mafb | 1.4389 | 0.0410 |
| ConA | BDGlu | 24 | Mafg | 1.2958 | 0.0358 |
| ConA | BDGlu | 24 | Nfe2 | 1.9672 | 0.0128 |
| ConA | BDGlu | 24 | Nfe2l2 | 1.4749 | 0.0410 |
| ConA | BDGlu | 24 | Nfkb2 | 1.2972 | 0.0394 |
| ConA | BDGlu | 24 | Nkx2-3 | 1.2929 | 0.0179 |
| ConA | BDGlu | 24 | Rara | 1.1422 | 0.0280 |
| ConA | BDGlu | 24 | Rora | 1.2913 | 0.0331 |
| ConA | BDGlu | 24 | Spi1 | 1.2502 | 0.0443 |
| ConA | BDGlu | 24 | Stat3 | 1.3318 | 0.0410 |
| ConA | BDGlu | 24 | Tal1 | 1.1409 | 0.0313 |
| ConA | BDGlu | 24 | Tcf3 | 1.1409 | 0.0313 |
| ConA | BDGlu | 72 | Arid5b | 1.3985 | 0.0564 |
| ConA | BDGlu | 72 | Atf5 | 1.2410 | 0.0421 |
| ConA | BDGlu | 72 | Foxm1 | 1.2321 | 0.0541 |
| ConA | BDGlu | 72 | Foxo1 | 1.1838 | 0.0541 |
| ConA | BDGlu | 72 | Gfi1 | 1.5641 | 0.0818 |
| ConA | BDGlu | 72 | Gfi1b | 1.5641 | 0.0818 |
| ConA | BDGlu | 72 | Hand1 | 1.2282 | 0.0667 |
| ConA | BDGlu | 72 | Hsf1 | 1.3397 | 0.0498 |
| ConA | BDGlu | 72 | Irf1 | 1.5924 | 0.0660 |
| ConA | BDGlu | 72 | Irf2 | 1.5924 | 0.0660 |
| ConA | BDGlu | 72 | Irf3 | 1.5924 | 0.0660 |
| ConA | BDGlu | 72 | Irf3 | 1.5424 | 0.0541 |
| ConA | BDGlu | 72 | Irf4 | 1.5924 | 0.0660 |
| ConA | BDGlu | 72 | Irf5 | 1.5924 | 0.0660 |
| ConA | BDGlu | 72 | Irf6 | 1.5924 | 0.0660 |
| ConA | BDGlu | 72 | Irf7 | 1.5924 | 0.0660 |
| ConA | BDGlu | 72 | Irf8 | 1.5924 | 0.0660 |
| ConA | BDGlu | 72 | Irf8 | 1.3294 | 0.0541 |
| ConA | BDGlu | 72 | Mafk | 1.3755 | 0.0924 |
| ConA | BDGlu | 72 | Nr2e1 | 1.3448 | 0.0588 |
| ConA | BDGlu | 72 | Rbpj | 1.1231 | 0.0584 |
| ConA | BDGlu | 72 | Relb | 1.3765 | 0.0584 |
| ConA | BDGlu | 72 | Spib | 1.3262 | 0.0541 |
| ConA | BDGlu | 72 | Stat3 | 1.2349 | 0.0885 |
| ConA | BDGlu | 72 | Zbtb7a | 1.3188 | 0.0847 |
| ConA + Poly I:C | BDGlu | 24 | Bach1 | 1.1492 | 0.0259 |
| ConA + Poly I:C | BDGlu | 24 | Cebpe | 1.5465 | 0.0305 |
| ConA + Poly I:C | BDGlu | 24 | Esr1 | 2.3449 | 0.0053 |
| ConA + Poly I:C | BDGlu | 24 | Esr2 | 1.1825 | 0.0357 |
| ConA + Poly I:C | BDGlu | 24 | Foxl2 | 1.1849 | 0.0380 |
| ConA + Poly I:C | BDGlu | 24 | Gfi1 | 1.1863 | 0.0275 |
| ConA + Poly I:C | BDGlu | 24 | Glis3 | 1.1456 | 0.0237 |
| ConA + Poly I:C | BDGlu | 24 | Hand1 | 1.1229 | 0.0224 |
| ConA + Poly I:C | BDGlu | 24 | Hdx | 1.4932 | 0.0286 |
| ConA + Poly I:C | BDGlu | 24 | Hnf1a | 1.3718 | 0.0300 |
| ConA + Poly I:C | BDGlu | 24 | Irf1 | 1.4645 | 0.0067 |
| ConA + Poly I:C | BDGlu | 24 | Irf2 | 1.4645 | 0.0067 |
| ConA + Poly I:C | BDGlu | 24 | Irf3 | 1.4645 | 0.0067 |
| ConA + Poly I:C | BDGlu | 24 | Irf4 | 2.2733 | 0.0066 |
| ConA + Poly I:C | BDGlu | 24 | Irf5 | 1.4645 | 0.0067 |
| ConA + Poly I:C | BDGlu | 24 | Irf6 | 1.4645 | 0.0067 |
| ConA + Poly I:C | BDGlu | 24 | Irf7 | 1.4645 | 0.0067 |
| ConA + Poly I:C | BDGlu | 24 | Irf8 | 1.8379 | 0.0118 |
| ConA + Poly I:C | BDGlu | 24 | Mafb | 1.4785 | 0.0097 |
| ConA + Poly I:C | BDGlu | 24 | Mafk | 1.5506 | 0.0088 |
| ConA + Poly I:C | BDGlu | 24 | Nfe2 | 1.6159 | 0.0260 |
| ConA + Poly I:C | BDGlu | 24 | Nfe2l2 | 1.5003 | 0.0153 |
| ConA + Poly I:C | BDGlu | 24 | Nfkb1 | 1.5353 | 0.0151 |
| ConA + Poly I:C | BDGlu | 24 | Nfkb2 | 1.2439 | 0.0207 |
| ConA + Poly I:C | BDGlu | 24 | Rarg | 1.7920 | 0.0067 |
| ConA + Poly I:C | BDGlu | 24 | Rela | 1.7446 | 0.0067 |
| ConA + Poly I:C | BDGlu | 24 | Spib | 1.2734 | 0.0250 |
| ConA + Poly I:C | BDGlu | 24 | Stat1 | 1.3801 | 0.0260 |
| ConA + Poly I:C | BDGlu | 24 | Stat2 | 1.3801 | 0.0260 |
| ConA + Poly I:C | BDGlu | 24 | Stat3 | 1.3801 | 0.0260 |
| ConA + Poly I:C | BDGlu | 24 | Stat4 | 1.3801 | 0.0260 |
| ConA + Poly I:C | BDGlu | 24 | Stat5a | 1.3801 | 0.0260 |
| ConA + Poly I:C | BDGlu | 24 | Stat5b | 1.3801 | 0.0260 |
| ConA + Poly I:C | BDGlu | 24 | Stat6 | 1.3801 | 0.0260 |
| ConA + Poly I:C | BDGlu | 24 | Thra | 1.4290 | 0.0235 |
| ConA + Poly I:C | BDGlu | 24 | Zfp182 | 1.4603 | 0.0380 |
| ConA + Poly I:C | BDGlu | 24 | Zscan4c | 1.4590 | 0.0153 |
| ConA + Poly I:C | BDGlu | 24 | Zscan4d | 1.4590 | 0.0153 |
| ConA + Poly I:C | BDGlu | 24 | Zscan4f | 1.4590 | 0.0153 |
| ConA + Poly I:C | BDGlu | 72 | Cebpe | 1.5097 | 0.0530 |
| ConA + Poly I:C | BDGlu | 72 | Cphx1 | 1.3229 | 0.0520 |
| ConA + Poly I:C | BDGlu | 72 | Esr1 | 1.8109 | 0.0417 |
| ConA + Poly I:C | BDGlu | 72 | Hsf1 | 1.4123 | 0.0354 |
| ConA + Poly I:C | BDGlu | 72 | Irf1 | 1.4303 | 0.0198 |
| ConA + Poly I:C | BDGlu | 72 | Irf2 | 1.4303 | 0.0198 |
| ConA + Poly I:C | BDGlu | 72 | Irf2 | 1.1035 | 0.0467 |
| ConA + Poly I:C | BDGlu | 72 | Irf3 | 1.4303 | 0.0198 |
| ConA + Poly I:C | BDGlu | 72 | Irf3 | 1.1167 | 0.0467 |
| ConA + Poly I:C | BDGlu | 72 | Irf4 | 1.6291 | 0.0457 |
| ConA + Poly I:C | BDGlu | 72 | Irf5 | 1.4303 | 0.0198 |
| ConA + Poly I:C | BDGlu | 72 | Irf6 | 1.4303 | 0.0198 |
| ConA + Poly I:C | BDGlu | 72 | Irf7 | 1.4303 | 0.0198 |
| ConA + Poly I:C | BDGlu | 72 | Irf8 | 1.4686 | 0.0494 |
| ConA + Poly I:C | BDGlu | 72 | Mafk | 1.3672 | 0.0543 |
| ConA + Poly I:C | BDGlu | 72 | Nfkb1 | 1.2438 | 0.0459 |
| ConA + Poly I:C | BDGlu | 72 | Nfkb2 | 1.1269 | 0.0520 |
| ConA + Poly I:C | BDGlu | 72 | Nr1h4 | 1.1661 | 0.0576 |
| ConA + Poly I:C | BDGlu | 72 | Nr1h5 | 1.1661 | 0.0576 |
| ConA + Poly I:C | BDGlu | 72 | Rarg | 1.4780 | 0.0417 |
| ConA + Poly I:C | BDGlu | 72 | Rela | 1.3051 | 0.0494 |
| ConA + Poly I:C | BDGlu | 72 | Runx2 | 1.2772 | 0.0494 |
| ConA + Poly I:C | BDGlu | 72 | Runx3 | 1.2772 | 0.0494 |
| ConA + Poly I:C | BDGlu | 72 | Rxra | 1.1661 | 0.0576 |
| ConA + Poly I:C | BDGlu | 72 | Srf | 1.1239 | 0.0263 |
| ConA + Poly I:C | BDGlu | 72 | Stat1 | 1.3475 | 0.0505 |
| ConA + Poly I:C | BDGlu | 72 | Stat2 | 1.3475 | 0.0505 |
| ConA + Poly I:C | BDGlu | 72 | Stat3 | 1.3475 | 0.0505 |
| ConA + Poly I:C | BDGlu | 72 | Stat4 | 1.3475 | 0.0505 |
| ConA + Poly I:C | BDGlu | 72 | Stat5a | 1.3475 | 0.0505 |
| ConA + Poly I:C | BDGlu | 72 | Stat5b | 1.3475 | 0.0505 |
| ConA + Poly I:C | BDGlu | 72 | Stat6 | 1.3475 | 0.0505 |
| ConA + Poly I:C | BDGlu | 72 | T | 1.1715 | 0.0354 |
| ConA + Poly I:C | BDGlu | 72 | Tcf7l1 | 1.4780 | 0.0553 |
| ConA + Poly I:C | BDGlu | 72 | Tcf7l2 | 1.6912 | 0.0473 |
| ConA + Poly I:C | BDGlu | 72 | Zfp24 | 1.3579 | 0.0467 |
| ConA + Poly I:C | BDGlu | 72 | Zfp354a | 1.5265 | 0.0417 |
| ConA + Poly I:C | BDGlu | 72 | Zfp37 | 1.2497 | 0.0467 |
| ConA | Kag100 | 24 | Arid5a | 1.1422 | 0.0186 |
| ConA | Kag100 | 24 | Cebpe | 1.3731 | 0.0131 |
| ConA | Kag100 | 24 | Cux1 | 1.6709 | 0.0164 |
| ConA | Kag100 | 24 | Esr1 | 1.3935 | 0.0164 |
| ConA | Kag100 | 24 | Esr2 | 1.1331 | 0.0131 |
| ConA | Kag100 | 24 | Hdx | 1.3179 | 0.0187 |
| ConA | Kag100 | 24 | Hnf1a | 1.5147 | 0.0223 |
| ConA | Kag100 | 24 | Hoxc9 | 1.2711 | 0.0131 |
| ConA | Kag100 | 24 | Irf1 | 1.2800 | 0.0243 |
| ConA | Kag100 | 24 | Irf2 | 1.2800 | 0.0243 |
| ConA | Kag100 | 24 | Irf3 | 1.2800 | 0.0243 |
| ConA | Kag100 | 24 | Irf4 | 1.2800 | 0.0243 |
| ConA | Kag100 | 24 | Irf5 | 1.5146 | 0.0242 |
| ConA | Kag100 | 24 | Irf6 | 1.2800 | 0.0243 |
| ConA | Kag100 | 24 | Irf7 | 1.2800 | 0.0243 |
| ConA | Kag100 | 24 | Irf8 | 1.2800 | 0.0243 |
| ConA | Kag100 | 24 | Mafg | 1.7176 | 0.0204 |
| ConA | Kag100 | 24 | Nfe2l1 | 1.7176 | 0.0204 |
| ConA | Kag100 | 24 | Nfe2l2 | 1.3832 | 0.0243 |
| ConA | Kag100 | 24 | Nfkb2 | 1.1416 | 0.0273 |
| ConA | Kag100 | 24 | Nkx2-9 | 1.2381 | 0.0281 |
| ConA | Kag100 | 24 | Nr1h2 | 1.1245 | 0.0273 |
| ConA | Kag100 | 24 | Nr1h4 | 1.4439 | 0.0188 |
| ConA | Kag100 | 24 | Nr1h5 | 1.4439 | 0.0188 |
| ConA | Kag100 | 24 | Rarg | 1.4640 | 0.0164 |
| ConA | Kag100 | 24 | Rela | 1.3438 | 0.0248 |
| ConA | Kag100 | 24 | Rxra | 1.4439 | 0.0188 |
| ConA | Kag100 | 24 | Srf | 1.1187 | 0.0223 |
| ConA | Kag100 | 24 | Thra | 1.1126 | 0.0235 |
| ConA | Kag100 | 72 | E4f1 | 1.1340 | 0.1648 |
| ConA + Poly I:C | Kag100 | 24 | Cebpa | 1.4928 | 0.0793 |
| ConA + Poly I:C | Kag100 | 24 | Cebpb | 1.3560 | 0.0793 |
| ConA + Poly I:C | Kag100 | 24 | Cebpd | 1.1797 | 0.1052 |
| ConA + Poly I:C | Kag100 | 24 | Stat5a | 1.1935 | 0.1357 |
| ConA + Poly I:C | Kag100 | 72 | Esr1 | 1.1817 | 0.0457 |
| ConA + Poly I:C | Kag100 | 72 | Esr2 | 1.1817 | 0.0457 |
| ConA + Poly I:C | Kag100 | 72 | Lef1 | 1.1177 | 0.0547 |
| ConA + Poly I:C | Kag100 | 72 | Nr1h4 | 1.5458 | 0.0424 |
| ConA + Poly I:C | Kag100 | 72 | Nr1h5 | 1.5458 | 0.0424 |
| ConA + Poly I:C | Kag100 | 72 | Onecut1 | 1.6548 | 0.0424 |
| ConA + Poly I:C | Kag100 | 72 | Onecut2 | 1.5178 | 0.0450 |
| ConA + Poly I:C | Kag100 | 72 | Pax2 | 1.5162 | 0.0424 |
| ConA + Poly I:C | Kag100 | 72 | Pparg | 1.1240 | 0.0355 |
| ConA + Poly I:C | Kag100 | 72 | Rxra | 1.5458 | 0.0424 |
| ConA + Poly I:C | Kag100 | 72 | Tead4 | 1.2797 | 0.0489 |
| ConA + Poly I:C | Kag100 | 72 | Zfp410 | 1.2736 | 0.0427 |
| ConA | Kag30 | 24 | Cebpe | 1.2799 | 0.0215 |
| ConA | Kag30 | 24 | Cux1 | 1.5076 | 0.0287 |
| ConA | Kag30 | 24 | Esr1 | 1.7555 | 0.0287 |
| ConA | Kag30 | 24 | Esr2 | 1.1016 | 0.0215 |
| ConA | Kag30 | 24 | Hdx | 1.4621 | 0.0396 |
| ConA | Kag30 | 24 | Hnf1a | 1.5127 | 0.0287 |
| ConA | Kag30 | 24 | Hoxc9 | 1.2027 | 0.0160 |
| ConA | Kag30 | 24 | Nr1h2 | 1.1019 | 0.0287 |
| ConA | Kag30 | 24 | Rarg | 1.8000 | 0.0193 |
| ConA | Kag30 | 24 | Rxra | 1.1019 | 0.0287 |
| ConA | Kag30 | 24 | Zfp354a | 1.4149 | 0.0425 |
| ConA + Poly I:C | Kag30 | 24 | Fezf1 | 1.2462 | 0.0130 |
| ConA + Poly I:C | Kag30 | 24 | Gcm1 | 1.2145 | 0.0295 |
| ConA + Poly I:C | Kag30 | 24 | Hoxc9 | 1.2482 | 0.0086 |
| ConA + Poly I:C | Kag30 | 24 | Irf1 | 1.2723 | 0.0361 |
| ConA + Poly I:C | Kag30 | 24 | Irf2 | 1.2839 | 0.0295 |
| ConA + Poly I:C | Kag30 | 24 | Irf3 | 1.2723 | 0.0361 |
| ConA + Poly I:C | Kag30 | 24 | Irf4 | 1.2723 | 0.0361 |
| ConA + Poly I:C | Kag30 | 24 | Irf5 | 1.7960 | 0.0133 |
| ConA + Poly I:C | Kag30 | 24 | Irf6 | 1.2723 | 0.0361 |
| ConA + Poly I:C | Kag30 | 24 | Irf7 | 1.2723 | 0.0361 |
| ConA + Poly I:C | Kag30 | 24 | Irf8 | 1.2723 | 0.0361 |
| ConA + Poly I:C | Kag30 | 24 | Nfkb1 | 1.4335 | 0.0328 |
| ConA + Poly I:C | Kag30 | 24 | Nr1h2 | 1.1633 | 0.0300 |
| ConA + Poly I:C | Kag30 | 24 | Pparg | 1.1214 | 0.0300 |
| ConA + Poly I:C | Kag30 | 24 | Rxra | 1.1633 | 0.0300 |
| ConA + Poly I:C | Kag30 | 24 | Stat1 | 1.5004 | 0.0300 |
| ConA + Poly I:C | Kag30 | 24 | Stat2 | 1.5004 | 0.0300 |
| ConA + Poly I:C | Kag30 | 24 | Stat3 | 1.5004 | 0.0300 |
| ConA + Poly I:C | Kag30 | 24 | Stat4 | 1.5004 | 0.0300 |
| ConA + Poly I:C | Kag30 | 24 | Stat5a | 1.5004 | 0.0300 |
| ConA + Poly I:C | Kag30 | 24 | Stat5b | 1.5004 | 0.0300 |
| ConA + Poly I:C | Kag30 | 24 | Stat6 | 1.5004 | 0.0300 |
| ConA + Poly I:C | Kag30 | 24 | Tead4 | 1.3296 | 0.0300 |
| ConA + Poly I:C | Kag30 | 24 | Zscan4c | 1.4420 | 0.0295 |
| ConA + Poly I:C | Kag30 | 24 | Zscan4d | 1.4420 | 0.0295 |
| ConA + Poly I:C | Kag30 | 24 | Zscan4f | 1.4420 | 0.0295 |
| ConA + Poly I:C | Kag30 | 72 | Ebf1 | 1.2490 | 0.0469 |
| ConA + Poly I:C | Kag30 | 72 | Fosl1 | 1.1212 | 0.0469 |
| ConA + Poly I:C | Kag30 | 72 | Fosl2 | 1.2121 | 0.0469 |
| ConA + Poly I:C | Kag30 | 72 | Gtf3a | 1.5616 | 0.0469 |
| ConA + Poly I:C | Kag30 | 72 | Hsf1 | 1.1038 | 0.0469 |
| ConA + Poly I:C | Kag30 | 72 | Hsf2 | 1.1396 | 0.0469 |
| ConA + Poly I:C | Kag30 | 72 | Mafk | 1.4714 | 0.0479 |
| ConA + Poly I:C | Kag30 | 72 | Nfe2 | 1.5861 | 0.0469 |
| ConA + Poly I:C | Kag30 | 72 | Nr1h4 | 1.5373 | 0.0469 |
| ConA + Poly I:C | Kag30 | 72 | Nr1h5 | 1.5373 | 0.0469 |
| ConA + Poly I:C | Kag30 | 72 | Prdm16 | 1.1443 | 0.0514 |
| ConA + Poly I:C | Kag30 | 72 | Rxra | 1.5373 | 0.0469 |
| ConA + Poly I:C | Kag30 | 72 | T | 1.3170 | 0.1878 |
| ConA + Poly I:C | Kag30 | 72 | Tead4 | 1.3682 | 0.0469 |
| ConA + Poly I:C | Kag30 | 72 | Zfp37 | 1.3577 | 0.1878 |
| ConA | Kag300 | 24 | Cebpb | 1.1186 | 0.0269 |
| ConA | Kag300 | 24 | Cebpe | 1.5465 | 0.0293 |
| ConA | Kag300 | 24 | Cux1 | 1.5014 | 0.0191 |
| ConA | Kag300 | 24 | Esr1 | 1.2254 | 0.0211 |
| ConA | Kag300 | 24 | Gata1 | 1.7084 | 0.0190 |
| ConA | Kag300 | 24 | Gata2 | 1.6529 | 0.0240 |
| ConA | Kag300 | 24 | Glis3 | 1.1190 | 0.0224 |
| ConA | Kag300 | 24 | Hand1 | 1.1023 | 0.0259 |
| ConA | Kag300 | 24 | Hdx | 1.4989 | 0.0206 |
| ConA | Kag300 | 24 | Hnf1a | 1.3718 | 0.0289 |
| ConA | Kag300 | 24 | Hoxc9 | 1.2443 | 0.0151 |
| ConA | Kag300 | 24 | Hsf2 | 1.1777 | 0.0151 |
| ConA | Kag300 | 24 | Irf1 | 1.3802 | 0.0178 |
| ConA | Kag300 | 24 | Irf2 | 1.3802 | 0.0178 |
| ConA | Kag300 | 24 | Irf3 | 1.3802 | 0.0178 |
| ConA | Kag300 | 24 | Irf4 | 1.3802 | 0.0178 |
| ConA | Kag300 | 24 | Irf5 | 1.3802 | 0.0178 |
| ConA | Kag300 | 24 | Irf6 | 1.3802 | 0.0178 |
| ConA | Kag300 | 24 | Irf7 | 1.3802 | 0.0178 |
| ConA | Kag300 | 24 | Irf8 | 1.3802 | 0.0178 |
| ConA | Kag300 | 24 | Mafg | 1.5210 | 0.0268 |
| ConA | Kag300 | 24 | Nfe2l1 | 1.5210 | 0.0268 |
| ConA | Kag300 | 24 | Nfe2l2 | 1.1517 | 0.0347 |
| ConA | Kag300 | 24 | Nfkb1 | 1.3187 | 0.0228 |
| ConA | Kag300 | 24 | Nfkb2 | 1.2284 | 0.0190 |
| ConA | Kag300 | 24 | Nkx2-9 | 1.1849 | 0.0362 |
| ConA | Kag300 | 24 | Nr1h2 | 1.1499 | 0.0191 |
| ConA | Kag300 | 24 | Nr1h4 | 1.2889 | 0.0190 |
| ConA | Kag300 | 24 | Nr1h5 | 1.2889 | 0.0190 |
| ConA | Kag300 | 24 | Rarg | 1.5139 | 0.0190 |
| ConA | Kag300 | 24 | Rela | 1.4093 | 0.0151 |
| ConA | Kag300 | 24 | Rxra | 1.2889 | 0.0190 |
| ConA | Kag300 | 24 | Srf | 1.1038 | 0.0211 |
| ConA | Kag300 | 24 | Zfp182 | 1.6519 | 0.0211 |
| ConA | Kag300 | 72 | Hsf1 | 1.1133 | 0.1655 |
| ConA | Kag300 | 72 | Rela | 1.1119 | 0.1477 |
| ConA + Poly I:C | Kag300 | 24 | Cebpa | 1.4928 | 0.0793 |
| ConA + Poly I:C | Kag300 | 24 | Cebpb | 1.3560 | 0.0793 |
| ConA + Poly I:C | Kag300 | 24 | Cebpd | 1.1797 | 0.1052 |
| ConA + Poly I:C | Kag300 | 24 | Stat5a | 1.1935 | 0.1357 |
| ConA + Poly I:C | Kag300 | 72 | Arid5a | 1.1256 | 0.1922 |
| ConA + Poly I:C | Kag300 | 72 | Hsf1 | 1.3374 | 0.2439 |
| ConA + Poly I:C | Kag300 | 72 | Zfy1 | 1.1678 | 0.1922 |
| ConA | Lent | 24 | Ar | 1.3682 | 0.0457 |
| ConA | Lent | 24 | Cebpa | 1.1612 | 0.0386 |
| ConA | Lent | 24 | Cebpb | 1.4216 | 0.0386 |
| ConA | Lent | 24 | Cebpe | 1.2061 | 0.0386 |
| ConA | Lent | 24 | Cux1 | 1.4371 | 0.0445 |
| ConA | Lent | 24 | Esr1 | 1.3081 | 0.0386 |
| ConA | Lent | 24 | Esr2 | 1.1817 | 0.0386 |
| ConA | Lent | 24 | Fezf1 | 1.1469 | 0.0523 |
| ConA | Lent | 24 | Irf1 | 1.9775 | 0.0065 |
| ConA | Lent | 24 | Irf2 | 1.8442 | 0.0122 |
| ConA | Lent | 24 | Irf3 | 1.1201 | 0.0225 |
| ConA | Lent | 24 | Irf4 | 1.1201 | 0.0225 |
| ConA | Lent | 24 | Irf5 | 1.1201 | 0.0225 |
| ConA | Lent | 24 | Irf5 | 1.6930 | 0.0386 |
| ConA | Lent | 24 | Irf7 | 1.1201 | 0.0225 |
| ConA | Lent | 24 | Irf8 | 1.1201 | 0.0225 |
| ConA | Lent | 24 | Irf9 | 1.1201 | 0.0225 |
| ConA | Lent | 24 | Lef1 | 1.4134 | 0.0387 |
| ConA | Lent | 24 | Msx1 | 1.1046 | 0.0387 |
| ConA | Lent | 24 | Nfe2l2 | 1.4493 | 0.0387 |
| ConA | Lent | 24 | Nfkb2 | 1.4162 | 0.0269 |
| ConA | Lent | 24 | Pknox1 | 1.3323 | 0.0386 |
| ConA | Lent | 24 | Pou2f2 | 1.1445 | 0.0401 |
| ConA | Lent | 24 | Rarg | 1.3545 | 0.0386 |
| ConA | Lent | 24 | Rela | 1.1421 | 0.0326 |
| ConA | Lent | 24 | Relb | 1.6097 | 0.0171 |
| ConA | Lent | 24 | Zscan4c | 1.5360 | 0.0387 |
| ConA | Lent | 24 | Zscan4d | 1.5360 | 0.0387 |
| ConA | Lent | 24 | Zscan4f | 1.5360 | 0.0387 |
| ConA | Lent | 72 | Bcl6b | 1.1089 | 0.0163 |
| ConA | Lent | 72 | Cebpe | 1.5891 | 0.0143 |
| ConA | Lent | 72 | Cux1 | 1.3060 | 0.0195 |
| ConA | Lent | 72 | Esr1 | 1.6708 | 0.0128 |
| ConA | Lent | 72 | Gata1 | 1.5963 | 0.0100 |
| ConA | Lent | 72 | Gata4 | 1.1086 | 0.0224 |
| ConA | Lent | 72 | Hand1 | 1.1283 | 0.0114 |
| ConA | Lent | 72 | Hdx | 1.4805 | 0.0151 |
| ConA | Lent | 72 | Irf1 | 1.8447 | 0.0054 |
| ConA | Lent | 72 | Irf2 | 1.8447 | 0.0054 |
| ConA | Lent | 72 | Irf3 | 1.8447 | 0.0054 |
| ConA | Lent | 72 | Irf4 | 2.4567 | 0.0044 |
| ConA | Lent | 72 | Irf5 | 1.8447 | 0.0054 |
| ConA | Lent | 72 | Irf6 | 1.8447 | 0.0054 |
| ConA | Lent | 72 | Irf7 | 1.8447 | 0.0054 |
| ConA | Lent | 72 | Irf8 | 1.8447 | 0.0054 |
| ConA | Lent | 72 | Mafb | 1.2619 | 0.0152 |
| ConA | Lent | 72 | Mafk | 1.4810 | 0.0111 |
| ConA | Lent | 72 | Nfe2l2 | 1.2976 | 0.0172 |
| ConA | Lent | 72 | Nfkb1 | 2.0536 | 0.0063 |
| ConA | Lent | 72 | Nfkb2 | 1.5099 | 0.0101 |
| ConA | Lent | 72 | Nr1h4 | 1.1521 | 0.0219 |
| ConA | Lent | 72 | Nr1h5 | 1.1521 | 0.0219 |
| ConA | Lent | 72 | Rarg | 1.4290 | 0.0150 |
| ConA | Lent | 72 | Rel | 1.6556 | 0.0113 |
| ConA | Lent | 72 | Rela | 2.1658 | 0.0032 |
| ConA | Lent | 72 | Rxra | 1.1521 | 0.0219 |
| ConA | Lent | 72 | Spi1 | 1.1411 | 0.0150 |
| ConA | Lent | 72 | Spib | 1.1929 | 0.0143 |
| ConA | Lent | 72 | Stat1 | 1.3016 | 0.0173 |
| ConA | Lent | 72 | Stat2 | 1.3016 | 0.0173 |
| ConA | Lent | 72 | Stat3 | 1.3016 | 0.0173 |
| ConA | Lent | 72 | Stat4 | 1.3016 | 0.0173 |
| ConA | Lent | 72 | Stat5a | 1.3016 | 0.0173 |
| ConA | Lent | 72 | Stat5b | 1.3016 | 0.0173 |
| ConA | Lent | 72 | Stat6 | 1.3016 | 0.0173 |
| ConA | Lent | 72 | Tal1 | 1.1270 | 0.0115 |
| ConA | Lent | 72 | Tcf3 | 1.1270 | 0.0115 |
| ConA | Lent | 72 | Tcf7 | 1.6392 | 0.0142 |
| ConA | Lent | 72 | Tcf7l1 | 1.8231 | 0.0101 |
| ConA | Lent | 72 | Tcf7l2 | 1.7873 | 0.0101 |
| ConA | Lent | 72 | Zscan4c | 1.2511 | 0.0219 |
| ConA | Lent | 72 | Zscan4d | 1.2511 | 0.0219 |
| ConA | Lent | 72 | Zscan4f | 1.2511 | 0.0219 |
| ConA + Poly I:C | Lent | 24 | Cebpb | 1.1642 | 0.0923 |
| ConA + Poly I:C | Lent | 24 | Ets1 | 1.1387 | 0.0923 |
| ConA + Poly I:C | Lent | 24 | Etv2 | 1.2867 | 0.0923 |
| ConA + Poly I:C | Lent | 24 | Fosl2 | 1.2084 | 0.0923 |
| ConA + Poly I:C | Lent | 24 | Hoxd13 | 1.1695 | 0.0923 |
| ConA + Poly I:C | Lent | 24 | Hsf1 | 1.1213 | 0.0923 |
| ConA + Poly I:C | Lent | 24 | Nr1h2 | 1.1886 | 0.0923 |
| ConA + Poly I:C | Lent | 24 | Rbpj | 1.1387 | 0.0923 |
| ConA + Poly I:C | Lent | 24 | Rxra | 1.1886 | 0.0923 |
| ConA + Poly I:C | Lent | 24 | Zfp410 | 1.4160 | 0.0923 |
| ConA + Poly I:C | Lent | 72 | Cebpd | 1.1698 | 0.0232 |
| ConA + Poly I:C | Lent | 72 | Cebpe | 2.2045 | 0.0062 |
| ConA + Poly I:C | Lent | 72 | Esr1 | 1.6236 | 0.0215 |
| ConA + Poly I:C | Lent | 72 | Gata1 | 1.5514 | 0.0164 |
| ConA + Poly I:C | Lent | 72 | Gata2 | 1.2211 | 0.0215 |
| ConA + Poly I:C | Lent | 72 | Gata4 | 1.4497 | 0.0152 |
| ConA + Poly I:C | Lent | 72 | Gata6 | 1.6348 | 0.0234 |
| ConA + Poly I:C | Lent | 72 | Hsf1 | 1.1783 | 0.0067 |
| ConA + Poly I:C | Lent | 72 | Hsf2 | 1.5883 | 0.0037 |
| ConA + Poly I:C | Lent | 72 | Irf2 | 1.4308 | 0.0168 |
| ConA + Poly I:C | Lent | 72 | Irf3 | 1.1623 | 0.0171 |
| ConA + Poly I:C | Lent | 72 | Irf4 | 1.5551 | 0.0267 |
| ConA + Poly I:C | Lent | 72 | Irf5 | 1.4299 | 0.0267 |
| ConA + Poly I:C | Lent | 72 | Irf8 | 1.4173 | 0.0189 |
| ConA + Poly I:C | Lent | 72 | Maf | 1.3046 | 0.0190 |
| ConA + Poly I:C | Lent | 72 | Mafb | 1.4671 | 0.0063 |
| ConA + Poly I:C | Lent | 72 | Mafk | 1.3196 | 0.0243 |
| ConA + Poly I:C | Lent | 72 | Nfe2l2 | 1.2612 | 0.0278 |
| ConA + Poly I:C | Lent | 72 | Nfkb1 | 1.5785 | 0.0037 |
| ConA + Poly I:C | Lent | 72 | Nfkb2 | 1.4430 | 0.0021 |
| ConA + Poly I:C | Lent | 72 | Rela | 1.7782 | 0.0109 |
| ConA + Poly I:C | Lent | 72 | Tcf7l1 | 1.5660 | 0.0267 |
| ConA + Poly I:C | Lent | 72 | Tfcp2l1 | 2.9567 | 0.0024 |
| ConA + Poly I:C | Lent | 72 | Thra | 1.3098 | 0.0245 |
| ConA + Poly I:C | Lent | 72 | Tlx2 | 1.2997 | 0.0295 |
