# Supplementary material for: Gene Expression Pattern of Peyer’s Patch Lymphocytes Exposed to Kagocel Suggests Pattern-Recognition Receptors Mediate Its Action
Source: Front Pharmacol. 2021 Aug 3;12:679511. doi: 10.3389/fphar.2021.679511 (PMC8369352; doi:10.3389/fphar.2021.679511)
Supplement: Supplementary file 4 [file Table3.DOCX]

**List of master regulators identified with GeneExplain Transpath**

| **Mitogen** | **Drug** | **Time** | **Transpath ID** | **Master molecule name** | **Score** | **FDR** | **Z-Score** | **Ranks sum** |
| --- | --- | --- | --- | --- | --- | --- | --- | --- |
| ConA | BDGlu | 48 | MO000089319 | Caspase(m) | 0.49203 | 0.021 | 2.3096 | 16 |
| ConA | BDGlu | 48 | MO000020145 | TRAF2(m) | 0.42849 | 0.002 | 2.5419 | 17 |
| ConA | BDGlu | 48 | MO000232618 | beta-TrCP1-isoform1(m) | 0.41216 | 0.016 | 3.2399 | 17 |
| ConA | BDGlu | 48 | MO000022202 | IRAK-1(m) | 0.3952 | 0.038 | 2.1357 | 30 |
| ConA | BDGlu | 48 | MO000086968 | HPK1(m) | 0.34694 | 0.009 | 1.9886 | 35 |
| ConA | BDGlu | 48 | MO000039080 | IRAK-4(m) | 0.28085 | 0.009 | 2.355 | 36 |
| ConA | BDGlu | 48 | MO000169114 | 20S proteasome(m) | 0.35071 | 0.034 | 1.8837 | 38 |
| ConA | BDGlu | 48 | MO000018297 | beta-TrCP1(m) | 0.49106 | 0.044 | 1.6264 | 39 |
| ConA | BDGlu | 48 | MO000057644 | Cdk5(m) | 0.35855 | 0.025 | 1.8335 | 39 |
| ConA | BDGlu | 48 | MO000234738 | Irak2(m) | 0.32473 | 0.032 | 1.8884 | 41 |
| ConA | BDGlu | 48 | MO000019250 | RIP(m) | 0.2777 | 0.017 | 2.1977 | 42 |
| ConA | BDGlu | 48 | MO000038316 | LPS:lbp:CD14:TLR4:MD-2:TIRAP:IRAK-2 | 0.41135 | 0.03 | 1.6811 | 42 |
| ConA | BDGlu | 48 | MO000041437 | dsRNA:TLR3:TRIF | 0.41135 | 0.03 | 1.6822 | 42 |
| ConA | BDGlu | 48 | MO000086646 | NF-kappaB2(m) | 0.23947 | 0.03 | 3.2521 | 42 |
| ConA | BDGlu | 48 | MO000036067 | TRAF3(m) | 0.30309 | 0.03 | 1.8659 | 44 |
| ConA | BDGlu | 48 | MO000119664 | IRAK-4(m) | 0.27096 | 0.019 | 2.2008 | 45 |
| ConA | BDGlu | 48 | MO000032846 | MKP-3(m) | 0.56194 | 0.023 | 1.3485 | 46 |
| ConA | BDGlu | 48 | MO000107968 | MKP-2(m) | 0.6028 | 0.018 | 1.302 | 47 |
| ConA | BDGlu | 48 | MO000165800 | IL-15Ralpha(m) | 0.25413 | 0.007 | 2.2004 | 47 |
| ConA | BDGlu | 48 | MO000176200 | JKAP(m) | 0.57927 | 0.014 | 1.3108 | 47 |
| ConA | BDGlu | 48 | MO000009397 | MEK2(m) | 0.56182 | 0.03 | 1.3274 | 48 |
| ConA | BDGlu | 48 | MO000093758 | TRAF2-isoform1(m) | 0.22692 | 0.003 | 2.4391 | 49 |
| ConA | BDGlu | 48 | MO000102207 | ERK2(m){p} | 0.44413 | 0.038 | 1.4384 | 49 |
| ConA | BDGlu | 48 | MO000008125 | Mos(m) | 0.56785 | 0.047 | 1.281 | 50 |
| ConA | BDGlu | 48 | MO000281381 | (angiotensin II)2:(AT2 receptor)2:(ATIP-isoform3)2:SHP-1 | 0.52563 | 0.025 | 1.3639 | 50 |
| ConA | BDGlu | 48 | MO000009387 | MEK1(m) | 0.68447 | 0.049 | 1.0555 | 51 |
| ConA | BDGlu | 48 | MO000121034 | CIKS(m) | 0.36143 | 0.049 | 1.5523 | 51 |
| ConA | BDGlu | 48 | MO000239976 | Ptpn7(m) | 0.53553 | 0.033 | 1.3159 | 51 |
| ConA | BDGlu | 48 | MO000099420 | TRADD(m) | 0.24629 | 0.014 | 1.9656 | 52 |
| ConA | BDGlu | 48 | MO000037578 | PU.1(m) | 0.20214 | 0.029 | 2.4631 | 53 |
| ConA | BDGlu | 48 | MO000004576 | EphA4(m) | 0.43082 | 0.05 | 1.3985 | 54 |
| ConA | BDGlu | 48 | MO000044588 | parkin:Cul-1:Fbw7 | 0.24462 | 0.013 | 1.951 | 54 |
| ConA | BDGlu | 48 | MO000054385 | Ret(m) | 0.27969 | 0.05 | 1.7481 | 55 |
| ConA | BDGlu | 48 | MO000281378 | (angiotensin II)2:(AT2 receptor)2:(ATIP-isoform3)2 | 0.30094 | 0.034 | 1.6682 | 55 |
| ConA | BDGlu | 48 | MO000131893 | AT2(m) | 0.39789 | 0.038 | 1.4353 | 56 |
| ConA | BDGlu | 48 | MO000171605 | MEK1(m){pS} | 0.54741 | 0.044 | 1.2481 | 56 |
| ConA | BDGlu | 48 | MO000281382 | (ATIP-isoform3)2:SHP-1 | 0.34533 | 0.042 | 1.5516 | 56 |
| ConA | BDGlu | 48 | MO000019413 | cd40(m) | 0.28174 | 0.043 | 1.6552 | 57 |
| ConA | BDGlu | 48 | MO000128058 | beta-TrCP1-isoform2(m) | 0.27426 | 0.032 | 1.7936 | 57 |
| ConA | BDGlu | 48 | MO000239971 | Ptpn5(m) | 0.53437 | 0.043 | 1.2681 | 57 |
| ConA | BDGlu | 48 | MO000280542 | (angiotensin II)2:(AT2 receptor)2 | 0.34529 | 0.044 | 1.5452 | 58 |
| ConA | BDGlu | 48 | MO000059832 | PTP-SL(m) | 0.46617 | 0.041 | 1.2806 | 60 |
| ConA | BDGlu | 48 | MO000285932 | Cezanne(m) | 0.2376 | 0.044 | 1.7483 | 64 |
| ConA | BDGlu | 48 | MO000019393 | IL-1RAcP(m) | 0.21958 | 0.046 | 1.7983 | 67 |
| ConA | BDGlu | 48 | MO000038315 | LPS:lbp:CD14:TLR4:MD-2:TIRAP | 0.22086 | 0.023 | 1.7965 | 67 |
| ConA | BDGlu | 48 | MO000098006 | Nod2(m) | 0.22689 | 0.033 | 1.7482 | 68 |
| ConA | BDGlu | 48 | MO000165178 | ERK2(m){pY185} | 0.27551 | 0.044 | 1.4407 | 69 |
| ConA | BDGlu | 48 | MO000109727 | ERK2(m){pT183} | 0.27551 | 0.045 | 1.4285 | 74 |
| ConA | BDGlu | 48 | MO000482267 | ERK2(m){pT} | 0.27551 | 0.045 | 1.4346 | 74 |
| ConA | BDGlu | 48 | MO000166501 | CpG B | 0.23027 | 0.042 | 1.5265 | 78 |
| ConA | BDGlu | 48 | MO000032843 | CrkL(m) | 0.22269 | 0.038 | 1.5376 | 80 |
| ConA | BDGlu | 48 | MO000155959 | IL-17RA(m) | 0.20103 | 0.046 | 1.5996 | 80 |
| ConA | BDGlu | 96 | MO000165399 | IKK-i(m){p} | 0.32368 | 0.015 | 2.6045 | 23 |
| ConA | BDGlu | 96 | MO000021888 | FOXO1A(m) | 0.35655 | 0.007 | 2.2616 | 25 |
| ConA | BDGlu | 96 | MO000128620 | EAC(m) | 0.43332 | 0.02 | 1.8538 | 25 |
| ConA | BDGlu | 96 | MO000035094 | cGKI(m) | 0.38927 | 0.011 | 1.8989 | 26 |
| ConA | BDGlu | 96 | MO000120165 | IRF-3(m) | 0.29083 | 0.011 | 2.9128 | 26 |
| ConA | BDGlu | 96 | MO000019336 | TBK1(m) | 0.41511 | 0.038 | 1.7528 | 27 |
| ConA | BDGlu | 96 | MO000083443 | FOXO1A(m) | 0.26649 | 0.006 | 3.8201 | 30 |
| ConA | BDGlu | 96 | MO000032972 | IRF-3(m) | 0.27419 | 0.005 | 2.6948 | 31 |
| ConA | BDGlu | 96 | MO000120169 | Ubc13(m) | 0.50308 | 0.016 | 1.6249 | 31 |
| ConA | BDGlu | 96 | MO000082357 | ErbB1(m) | 0.46736 | 0.033 | 1.6258 | 32 |
| ConA | BDGlu | 96 | MO000086680 | TNF-alpha(m) | 0.37473 | 0.028 | 1.7158 | 32 |
| ConA | BDGlu | 96 | MO000088390 | IRF-8(m) | 0.29046 | 0.014 | 2.5898 | 32 |
| ConA | BDGlu | 96 | MO000106371 | CaMKII(m) | 0.34261 | 0.031 | 2.0275 | 32 |
| ConA | BDGlu | 96 | MO000120045 | UbcH7(m) | 0.43999 | 0.033 | 1.6118 | 35 |
| ConA | BDGlu | 96 | MO000007186 | IRF-8(m) | 0.2902 | 0.015 | 2.1543 | 39 |
| ConA | BDGlu | 96 | MO000200602 | march5(m) | 0.35516 | 0.03 | 1.6631 | 39 |
| ConA | BDGlu | 96 | MO000336674 | SGK-1(m){pT256} | 0.31542 | 0.039 | 1.6641 | 43 |
| ConA | BDGlu | 96 | MO000034664 | c-Cbl(m) | 0.39757 | 0.04 | 1.5064 | 44 |
| ConA | BDGlu | 96 | MO000033754 | PAK1(m) | 0.53021 | 0.034 | 1.3171 | 45 |
| ConA | BDGlu | 96 | MO000081629 | E1(m) | 0.58182 | 0.02 | 1.2622 | 45 |
| ConA | BDGlu | 96 | MO000036158 | TBK1(m):TANK(m) | 0.20818 | 0.016 | 3.9367 | 46 |
| ConA | BDGlu | 96 | MO000200601 | march5(m) | 0.35514 | 0.041 | 1.5709 | 46 |
| ConA | BDGlu | 96 | MO000114796 | AMPKalpha(h){pT} | 0.22377 | 0.006 | 2.6022 | 47 |
| ConA | BDGlu | 96 | MO000032827 | PKCepsilon(m) | 0.5329 | 0.047 | 1.0837 | 48 |
| ConA | BDGlu | 96 | MO000149925 | Ro52(m) | 0.2164 | 0.023 | 2.7372 | 48 |
| ConA | BDGlu | 96 | MO000034986 | LMW-PTP(m) | 0.48047 | 0.048 | 1.2452 | 50 |
| ConA | BDGlu | 96 | MO000067266 | cGKII(m) | 0.22375 | 0.006 | 2.5052 | 50 |
| ConA | BDGlu | 96 | MO000188322 | E1{ub(1)}:Ubc13:Mms2 | 0.26155 | 0.034 | 1.7258 | 51 |
| ConA | BDGlu | 96 | MO000279508 | Fcor(m) | 0.22369 | 0.012 | 2.3855 | 52 |
| ConA | BDGlu | 96 | MO000149650 | TBK1(m) | 0.25574 | 0.04 | 1.8959 | 53 |
| ConA | BDGlu | 96 | MO000120168 | Ubc13(m) | 0.36597 | 0.04 | 1.3214 | 54 |
| ConA | BDGlu | 96 | MO000131412 | E1:Ubc5C{ub(1)} | 0.22575 | 0.015 | 2.0578 | 54 |
| ConA | BDGlu | 96 | MO000084535 | MAFbx(m) | 0.22326 | 0.018 | 2.3475 | 55 |
| ConA | BDGlu | 96 | MO000120961 | E1:Ubc7{ub(1)} | 0.26155 | 0.034 | 1.6911 | 55 |
| ConA | BDGlu | 96 | MO000120050 | UBE2G2(m) | 0.2613 | 0.034 | 1.6919 | 57 |
| ConA | BDGlu | 96 | MO000253996 | TBK1(m){pS172} | 0.20795 | 0.013 | 2.3667 | 57 |
| ConA | BDGlu | 96 | MO000083333 | Fyn(m){pY} | 0.31142 | 0.037 | 1.4725 | 59 |
| ConA | BDGlu | 96 | MO000113198 | T6BP(m) | 0.34442 | 0.04 | 1.2652 | 60 |
| ConA | BDGlu | 96 | MO000119264 | E1:UbcH7{ub(1)} | 0.26154 | 0.034 | 1.6372 | 60 |
| ConA | BDGlu | 96 | MO000085462 | c-Cbl(m) | 0.29912 | 0.046 | 1.4125 | 61 |
| ConA | BDGlu | 96 | MO000161841 | ube2h(m) | 0.31449 | 0.047 | 1.3292 | 61 |
| ConA | BDGlu | 96 | MO000160215 | E1:Ubc5A{ub(1)} | 0.26155 | 0.034 | 1.5649 | 62 |
| ConA | BDGlu | 96 | MO000200433 | RPTPkappa(m) | 0.26827 | 0.045 | 1.5133 | 62 |
| ConA | BDGlu | 96 | MO000032775 | p75NTR(m) | 0.2949 | 0.049 | 1.3858 | 63 |
| ConA | BDGlu | 96 | MO000170572 | IKK-beta(m){pS} | 0.24086 | 0.032 | 1.5881 | 67 |
| ConA | BDGlu | 96 | MO000187670 | E1{ub(1)}:Ubc13:Uev1 | 0.26155 | 0.034 | 1.4978 | 69 |
| ConA | BDGlu | 96 | MO000165629 | IKK-beta(m){pS177}{pS181} | 0.24035 | 0.043 | 1.5633 | 71 |
| ConA | BDGlu | 96 | MO000285932 | Cezanne(m) | 0.23877 | 0.035 | 1.497 | 76 |
| ConA | BDGlu | 96 | MO000089823 | cIAP-2(m) | 0.42776 | 0.011 | 3.1704 | 27 |
| ConA | BDGlu | 96 | MO000059714 | ErbB3(m) | 0.41721 | 0.003 | 2.7394 | 52 |
| ConA | BDGlu | 96 | MO000120045 | UbcH7(m) | 0.45388 | 0.001 | 2.4375 | 60 |
| ConA | BDGlu | 96 | MO000149650 | TBK1(m) | 0.39462 | 0.004 | 2.696 | 61 |
| ConA | BDGlu | 96 | MO000059735 | ErbB4(m) | 0.41713 | 0.013 | 2.5649 | 65 |
| ConA | BDGlu | 96 | MO000114529 | Src(m){pY} | 0.44894 | 0.003 | 2.2628 | 72 |
| ConA | BDGlu | 96 | MO000044385 | parkin(m) | 0.35864 | 0.003 | 2.7194 | 74 |
| ConA | BDGlu | 96 | MO000103567 | E2-C(m) | 0.45533 | 0.001 | 2.1824 | 78 |
| ConA | BDGlu | 96 | MO000017705 | FcepsilonRI | 0.33394 | 0 | 2.7582 | 80 |
| ConA | BDGlu | 96 | MO000041182 | TRIF(m) | 0.41308 | 0.005 | 2.34 | 80 |
| ConA | BDGlu | 96 | MO000253996 | TBK1(m){pS172} | 0.31542 | 0.004 | 3.0826 | 80 |
| ConA | BDGlu | 96 | MO000120015 | TLR3(m) | 0.30385 | 0.002 | 3.6139 | 81 |
| ConA | BDGlu | 96 | MO000022202 | IRAK-1(m) | 0.36335 | 0.01 | 2.5433 | 85 |
| ConA | BDGlu | 96 | MO000022316 | PKCiota(m) | 0.42173 | 0.002 | 2.1857 | 87 |
| ConA | BDGlu | 96 | MO000062597 | FCRG(m) | 0.31966 | 0 | 2.8408 | 88 |
| ConA | BDGlu | 96 | MO000020140 | TCR(m) | 0.30111 | 0 | 3.0995 | 89 |
| ConA | BDGlu | 96 | MO000120165 | IRF-3(m) | 0.2975 | 0.007 | 3.3576 | 90 |
| ConA | BDGlu | 96 | MO000032972 | IRF-3(m) | 0.29933 | 0.013 | 3.1598 | 91 |
| ConA | BDGlu | 96 | MO000082402 | MEKK1-xbb2(m) | 0.36464 | 0.001 | 2.341 | 91 |
| ConA | BDGlu | 96 | MO000165399 | IKK-i(m){p} | 0.30596 | 0.006 | 2.905 | 91 |
| ConA | BDGlu | 96 | MO000019336 | TBK1(m) | 0.561 | 0.002 | 2.0455 | 93 |
| ConA | BDGlu | 96 | MO000166537 | FcgammaRIII(m) | 0.31236 | 0 | 2.8697 | 93 |
| ConA | BDGlu | 96 | MO000075867 | MEKK1(m) | 0.36464 | 0.001 | 2.3273 | 95 |
| ConA | BDGlu | 96 | MO000017821 | Igalpha:Igbeta | 0.3129 | 0 | 2.7323 | 99 |
| ConA | BDGlu | 96 | MO000018208 | FcgammaRI | 0.31306 | 0 | 2.7246 | 99 |
| ConA | BDGlu | 96 | MO000156307 | FcepsilonRI beta(m) | 0.3313 | 0.001 | 2.5159 | 100 |
| ConA | BDGlu | 96 | MO000057591 | PLCgamma2(m) | 0.29449 | 0 | 2.9082 | 104 |
| ConA | BDGlu | 96 | MO000043163 | Caspase-8(m) | 0.32311 | 0.008 | 2.5505 | 106 |
| ConA | BDGlu | 96 | MO000037455 | Cas(m) | 0.32921 | 0.005 | 2.362 | 108 |
| ConA | BDGlu | 96 | MO000037747 | LynA(m) | 0.3936 | 0.001 | 2.1298 | 111 |
| ConA | BDGlu | 96 | MO000037746 | LynB(m) | 0.3936 | 0.001 | 2.1298 | 113 |
| ConA | BDGlu | 96 | MO000082357 | ErbB1(m) | 0.44712 | 0.013 | 1.9462 | 116 |
| ConA | BDGlu | 96 | MO000034766 | HEF1(m) | 0.32996 | 0.004 | 2.2252 | 118 |
| ConA | BDGlu | 96 | MO000086689 | NRG2(m) | 0.30042 | 0.004 | 2.6098 | 119 |
| ConA | BDGlu | 96 | MO000120961 | E1:Ubc7{ub(1)} | 0.28131 | 0.001 | 2.8812 | 119 |
| ConA | BDGlu | 96 | MO000160215 | E1:Ubc5A{ub(1)} | 0.28131 | 0.001 | 2.8811 | 119 |
| ConA | BDGlu | 96 | MO000188322 | E1{ub(1)}:Ubc13:Mms2 | 0.28131 | 0.001 | 2.8812 | 119 |
| ConA | BDGlu | 96 | MO000119264 | E1:UbcH7{ub(1)} | 0.28131 | 0.001 | 2.8826 | 120 |
| ConA | BDGlu | 96 | MO000025653 | IRF-4(m) | 0.27771 | 0.012 | 3.0495 | 121 |
| ConA | BDGlu | 96 | MO000038316 | LPS:lbp:CD14:TLR4:MD-2:TIRAP:IRAK-2 | 0.36818 | 0.004 | 2.0886 | 123 |
| ConA | BDGlu | 96 | MO000041437 | dsRNA:TLR3:TRIF | 0.36817 | 0.004 | 2.0906 | 123 |
| ConA | BDGlu | 96 | MO000187670 | E1{ub(1)}:Ubc13:Uev1 | 0.28131 | 0.001 | 2.88 | 123 |
| ConA | BDGlu | 96 | MO000044588 | parkin:Cul-1:Fbw7 | 0.26603 | 0 | 3.0848 | 126 |
| ConA | BDGlu | 96 | MO000086151 | traf6-isoform1(m) | 0.34979 | 0.016 | 2.1459 | 126 |
| ConA | BDGlu | 96 | MO000036158 | TBK1(m):TANK(m) | 0.25679 | 0.009 | 4.0453 | 127 |
| ConA | BDGlu | 96 | MO000120050 | UBE2G2(m) | 0.28106 | 0.001 | 2.8459 | 127 |
| ConA | BDGlu | 96 | MO000034664 | c-Cbl(m) | 0.3917 | 0.007 | 2.019 | 128 |
| ConA | BDGlu | 96 | MO000089319 | Caspase(m) | 0.38255 | 0.013 | 2.0312 | 129 |
| ConA | BDGlu | 96 | MO000120044 | UbcH7(m) | 0.28383 | 0.007 | 2.6809 | 132 |
| ConA | BDGlu | 96 | MO000130073 | PKCiota(m) | 0.27217 | 0.001 | 2.8217 | 133 |
| ConA | BDGlu | 96 | MO000019402 | traf6(m) | 0.50529 | 0.029 | 1.7126 | 136 |
| ConA | BDGlu | 96 | MO000081647 | IFNgamma(m) | 0.31899 | 0.028 | 2.1802 | 136 |
| ConA | BDGlu | 96 | MO000092386 | ErbB2(m) | 0.33855 | 0.016 | 2.1171 | 136 |
| ConA | BDGlu | 96 | MO000131412 | E1:Ubc5C{ub(1)} | 0.25208 | 0 | 3.2454 | 137 |
| ConA | BDGlu | 96 | MO000007513 | IFNgamma(m) | 0.28847 | 0.008 | 2.453 | 139 |
| ConA | BDGlu | 96 | MO000019403 | SITPEC(m) | 0.29636 | 0.002 | 2.338 | 140 |
| ConA | BDGlu | 96 | MO000020142 | BCR(m) | 0.28792 | 0.001 | 2.4911 | 140 |
| ConA | BDGlu | 96 | MO000020192 | SHP-1(m) | 0.52113 | 0.005 | 1.6842 | 141 |
| ConA | BDGlu | 96 | MO000007687 | IRF-1(m) | 0.24648 | 0.02 | 3.3966 | 142 |
| ConA | BDGlu | 96 | MO000032784 | PLCgamma1(m) | 0.28099 | 0.002 | 2.6568 | 142 |
| ConA | BDGlu | 96 | MO000019483 | map4k4(m) | 0.29626 | 0.002 | 2.3161 | 144 |
| ConA | BDGlu | 96 | MO000020145 | TRAF2(m) | 0.31744 | 0.008 | 2.1436 | 144 |
| ConA | BDGlu | 96 | MO000085462 | c-Cbl(m) | 0.29549 | 0.006 | 2.2742 | 147 |
| ConA | BDGlu | 96 | MO000006539 | Hck(m) | 0.45379 | 0.01 | 1.6813 | 149 |
| ConA | BDGlu | 96 | MO000200602 | march5(m) | 0.32447 | 0.013 | 2.0853 | 149 |
| ConA | BDGlu | 96 | MO000019485 | IFNalpha, IFNbeta:IFNAR1{pY}:Tyk2{pY}:IFNAR2c{pY}:Jak1{pY} | 0.29949 | 0.015 | 2.1807 | 150 |
| ConA | BDGlu | 96 | MO000008302 | Syk(m) | 0.52868 | 0.003 | 1.6492 | 151 |
| ConA | BDGlu | 96 | MO000079158 | Syk(m) | 0.32792 | 0.02 | 2.035 | 152 |
| ConA | BDGlu | 96 | MO000149925 | Ro52(m) | 0.23357 | 0.006 | 3.2288 | 155 |
| ConA | BDGlu | 96 | MO000022056 | Jak1(m) | 0.48776 | 0.019 | 1.6462 | 157 |
| ConA | BDGlu | 96 | MO000084046 | CD19(m) | 0.32823 | 0.015 | 1.9898 | 157 |
| ConA | BDGlu | 96 | MO000234738 | Irak2(m) | 0.28633 | 0.012 | 2.2627 | 157 |
| ConA | BDGlu | 96 | MO000033240 | EpoR(m) | 0.37675 | 0.016 | 1.7852 | 159 |
| ConA | BDGlu | 96 | MO000085966 | IRF-4-isoform1(m) | 0.22356 | 0.014 | 5.4364 | 159 |
| ConA | BDGlu | 96 | MO000021349 | RPTPalpha(m) | 0.41363 | 0.019 | 1.6881 | 160 |
| ConA | BDGlu | 96 | MO000059699 | ErbB2(m) | 0.53607 | 0.012 | 1.5934 | 161 |
| ConA | BDGlu | 96 | MO000088546 | IRF-7(m) | 0.22293 | 0.015 | 4.2353 | 162 |
| ConA | BDGlu | 96 | MO000236690 | Mad2l1bp(m) | 0.25542 | 0.004 | 2.6654 | 163 |
| ConA | BDGlu | 96 | MO000087375 | PIAS3-isoform2(m) | 0.35153 | 0.025 | 1.8204 | 165 |
| ConA | BDGlu | 96 | MO000200601 | march5(m) | 0.32445 | 0.028 | 1.9387 | 165 |
| ConA | BDGlu | 96 | MO000020892 | Par3(m) | 0.2627 | 0.001 | 2.4444 | 166 |
| ConA | BDGlu | 96 | MO000081629 | E1(m) | 0.54939 | 0.002 | 1.555 | 167 |
| ConA | BDGlu | 96 | MO000079029 | Jak1(m) | 0.28982 | 0.016 | 2.1415 | 169 |
| ConA | BDGlu | 96 | MO000032010 | H-Ras(m) | 0.38455 | 0.023 | 1.6814 | 170 |
| ConA | BDGlu | 96 | MO000007186 | IRF-8(m) | 0.23775 | 0.013 | 2.756 | 172 |
| ConA | BDGlu | 96 | MO000032890 | PTP-PEST(m) | 0.36428 | 0.017 | 1.6998 | 172 |
| ConA | BDGlu | 96 | MO000042840 | ptpn21(m) | 0.35724 | 0.011 | 1.7261 | 172 |
| ConA | BDGlu | 96 | MO000039080 | IRAK-4(m) | 0.25122 | 0.002 | 2.62 | 173 |
| ConA | BDGlu | 96 | MO000058103 | CD45(m) | 0.42801 | 0.045 | 1.6012 | 173 |
| ConA | BDGlu | 96 | MO000119664 | IRAK-4(m) | 0.25112 | 0.002 | 2.6471 | 173 |
| ConA | BDGlu | 96 | MO000008801 | Lyn(m) | 0.57167 | 0.005 | 1.4006 | 175 |
| ConA | BDGlu | 96 | MO000038241 | itch(m) | 0.44322 | 0.05 | 1.5663 | 176 |
| ConA | BDGlu | 96 | MO000041137 | TRAM(m) | 0.24178 | 0.002 | 2.669 | 176 |
| ConA | BDGlu | 96 | MO000057306 | Src(m) | 0.35509 | 0.02 | 1.7118 | 177 |
| ConA | BDGlu | 96 | MO000086968 | HPK1(m) | 0.296 | 0.006 | 2.0653 | 177 |
| ConA | BDGlu | 96 | MO000088390 | IRF-8(m) | 0.23021 | 0.021 | 2.8074 | 177 |
| ConA | BDGlu | 96 | MO000150828 | PKD1(m) | 0.31208 | 0.02 | 1.931 | 177 |
| ConA | BDGlu | 96 | MO000180126 | PP2Cbeta(m) | 0.43906 | 0.012 | 1.5654 | 178 |
| ConA | BDGlu | 96 | MO000245279 | Usp25(m) | 0.31241 | 0.017 | 1.9207 | 178 |
| ConA | BDGlu | 96 | MO000013004 | Src(m) | 0.55621 | 0.048 | 1.2136 | 179 |
| ConA | BDGlu | 96 | MO000146524 | pkmyt1(m) | 0.41868 | 0.013 | 1.5926 | 179 |
| ConA | BDGlu | 96 | MO000127914 | Cdc25C(m) | 0.41861 | 0.015 | 1.5861 | 181 |
| ConA | BDGlu | 96 | MO000009087 | MEKK1(m) | 0.50487 | 0.028 | 1.4035 | 182 |
| ConA | BDGlu | 96 | MO000021719 | A20(m) | 0.32331 | 0.037 | 1.8088 | 182 |
| ConA | BDGlu | 96 | MO000059191 | AKT(m){p} | 0.35701 | 0.019 | 1.6791 | 183 |
| ConA | BDGlu | 96 | MO000033795 | Tyk2(m) | 0.39544 | 0.048 | 1.5972 | 184 |
| ConA | BDGlu | 96 | MO000117511 | TC-PTPa(m) | 0.43581 | 0.034 | 1.5209 | 186 |
| ConA | BDGlu | 96 | MO000059453 | Jak2(m){pY} | 0.28678 | 0.032 | 2.0569 | 187 |
| ConA | BDGlu | 96 | MO000019393 | IL-1RAcP(m) | 0.2302 | 0.003 | 2.5216 | 197 |
| ConA | BDGlu | 96 | MO000103566 | E2-C(m) | 0.25805 | 0.012 | 2.1499 | 198 |
| ConA | BDGlu | 96 | MO000019839 | Cdc34(m) | 0.35155 | 0.046 | 1.6294 | 199 |
| ConA | BDGlu | 96 | MO000187933 | Wee1(m) | 0.41808 | 0.045 | 1.3826 | 199 |
| ConA | BDGlu | 96 | MO000200433 | RPTPkappa(m) | 0.25826 | 0.012 | 2.1353 | 201 |
| ConA | BDGlu | 96 | MO000022224 | dsRNA | 0.24582 | 0.009 | 2.2409 | 202 |
| ConA | BDGlu | 96 | MO000032775 | p75NTR(m) | 0.27783 | 0.022 | 2.0252 | 202 |
| ConA | BDGlu | 96 | MO000085229 | cyclinB1(m) | 0.32531 | 0.029 | 1.6669 | 202 |
| ConA | BDGlu | 96 | MO000098007 | Nod2(m) | 0.30579 | 0.031 | 1.7723 | 202 |
| ConA | BDGlu | 96 | MO000121660 | alpha IIb-integrin(m):beta3-integrin(m) | 0.2844 | 0.015 | 1.9239 | 204 |
| ConA | BDGlu | 96 | MO000021312 | PP2Cbeta1(m) | 0.38486 | 0.025 | 1.4218 | 206 |
| ConA | BDGlu | 96 | MO000120168 | Ubc13(m) | 0.33875 | 0.027 | 1.615 | 206 |
| ConA | BDGlu | 96 | MO000042988 | Nrdp1(m) | 0.22663 | 0.003 | 2.4316 | 207 |
| ConA | BDGlu | 96 | MO000046028 | NF-YA-isoform1(m) | 0.28811 | 0.023 | 1.8436 | 207 |
| ConA | BDGlu | 96 | MO000054385 | Ret(m) | 0.25816 | 0.033 | 2.1095 | 207 |
| ConA | BDGlu | 96 | MO000122582 | IKK-i(m) | 0.30643 | 0.035 | 1.6993 | 207 |
| ConA | BDGlu | 96 | MO000030839 | Wee1(m) | 0.28773 | 0.016 | 1.8453 | 208 |
| ConA | BDGlu | 96 | MO000038315 | LPS:lbp:CD14:TLR4:MD-2:TIRAP | 0.22326 | 0.002 | 2.3212 | 216 |
| ConA | BDGlu | 96 | MO000130220 | PEP(m) | 0.34259 | 0.033 | 1.5533 | 216 |
| ConA | BDGlu | 96 | MO000239966 | Ptpn23(m) | 0.35641 | 0.038 | 1.5038 | 216 |
| ConA | BDGlu | 96 | MO000095704 | TRIF(m) | 0.26419 | 0.015 | 1.9808 | 217 |
| ConA | BDGlu | 96 | MO000035461 | Syk(m){pY} | 0.20082 | 0.004 | 2.56 | 218 |
| ConA | BDGlu | 96 | MO000036067 | TRAF3(m) | 0.26746 | 0.02 | 1.9297 | 218 |
| ConA | BDGlu | 96 | MO000149349 | RNF126(m) | 0.25827 | 0.014 | 2.0153 | 218 |
| ConA | BDGlu | 96 | MO000240859 | Rnf115(m) | 0.25827 | 0.014 | 2.0149 | 218 |
| ConA | BDGlu | 96 | MO000019329 | TANK(m) | 0.22531 | 0.01 | 2.217 | 221 |
| ConA | BDGlu | 96 | MO000170572 | IKK-beta(m){pS} | 0.23257 | 0.01 | 2.1299 | 229 |
| ConA | BDGlu | 96 | MO000059659 | IRAK-1(m){p} | 0.29927 | 0.046 | 1.6298 | 233 |
| ConA | BDGlu | 96 | MO000233616 | Gprc5b(m) | 0.24028 | 0.005 | 2.0571 | 233 |
| ConA | BDGlu | 96 | MO000081385 | TAK1(m) | 0.2711 | 0.017 | 1.7794 | 234 |
| ConA | BDGlu | 96 | MO000165056 | cIAP-1(m) | 0.30816 | 0.05 | 1.5687 | 234 |
| ConA | BDGlu | 96 | MO000032785 | Yes(m) | 0.2671 | 0.032 | 1.8051 | 235 |
| ConA | BDGlu | 96 | MO000084051 | Lyn(m){pY397} | 0.20773 | 0.003 | 2.1906 | 235 |
| ConA | BDGlu | 96 | MO000088384 | Pkm2(m) | 0.21682 | 0.025 | 2.1757 | 235 |
| ConA | BDGlu | 96 | MO000107884 | Cbl-b(m) | 0.25641 | 0.031 | 1.9137 | 235 |
| ConA | BDGlu | 96 | MO000042687 | CIN85(m) | 0.22236 | 0.011 | 2.1468 | 237 |
| ConA | BDGlu | 96 | MO000084053 | Lyn(m){pY397}{pY508} | 0.20773 | 0.003 | 2.1927 | 237 |
| ConA | BDGlu | 96 | MO000121034 | CIKS(m) | 0.29915 | 0.048 | 1.6163 | 237 |
| ConA | BDGlu | 96 | MO000126162 | Cd72(m) | 0.25583 | 0.024 | 1.9115 | 237 |
| ConA | BDGlu | 96 | MO000165629 | IKK-beta(m){pS177}{pS181} | 0.23211 | 0.014 | 2.0866 | 237 |
| ConA | BDGlu | 96 | MO000165441 | Syk(m){p} | 0.20194 | 0.003 | 2.2087 | 239 |
| ConA | BDGlu | 96 | MO000197545 | Lyn(m){pY508} | 0.20773 | 0.003 | 2.1637 | 242 |
| ConA | BDGlu | 96 | MO000105496 | Lyn(m){pY} | 0.20773 | 0.003 | 2.1589 | 244 |
| ConA | BDGlu | 96 | MO000017252 | IL-10R | 0.25182 | 0.03 | 1.8108 | 249 |
| ConA | BDGlu | 96 | MO000019522 | IL-22R1:IL-10R2 | 0.25182 | 0.03 | 1.8108 | 251 |
| ConA | BDGlu | 96 | MO000037966 | Gab-1(m) | 0.2686 | 0.042 | 1.668 | 253 |
| ConA | BDGlu | 96 | MO000044233 | Jak2(m){pY1007} | 0.23797 | 0.034 | 1.9058 | 255 |
| ConA | BDGlu | 96 | MO000037506 | gamma-secretase(m) | 0.24726 | 0.039 | 1.808 | 257 |
| ConA | BDGlu | 96 | MO000083014 | Hck-isoform1(m) | 0.25362 | 0.037 | 1.7287 | 257 |
| ConA | BDGlu | 96 | MO000035100 | RanGAP1(m) | 0.26129 | 0.039 | 1.6436 | 268 |
| ConA | BDGlu | 96 | MO000028143 | FOXP3(m) | 0.20826 | 0.018 | 1.9806 | 269 |
| ConA | BDGlu | 96 | MO000151911 | IKK-alpha(m){p} | 0.2545 | 0.041 | 1.6715 | 269 |
| ConA | BDGlu | 96 | MO000016669 | IFNRI | 0.2513 | 0.044 | 1.678 | 273 |
| ConA | BDGlu | 96 | MO000001761 | Blk(m) | 0.2312 | 0.032 | 1.7412 | 277 |
| ConA | BDGlu | 96 | MO000057490 | InsR(m){pY} | 0.2213 | 0.014 | 1.8302 | 278 |
| ConA | BDGlu | 96 | MO000336179 | TAK1(m){ub} | 0.27051 | 0.038 | 1.5417 | 278 |
| ConA | BDGlu | 96 | MO000084602 | IKK-gamma(m) | 0.20678 | 0.022 | 1.914 | 282 |
| ConA | BDGlu | 96 | MO000095010 | pawr(m) | 0.26557 | 0.048 | 1.5437 | 282 |
| ConA | BDGlu | 96 | MO000098006 | Nod2(m) | 0.20747 | 0.015 | 1.878 | 285 |
| ConA | BDGlu | 96 | MO000032886 | C3G(m) | 0.21564 | 0.027 | 1.801 | 288 |
| ConA | BDGlu | 96 | MO000119721 | tab3(m) | 0.24628 | 0.017 | 1.625 | 291 |
| ConA | BDGlu | 96 | MO000043647 | Tome-1(m) | 0.23502 | 0.033 | 1.6473 | 293 |
| ConA | BDGlu | 96 | MO000032836 | IFNAR2(m) | 0.22927 | 0.031 | 1.6655 | 297 |
| ConA | BDGlu | 96 | MO000023660 | IL-1RI(m) | 0.21305 | 0.026 | 1.6956 | 300 |
| ConA | BDGlu | 96 | MO000032852 | OSMRbeta(m) | 0.22925 | 0.031 | 1.664 | 300 |
| ConA | BDGlu | 96 | MO000041135 | tab2(m) | 0.24624 | 0.017 | 1.5246 | 308 |
| ConA | BDGlu | 96 | MO000087723 | Cdk1(m):cyclinB1(m) | 0.23446 | 0.05 | 1.4981 | 318 |
| ConA | BDGlu | 96 | MO000089140 | gp130(m) | 0.22905 | 0.048 | 1.5647 | 319 |
| ConA | BDGlu | 96 | MO000032843 | CrkL(m) | 0.20124 | 0.03 | 1.6655 | 320 |
| ConA | BDGlu | 96 | MO000036021 | EDAR(m) | 0.21593 | 0.041 | 1.4967 | 337 |
| ConA + Poly I:C | BDGlu | 48 | MO000089823 | cIAP-2(m) | 0.40675 | 0.026 | 3.3675 | 42 |
| ConA + Poly I:C | BDGlu | 48 | MO000019336 | TBK1(m) | 0.53761 | 0.001 | 2.5336 | 51 |
| ConA + Poly I:C | BDGlu | 48 | MO000120045 | UbcH7(m) | 0.4625 | 0.006 | 2.6089 | 51 |
| ConA + Poly I:C | BDGlu | 48 | MO000089319 | Caspase(m) | 0.45605 | 0.035 | 2.5807 | 58 |
| ConA + Poly I:C | BDGlu | 48 | MO000041182 | TRIF(m) | 0.39693 | 0.001 | 2.85 | 61 |
| ConA + Poly I:C | BDGlu | 48 | MO000007348 | IL-2Rbeta(m) | 0.43949 | 0 | 2.4571 | 69 |
| ConA + Poly I:C | BDGlu | 48 | MO000232618 | beta-TrCP1-isoform1(m) | 0.3487 | 0.033 | 3.1094 | 69 |
| ConA + Poly I:C | BDGlu | 48 | MO000079029 | Jak1(m) | 0.35269 | 0.003 | 2.904 | 76 |
| ConA + Poly I:C | BDGlu | 48 | MO000022316 | PKCiota(m) | 0.42506 | 0.002 | 2.4194 | 77 |
| ConA + Poly I:C | BDGlu | 48 | MO000234738 | Irak2(m) | 0.33362 | 0.004 | 2.9733 | 82 |
| ConA + Poly I:C | BDGlu | 48 | MO000032010 | H-Ras(m) | 0.44113 | 0.009 | 2.2854 | 84 |
| ConA + Poly I:C | BDGlu | 48 | MO000032775 | p75NTR(m) | 0.33013 | 0.004 | 2.8599 | 92 |
| ConA + Poly I:C | BDGlu | 48 | MO000149650 | TBK1(m) | 0.32299 | 0.005 | 2.8737 | 92 |
| ConA + Poly I:C | BDGlu | 48 | MO000013127 | STAT5A(m) | 0.29616 | 0.019 | 3.045 | 93 |
| ConA + Poly I:C | BDGlu | 48 | MO000121772 | A20(m) | 0.40981 | 0.007 | 2.2316 | 94 |
| ConA + Poly I:C | BDGlu | 48 | MO000117511 | TC-PTPa(m) | 0.54088 | 0.004 | 2.0932 | 96 |
| ConA + Poly I:C | BDGlu | 48 | MO000059714 | ErbB3(m) | 0.38817 | 0.019 | 2.417 | 98 |
| ConA + Poly I:C | BDGlu | 48 | MO000013134 | STAT5B(m) | 0.29316 | 0.015 | 2.986 | 99 |
| ConA + Poly I:C | BDGlu | 48 | MO000120015 | TLR3(m) | 0.28365 | 0 | 3.6927 | 99 |
| ConA + Poly I:C | BDGlu | 48 | MO000017705 | FcepsilonRI | 0.3119 | 0.001 | 2.8127 | 100 |
| ConA + Poly I:C | BDGlu | 48 | MO000200602 | march5(m) | 0.38801 | 0.006 | 2.397 | 100 |
| ConA + Poly I:C | BDGlu | 48 | MO000128620 | EAC(m) | 0.43403 | 0.005 | 2.1819 | 101 |
| ConA + Poly I:C | BDGlu | 48 | MO000098007 | Nod2(m) | 0.38062 | 0.006 | 2.365 | 104 |
| ConA + Poly I:C | BDGlu | 48 | MO000022202 | IRAK-1(m) | 0.39108 | 0.024 | 2.3121 | 105 |
| ConA + Poly I:C | BDGlu | 48 | MO000038316 | LPS:lbp:CD14:TLR4:MD-2:TIRAP:IRAK-2 | 0.40899 | 0.004 | 2.2087 | 105 |
| ConA + Poly I:C | BDGlu | 48 | MO000041437 | dsRNA:TLR3:TRIF | 0.40898 | 0.004 | 2.2088 | 105 |
| ConA + Poly I:C | BDGlu | 48 | MO000059659 | IRAK-1(m){p} | 0.37802 | 0.008 | 2.3375 | 106 |
| ConA + Poly I:C | BDGlu | 48 | MO000019485 | IFNalpha, IFNbeta:IFNAR1{pY}:Tyk2{pY}:IFNAR2c{pY}:Jak1{pY} | 0.33077 | 0.008 | 2.6048 | 109 |
| ConA + Poly I:C | BDGlu | 48 | MO000166537 | FcgammaRIII(m) | 0.29708 | 0.003 | 2.7376 | 109 |
| ConA + Poly I:C | BDGlu | 48 | MO000016606 | IKK-gamma(m) | 0.36921 | 0.021 | 2.3342 | 110 |
| ConA + Poly I:C | BDGlu | 48 | MO000121034 | CIKS(m) | 0.37794 | 0.008 | 2.3247 | 111 |
| ConA + Poly I:C | BDGlu | 48 | MO000245279 | Usp25(m) | 0.38285 | 0.008 | 2.3087 | 111 |
| ConA + Poly I:C | BDGlu | 48 | MO000021349 | RPTPalpha(m) | 0.451 | 0.013 | 2.0772 | 112 |
| ConA + Poly I:C | BDGlu | 48 | MO000086151 | traf6-isoform1(m) | 0.3958 | 0.024 | 2.216 | 112 |
| ConA + Poly I:C | BDGlu | 48 | MO000017162 | c-Kit(m) | 0.45246 | 0.02 | 2.0406 | 117 |
| ConA + Poly I:C | BDGlu | 48 | MO000022056 | Jak1(m) | 0.56129 | 0.008 | 1.9448 | 117 |
| ConA + Poly I:C | BDGlu | 48 | MO000032271 | SOCS-1(m) | 0.4336 | 0.004 | 2.0951 | 117 |
| ConA + Poly I:C | BDGlu | 48 | MO000021719 | A20(m) | 0.40058 | 0.011 | 2.1756 | 118 |
| ConA + Poly I:C | BDGlu | 48 | MO000008302 | Syk(m) | 0.53854 | 0.016 | 1.9345 | 120 |
| ConA + Poly I:C | BDGlu | 48 | MO000017821 | Igalpha:Igbeta | 0.29169 | 0.001 | 2.6646 | 121 |
| ConA + Poly I:C | BDGlu | 48 | MO000044588 | parkin:Cul-1:Fbw7 | 0.26112 | 0.001 | 3.0644 | 121 |
| ConA + Poly I:C | BDGlu | 48 | MO000103567 | E2-C(m) | 0.45135 | 0.016 | 2.0267 | 121 |
| ConA + Poly I:C | BDGlu | 48 | MO000200601 | march5(m) | 0.38799 | 0.012 | 2.2056 | 123 |
| ConA + Poly I:C | BDGlu | 48 | MO000156307 | FcepsilonRI beta(m) | 0.33269 | 0.017 | 2.3281 | 124 |
| ConA + Poly I:C | BDGlu | 48 | MO000024810 | ER-alpha(m) | 0.2708 | 0.042 | 2.8117 | 125 |
| ConA + Poly I:C | BDGlu | 48 | MO000120044 | UbcH7(m) | 0.30787 | 0.028 | 2.4523 | 125 |
| ConA + Poly I:C | BDGlu | 48 | MO000025777 | STAT3(m) | 0.33018 | 0.033 | 2.3273 | 128 |
| ConA + Poly I:C | BDGlu | 48 | MO000170572 | IKK-beta(m){pS} | 0.25833 | 0 | 2.9415 | 128 |
| ConA + Poly I:C | BDGlu | 48 | MO000119664 | IRAK-4(m) | 0.26711 | 0.002 | 2.8127 | 129 |
| ConA + Poly I:C | BDGlu | 48 | MO000006539 | Hck(m) | 0.51232 | 0.011 | 1.9259 | 130 |
| ConA + Poly I:C | BDGlu | 48 | MO000018208 | FcgammaRI | 0.28907 | 0.003 | 2.5853 | 131 |
| ConA + Poly I:C | BDGlu | 48 | MO000062597 | FCRG(m) | 0.29266 | 0.006 | 2.5495 | 131 |
| ConA + Poly I:C | BDGlu | 48 | MO000165629 | IKK-beta(m){pS177}{pS181} | 0.26546 | 0.002 | 2.8493 | 131 |
| ConA + Poly I:C | BDGlu | 48 | MO000019402 | traf6(m) | 0.58017 | 0.038 | 1.8439 | 133 |
| ConA + Poly I:C | BDGlu | 48 | MO000039080 | IRAK-4(m) | 0.26726 | 0.002 | 2.7722 | 133 |
| ConA + Poly I:C | BDGlu | 48 | MO000033240 | EpoR(m) | 0.42886 | 0.009 | 1.9869 | 134 |
| ConA + Poly I:C | BDGlu | 48 | MO000256766 | PDGFRalpha(m) | 0.41277 | 0.014 | 1.9852 | 138 |
| ConA + Poly I:C | BDGlu | 48 | MO000088836 | NF-kappaB1(m) | 0.29787 | 0.037 | 2.2886 | 140 |
| ConA + Poly I:C | BDGlu | 48 | MO000037686 | Bmx(m) | 0.42241 | 0.026 | 1.9498 | 142 |
| ConA + Poly I:C | BDGlu | 48 | MO000245273 | Usp22(m) | 0.51657 | 0.026 | 1.8377 | 144 |
| ConA + Poly I:C | BDGlu | 48 | MO000037455 | Cas(m) | 0.34807 | 0.02 | 2.1429 | 145 |
| ConA + Poly I:C | BDGlu | 48 | MO000032104 | c-Kit(m){pY} | 0.25631 | 0.004 | 2.6566 | 147 |
| ConA + Poly I:C | BDGlu | 48 | MO000033795 | Tyk2(m) | 0.46529 | 0.014 | 1.8345 | 148 |
| ConA + Poly I:C | BDGlu | 48 | MO000038315 | LPS:lbp:CD14:TLR4:MD-2:TIRAP | 0.23311 | 0.002 | 2.8731 | 152 |
| ConA + Poly I:C | BDGlu | 48 | MO000079038 | SOCS-1(m) | 0.39534 | 0.022 | 1.9849 | 152 |
| ConA + Poly I:C | BDGlu | 48 | MO000130073 | PKCiota(m) | 0.23613 | 0.002 | 2.7837 | 153 |
| ConA + Poly I:C | BDGlu | 48 | MO000022549 | IKK-beta(m) | 0.52984 | 0.008 | 1.7741 | 154 |
| ConA + Poly I:C | BDGlu | 48 | MO000032972 | IRF-3(m) | 0.22413 | 0.029 | 3.4251 | 155 |
| ConA + Poly I:C | BDGlu | 48 | MO000098006 | Nod2(m) | 0.23935 | 0.001 | 2.693 | 156 |
| ConA + Poly I:C | BDGlu | 48 | MO000034986 | LMW-PTP(m) | 0.52439 | 0.035 | 1.7493 | 157 |
| ConA + Poly I:C | BDGlu | 48 | MO000033754 | PAK1(m) | 0.5777 | 0.029 | 1.6816 | 158 |
| ConA + Poly I:C | BDGlu | 48 | MO000056794 | PTP1B(m) | 0.63252 | 0.008 | 1.6723 | 159 |
| ConA + Poly I:C | BDGlu | 48 | MO000016669 | IFNRI | 0.2965 | 0.013 | 2.1763 | 160 |
| ConA + Poly I:C | BDGlu | 48 | MO000038554 | Epo:(EpoR{pY})2:Lyn | 0.23933 | 0.022 | 2.6507 | 160 |
| ConA + Poly I:C | BDGlu | 48 | MO000253996 | TBK1(m){pS172} | 0.21739 | 0.006 | 3.0386 | 161 |
| ConA + Poly I:C | BDGlu | 48 | MO000058103 | CD45(m) | 0.47764 | 0.05 | 1.6911 | 165 |
| ConA + Poly I:C | BDGlu | 48 | MO000034766 | HEF1(m) | 0.33978 | 0.033 | 2.0282 | 167 |
| ConA + Poly I:C | BDGlu | 48 | MO000059191 | AKT(m){p} | 0.40252 | 0.012 | 1.8646 | 167 |
| ConA + Poly I:C | BDGlu | 48 | MO000083014 | Hck-isoform1(m) | 0.28821 | 0.012 | 2.1965 | 167 |
| ConA + Poly I:C | BDGlu | 48 | MO000119981 | NIK(m) | 0.46063 | 0.013 | 1.6944 | 167 |
| ConA + Poly I:C | BDGlu | 48 | MO000008801 | Lyn(m) | 0.59594 | 0.026 | 1.4632 | 170 |
| ConA + Poly I:C | BDGlu | 48 | MO000020892 | Par3(m) | 0.24411 | 0.008 | 2.4297 | 170 |
| ConA + Poly I:C | BDGlu | 48 | MO000020192 | SHP-1(m) | 0.52627 | 0.05 | 1.6679 | 171 |
| ConA + Poly I:C | BDGlu | 48 | MO000022062 | Jak3(m) | 0.58922 | 0.049 | 1.4461 | 172 |
| ConA + Poly I:C | BDGlu | 48 | MO000023330 | E1(m) | 0.75667 | 0.044 | 1.1602 | 172 |
| ConA + Poly I:C | BDGlu | 48 | MO000120165 | IRF-3(m) | 0.20258 | 0.028 | 3.476 | 172 |
| ConA + Poly I:C | BDGlu | 48 | MO000121660 | alpha IIb-integrin(m):beta3-integrin(m) | 0.31449 | 0.02 | 2.0695 | 172 |
| ConA + Poly I:C | BDGlu | 48 | MO000013004 | Src(m) | 0.60985 | 0.048 | 1.3146 | 173 |
| ConA + Poly I:C | BDGlu | 48 | MO000013131 | STAT6(m) | 0.22529 | 0.038 | 2.7763 | 173 |
| ConA + Poly I:C | BDGlu | 48 | MO000057591 | PLCgamma2(m) | 0.25444 | 0.005 | 2.3176 | 173 |
| ConA + Poly I:C | BDGlu | 48 | MO000021511 | Nck-1(m) | 0.34559 | 0.029 | 1.9513 | 175 |
| ConA + Poly I:C | BDGlu | 48 | MO000114529 | Src(m){pY} | 0.40917 | 0.026 | 1.803 | 175 |
| ConA + Poly I:C | BDGlu | 48 | MO000001937 | Btk(m) | 0.29684 | 0.046 | 2.0745 | 176 |
| ConA + Poly I:C | BDGlu | 48 | MO000042840 | ptpn21(m) | 0.40215 | 0.019 | 1.8144 | 177 |
| ConA + Poly I:C | BDGlu | 48 | MO000017252 | IL-10R | 0.28836 | 0.011 | 2.1123 | 179 |
| ConA + Poly I:C | BDGlu | 48 | MO000019522 | IL-22R1:IL-10R2 | 0.28836 | 0.011 | 2.1123 | 179 |
| ConA + Poly I:C | BDGlu | 48 | MO000041137 | TRAM(m) | 0.20352 | 0.002 | 2.9739 | 179 |
| ConA + Poly I:C | BDGlu | 48 | MO000113198 | T6BP(m) | 0.37768 | 0.019 | 1.8932 | 179 |
| ConA + Poly I:C | BDGlu | 48 | MO000020145 | TRAF2(m) | 0.33088 | 0.027 | 1.97 | 180 |
| ConA + Poly I:C | BDGlu | 48 | MO000089140 | gp130(m) | 0.27752 | 0.008 | 2.1381 | 180 |
| ConA + Poly I:C | BDGlu | 48 | MO000032786 | IRS-1(m) | 0.34436 | 0.022 | 1.9042 | 184 |
| ConA + Poly I:C | BDGlu | 48 | MO000025653 | IRF-4(m) | 0.21199 | 0.039 | 2.7589 | 185 |
| ConA + Poly I:C | BDGlu | 48 | MO000020508 | HRS(m) | 0.36724 | 0.028 | 1.8628 | 188 |
| ConA + Poly I:C | BDGlu | 48 | MO000017453 | TAK1(m) | 0.44754 | 0.037 | 1.5538 | 190 |
| ConA + Poly I:C | BDGlu | 48 | MO000044385 | parkin(m) | 0.32462 | 0.038 | 1.9295 | 192 |
| ConA + Poly I:C | BDGlu | 48 | MO000088390 | IRF-8(m) | 0.20589 | 0.031 | 2.7125 | 192 |
| ConA + Poly I:C | BDGlu | 48 | MO000037747 | LynA(m) | 0.39443 | 0.023 | 1.7468 | 193 |
| ConA + Poly I:C | BDGlu | 48 | MO000022141 | traf6(m){ub{K63}(n)} | 0.20999 | 0.002 | 2.6117 | 194 |
| ConA + Poly I:C | BDGlu | 48 | MO000036067 | TRAF3(m) | 0.27649 | 0.015 | 2.0686 | 195 |
| ConA + Poly I:C | BDGlu | 48 | MO000037746 | LynB(m) | 0.39443 | 0.023 | 1.7468 | 195 |
| ConA + Poly I:C | BDGlu | 48 | MO000180126 | PP2Cbeta(m) | 0.44473 | 0.044 | 1.4368 | 195 |
| ConA + Poly I:C | BDGlu | 48 | MO000032890 | PTP-PEST(m) | 0.40419 | 0.023 | 1.6745 | 196 |
| ConA + Poly I:C | BDGlu | 48 | MO000032862 | PIAS3(m) | 0.43881 | 0.044 | 1.4418 | 197 |
| ConA + Poly I:C | BDGlu | 48 | MO000032836 | IFNAR2(m) | 0.26616 | 0.007 | 2.1049 | 198 |
| ConA + Poly I:C | BDGlu | 48 | MO000032852 | OSMRbeta(m) | 0.26614 | 0.007 | 2.1052 | 198 |
| ConA + Poly I:C | BDGlu | 48 | MO000128025 | traf6(m){ub}n | 0.20994 | 0.003 | 2.6009 | 198 |
| ConA + Poly I:C | BDGlu | 48 | MO000079450 | STAT5B(m) | 0.20499 | 0.034 | 2.6441 | 199 |
| ConA + Poly I:C | BDGlu | 48 | MO000120886 | traf6(m){ub} | 0.20994 | 0.003 | 2.6009 | 200 |
| ConA + Poly I:C | BDGlu | 48 | MO000188322 | E1{ub(1)}:Ubc13:Mms2 | 0.2328 | 0.008 | 2.2251 | 201 |
| ConA + Poly I:C | BDGlu | 48 | MO000019364 | RelA-p65(m) | 0.27028 | 0.031 | 2.0407 | 202 |
| ConA + Poly I:C | BDGlu | 48 | MO000160215 | E1:Ubc5A{ub(1)} | 0.2328 | 0.008 | 2.225 | 202 |
| ConA + Poly I:C | BDGlu | 48 | MO000021505 | Grb-2(m) | 0.35372 | 0.043 | 1.7819 | 203 |
| ConA + Poly I:C | BDGlu | 48 | MO000022224 | dsRNA | 0.24776 | 0.017 | 2.1379 | 203 |
| ConA + Poly I:C | BDGlu | 48 | MO000119264 | E1:UbcH7{ub(1)} | 0.2328 | 0.008 | 2.2252 | 203 |
| ConA + Poly I:C | BDGlu | 48 | MO000120961 | E1:Ubc7{ub(1)} | 0.2328 | 0.008 | 2.2251 | 203 |
| ConA + Poly I:C | BDGlu | 48 | MO000187670 | E1{ub(1)}:Ubc13:Uev1 | 0.2328 | 0.008 | 2.225 | 206 |
| ConA + Poly I:C | BDGlu | 48 | MO000020140 | TCR(m) | 0.27052 | 0.02 | 1.9932 | 207 |
| ConA + Poly I:C | BDGlu | 48 | MO000085462 | c-Cbl(m) | 0.29354 | 0.033 | 1.8994 | 207 |
| ConA + Poly I:C | BDGlu | 48 | MO000057306 | Src(m) | 0.39916 | 0.039 | 1.5733 | 209 |
| ConA + Poly I:C | BDGlu | 48 | MO000084528 | STAT5A(m) | 0.20499 | 0.036 | 2.5438 | 209 |
| ConA + Poly I:C | BDGlu | 48 | MO000037344 | IRSp53(m) | 0.29547 | 0.025 | 1.888 | 210 |
| ConA + Poly I:C | BDGlu | 48 | MO000021320 | InsR(m) | 0.39011 | 0.04 | 1.6677 | 211 |
| ConA + Poly I:C | BDGlu | 48 | MO000007259 | IGF-2(m) | 0.27709 | 0.029 | 1.9296 | 214 |
| ConA + Poly I:C | BDGlu | 48 | MO000084046 | CD19(m) | 0.36145 | 0.046 | 1.68 | 215 |
| ConA + Poly I:C | BDGlu | 48 | MO000057490 | InsR(m){pY} | 0.24069 | 0.018 | 2.0761 | 217 |
| ConA + Poly I:C | BDGlu | 48 | MO000120050 | UBE2G2(m) | 0.2326 | 0.01 | 2.1812 | 218 |
| ConA + Poly I:C | BDGlu | 48 | MO000084602 | IKK-gamma(m) | 0.21668 | 0.009 | 2.2164 | 220 |
| ConA + Poly I:C | BDGlu | 48 | MO000127428 | Rock-1(m) | 0.29365 | 0.022 | 1.834 | 221 |
| ConA + Poly I:C | BDGlu | 48 | MO000151911 | IKK-alpha(m){p} | 0.28611 | 0.032 | 1.8871 | 221 |
| ConA + Poly I:C | BDGlu | 48 | MO000019250 | RIP(m) | 0.24046 | 0.025 | 2.0629 | 222 |
| ConA + Poly I:C | BDGlu | 48 | MO000082402 | MEKK1-xbb2(m) | 0.33503 | 0.046 | 1.6797 | 224 |
| ConA + Poly I:C | BDGlu | 48 | MO000032784 | PLCgamma1(m) | 0.2464 | 0.01 | 1.9895 | 226 |
| ConA + Poly I:C | BDGlu | 48 | MO000002525 | CD28(m) | 0.26381 | 0.02 | 1.9336 | 227 |
| ConA + Poly I:C | BDGlu | 48 | MO000042988 | Nrdp1(m) | 0.21512 | 0.012 | 2.1832 | 227 |
| ConA + Poly I:C | BDGlu | 48 | MO000056839 | c-Kit(m) | 0.2337 | 0.023 | 2.0336 | 231 |
| ConA + Poly I:C | BDGlu | 48 | MO000044233 | Jak2(m){pY1007} | 0.24564 | 0.034 | 1.9567 | 232 |
| ConA + Poly I:C | BDGlu | 48 | MO000075867 | MEKK1(m) | 0.33503 | 0.047 | 1.6584 | 233 |
| ConA + Poly I:C | BDGlu | 48 | MO000038127 | Src(m){pY424} | 0.21255 | 0.01 | 2.1594 | 234 |
| ConA + Poly I:C | BDGlu | 48 | MO000056866 | EpoR-F(m) | 0.22432 | 0.017 | 2.1165 | 234 |
| ConA + Poly I:C | BDGlu | 48 | MO000060232 | Src(m){p} | 0.21255 | 0.01 | 2.1594 | 235 |
| ConA + Poly I:C | BDGlu | 48 | MO000115111 | Src(m){pY527} | 0.21255 | 0.01 | 2.1594 | 236 |
| ConA + Poly I:C | BDGlu | 48 | MO000020142 | BCR(m) | 0.24935 | 0.012 | 1.8985 | 240 |
| ConA + Poly I:C | BDGlu | 48 | MO000149349 | RNF126(m) | 0.26991 | 0.029 | 1.8204 | 242 |
| ConA + Poly I:C | BDGlu | 48 | MO000240859 | Rnf115(m) | 0.26991 | 0.029 | 1.8202 | 242 |
| ConA + Poly I:C | BDGlu | 48 | MO000236690 | Mad2l1bp(m) | 0.24666 | 0.018 | 1.8928 | 244 |
| ConA + Poly I:C | BDGlu | 48 | MO000036021 | EDAR(m) | 0.23865 | 0.016 | 1.8991 | 249 |
| ConA + Poly I:C | BDGlu | 48 | MO000037328 | dock9(m) | 0.27619 | 0.037 | 1.7403 | 250 |
| ConA + Poly I:C | BDGlu | 48 | MO000057352 | PTP1B(m){pY} | 0.20639 | 0.013 | 1.997 | 265 |
| ConA + Poly I:C | BDGlu | 48 | MO000107884 | Cbl-b(m) | 0.27533 | 0.048 | 1.6062 | 267 |
| ConA + Poly I:C | BDGlu | 48 | MO000036576 | BCAR-3(m) | 0.23273 | 0.019 | 1.8806 | 268 |
| ConA + Poly I:C | BDGlu | 48 | MO000166501 | CpG B | 0.23069 | 0.028 | 1.8787 | 272 |
| ConA + Poly I:C | BDGlu | 48 | MO000056726 | FRS2alpha(m) | 0.23321 | 0.025 | 1.7789 | 277 |
| ConA + Poly I:C | BDGlu | 48 | MO000114548 | FRS3(m) | 0.23321 | 0.025 | 1.777 | 277 |
| ConA + Poly I:C | BDGlu | 48 | MO000095704 | TRIF(m) | 0.25234 | 0.042 | 1.6641 | 281 |
| ConA + Poly I:C | BDGlu | 48 | MO000032774 | RIP1(m) | 0.22618 | 0.021 | 1.8135 | 286 |
| ConA + Poly I:C | BDGlu | 48 | MO000130207 | Arhgef9(m) | 0.22617 | 0.021 | 1.8139 | 286 |
| ConA + Poly I:C | BDGlu | 48 | MO000199377 | Fgd1(m) | 0.22618 | 0.021 | 1.8132 | 286 |
| ConA + Poly I:C | BDGlu | 48 | MO000097965 | trkA(m) | 0.23511 | 0.031 | 1.6764 | 288 |
| ConA + Poly I:C | BDGlu | 48 | MO000285932 | Cezanne(m) | 0.20551 | 0.038 | 1.8411 | 296 |
| ConA + Poly I:C | BDGlu | 48 | MO000033731 | Sprouty2(m) | 0.23146 | 0.048 | 1.6819 | 297 |
| ConA + Poly I:C | BDGlu | 48 | MO000032886 | C3G(m) | 0.23318 | 0.041 | 1.6047 | 301 |
| ConA + Poly I:C | BDGlu | 48 | MO000186097 | RPTPalpha(m){pS204} | 0.21235 | 0.042 | 1.6735 | 317 |
| ConA + Poly I:C | BDGlu | 48 | MO000068857 | Omi(m) | 0.20232 | 0.04 | 1.6891 | 322 |
| ConA + Poly I:C | BDGlu | 96 | MO000165399 | IKK-i(m){p} | 0.53797 | 0.002 | 2.7295 | 7 |
| ConA + Poly I:C | BDGlu | 96 | MO000106371 | CaMKII(m) | 0.44766 | 0.007 | 2.369 | 12 |
| ConA + Poly I:C | BDGlu | 96 | MO000036158 | TBK1(m):TANK(m) | 0.39954 | 0.008 | 4.8127 | 13 |
| ConA + Poly I:C | BDGlu | 96 | MO000120165 | IRF-3(m) | 0.40871 | 0.002 | 3.3113 | 13 |
| ConA + Poly I:C | BDGlu | 96 | MO000149650 | TBK1(m) | 0.43632 | 0.013 | 2.4294 | 13 |
| ConA + Poly I:C | BDGlu | 96 | MO000253996 | TBK1(m){pS172} | 0.39906 | 0.004 | 3.1607 | 16 |
| ConA + Poly I:C | BDGlu | 96 | MO000122582 | IKK-i(m) | 0.43388 | 0.016 | 1.8624 | 25 |
| ConA + Poly I:C | BDGlu | 96 | MO000032972 | IRF-3(m) | 0.3689 | 0.005 | 2.9342 | 27 |
| ConA + Poly I:C | BDGlu | 96 | MO000019531 | RSK2(m) | 0.49894 | 0.021 | 1.4341 | 34 |
| ConA + Poly I:C | BDGlu | 96 | MO000022549 | IKK-beta(m) | 0.55864 | 0.025 | 1.3949 | 34 |
| ConA + Poly I:C | BDGlu | 96 | MO000019336 | TBK1(m) | 0.60733 | 0.006 | 1.3802 | 35 |
| ConA + Poly I:C | BDGlu | 96 | MO000044514 | CHIP(m) | 0.44496 | 0.031 | 1.5027 | 35 |
| ConA + Poly I:C | BDGlu | 96 | MO000151911 | IKK-alpha(m){p} | 0.38627 | 0.022 | 1.926 | 35 |
| ConA + Poly I:C | BDGlu | 96 | MO000019329 | TANK(m) | 0.32331 | 0.008 | 2.1482 | 36 |
| ConA + Poly I:C | BDGlu | 96 | MO000120015 | TLR3(m) | 0.31621 | 0.008 | 2.183 | 37 |
| ConA + Poly I:C | BDGlu | 96 | MO000127428 | Rock-1(m) | 0.3847 | 0.026 | 1.6855 | 40 |
| ConA + Poly I:C | BDGlu | 96 | MO000079423 | GSK3alpha(m) | 0.42878 | 0.021 | 1.3381 | 48 |
| ConA + Poly I:C | BDGlu | 96 | MO000122350 | TANK-isoform2(m) | 0.24897 | 0.007 | 3.1135 | 48 |
| ConA + Poly I:C | BDGlu | 96 | MO000084602 | IKK-gamma(m) | 0.27518 | 0.033 | 1.8777 | 51 |
| ConA + Poly I:C | BDGlu | 96 | MO000057073 | GSK3beta(m) | 0.42279 | 0.034 | 1.2022 | 54 |
| ConA + Poly I:C | BDGlu | 96 | MO000057953 | p70S6K1(m) | 0.44957 | 0.039 | 1.0506 | 54 |
| ConA + Poly I:C | BDGlu | 96 | MO000233616 | Gprc5b(m) | 0.26865 | 0.017 | 2.0547 | 55 |
| ConA + Poly I:C | BDGlu | 96 | MO000020525 | Bax(m) | 0.3081 | 0.018 | 1.6137 | 56 |
| ConA + Poly I:C | BDGlu | 96 | MO000120308 | CaMKII-alpha(m) | 0.35951 | 0.01 | 1.4122 | 56 |
| ConA + Poly I:C | BDGlu | 96 | MO000167333 | Laforin(m) | 0.39761 | 0.047 | 1.2896 | 56 |
| ConA + Poly I:C | BDGlu | 96 | MO000041182 | TRIF(m) | 0.41832 | 0.012 | 1.1126 | 57 |
| ConA + Poly I:C | BDGlu | 96 | MO000106376 | PP1(m) | 0.29603 | 0.048 | 1.6207 | 57 |
| ConA + Poly I:C | BDGlu | 96 | MO000120915 | IKK-beta(m){pS181} | 0.21812 | 0.023 | 2.2694 | 58 |
| ConA + Poly I:C | BDGlu | 96 | MO000007943 | CaMKII-alpha(m) | 0.30425 | 0.027 | 1.5807 | 59 |
| ConA + Poly I:C | BDGlu | 96 | MO000057122 | GSK3beta(m){pS9} | 0.30909 | 0.02 | 1.4977 | 59 |
| ConA + Poly I:C | BDGlu | 96 | MO000120961 | E1:Ubc7{ub(1)} | 0.27241 | 0.046 | 1.6339 | 59 |
| ConA + Poly I:C | BDGlu | 96 | MO000188322 | E1{ub(1)}:Ubc13:Mms2 | 0.27241 | 0.046 | 1.6262 | 59 |
| ConA + Poly I:C | BDGlu | 96 | MO000117364 | AMPKalpha-1(m) | 0.27101 | 0.03 | 1.6697 | 61 |
| ConA + Poly I:C | BDGlu | 96 | MO000120050 | UBE2G2(m) | 0.27219 | 0.047 | 1.6521 | 61 |
| ConA + Poly I:C | BDGlu | 96 | MO000131412 | E1:Ubc5C{ub(1)} | 0.23524 | 0.033 | 1.8967 | 61 |
| ConA + Poly I:C | BDGlu | 96 | MO000117366 | AMPKalpha-1(m) | 0.39114 | 0.04 | 1.2691 | 62 |
| ConA + Poly I:C | BDGlu | 96 | MO000122489 | mTOR(m):raptor(m) | 0.34959 | 0.05 | 1.3574 | 63 |
| ConA + Poly I:C | BDGlu | 96 | MO000233615 | Gprc5b(m) | 0.20436 | 0.026 | 2.0988 | 63 |
| ConA + Poly I:C | BDGlu | 96 | MO000121392 | IKK-beta(m){p} | 0.21761 | 0.033 | 2.0544 | 64 |
| ConA + Poly I:C | BDGlu | 96 | MO000035087 | CnAbeta(m) | 0.39726 | 0.045 | 1.0514 | 67 |
| ConA + Poly I:C | BDGlu | 96 | MO000106362 | PKCepsilon(m) | 0.38997 | 0.041 | 1.0816 | 68 |
| ConA + Poly I:C | BDGlu | 96 | MO000119264 | E1:UbcH7{ub(1)} | 0.27241 | 0.046 | 1.5345 | 68 |
| ConA + Poly I:C | BDGlu | 96 | MO000128191 | AKT-1(m){pS473} | 0.3976 | 0.047 | 1.0453 | 68 |
| ConA + Poly I:C | BDGlu | 96 | MO000339319 | AKT-1(m){p} | 0.3976 | 0.047 | 1.0453 | 68 |
| ConA + Poly I:C | BDGlu | 96 | MO000079452 | GSK3beta(m){pS} | 0.30907 | 0.021 | 1.3253 | 70 |
| ConA + Poly I:C | BDGlu | 96 | MO000160215 | E1:Ubc5A{ub(1)} | 0.27241 | 0.046 | 1.3921 | 70 |
| ConA + Poly I:C | BDGlu | 96 | MO000095704 | TRIF(m) | 0.32282 | 0.021 | 1.1218 | 72 |
| ConA + Poly I:C | BDGlu | 96 | MO000106366 | CaMKII-beta(m) | 0.23321 | 0.044 | 1.6055 | 73 |
| ConA + Poly I:C | BDGlu | 96 | MO000094999 | ZIPK(m) | 0.23406 | 0.019 | 1.5367 | 74 |
| ConA + Poly I:C | BDGlu | 96 | MO000058796 | Bax-alpha(m) | 0.30906 | 0.008 | 1.0909 | 78 |
| ConA + Poly I:C | BDGlu | 96 | MO000170572 | IKK-beta(m){pS} | 0.25204 | 0.046 | 1.375 | 80 |
| ConA + Poly I:C | BDGlu | 96 | MO000187670 | E1{ub(1)}:Ubc13:Uev1 | 0.27241 | 0.046 | 1.2731 | 81 |
| ConA + Poly I:C | BDGlu | 96 | MO000041137 | TRAM(m) | 0.2231 | 0.025 | 1.362 | 86 |
| ConA + Poly I:C | BDGlu | 96 | MO000019336 | TBK1(m) | 0.5304 | 0.001 | 2.5726 | 22 |
| ConA + Poly I:C | BDGlu | 96 | MO000089823 | cIAP-2(m) | 0.40966 | 0.02 | 3.3967 | 26 |
| ConA + Poly I:C | BDGlu | 96 | MO000007348 | IL-2Rbeta(m) | 0.42187 | 0 | 2.3766 | 41 |
| ConA + Poly I:C | BDGlu | 96 | MO000041182 | TRIF(m) | 0.38678 | 0.002 | 2.7069 | 43 |
| ConA + Poly I:C | BDGlu | 96 | MO000032271 | SOCS-1(m) | 0.43046 | 0.002 | 2.1267 | 53 |
| ConA + Poly I:C | BDGlu | 96 | MO000089319 | Caspase(m) | 0.43132 | 0.031 | 2.093 | 54 |
| ConA + Poly I:C | BDGlu | 96 | MO000017162 | c-Kit(m) | 0.44794 | 0.012 | 2.059 | 58 |
| ConA + Poly I:C | BDGlu | 96 | MO000059714 | ErbB3(m) | 0.37501 | 0.023 | 2.348 | 59 |
| ConA + Poly I:C | BDGlu | 96 | MO000024810 | ER-alpha(m) | 0.29355 | 0.024 | 3.5447 | 60 |
| ConA + Poly I:C | BDGlu | 96 | MO000149650 | TBK1(m) | 0.30274 | 0.004 | 2.7901 | 63 |
| ConA + Poly I:C | BDGlu | 96 | MO000025777 | STAT3(m) | 0.34621 | 0.027 | 2.4177 | 65 |
| ConA + Poly I:C | BDGlu | 96 | MO000117511 | TC-PTPa(m) | 0.51872 | 0.015 | 1.9284 | 66 |
| ConA + Poly I:C | BDGlu | 96 | MO000013127 | STAT5A(m) | 0.28912 | 0.015 | 3.0359 | 67 |
| ConA + Poly I:C | BDGlu | 96 | MO000013134 | STAT5B(m) | 0.28638 | 0.015 | 3.1528 | 68 |
| ConA + Poly I:C | BDGlu | 96 | MO000019485 | IFNalpha, IFNbeta:IFNAR1{pY}:Tyk2{pY}:IFNAR2c{pY}:Jak1{pY} | 0.32051 | 0.01 | 2.5552 | 68 |
| ConA + Poly I:C | BDGlu | 96 | MO000128620 | EAC(m) | 0.42051 | 0.012 | 2.0245 | 69 |
| ConA + Poly I:C | BDGlu | 96 | MO000007226 | IGF-1R(m) | 0.44436 | 0.019 | 1.9593 | 71 |
| ConA + Poly I:C | BDGlu | 96 | MO000016606 | IKK-gamma(m) | 0.3618 | 0.025 | 2.2113 | 72 |
| ConA + Poly I:C | BDGlu | 96 | MO000022056 | Jak1(m) | 0.53898 | 0.006 | 1.8661 | 75 |
| ConA + Poly I:C | BDGlu | 96 | MO000120015 | TLR3(m) | 0.26763 | 0 | 3.3391 | 75 |
| ConA + Poly I:C | BDGlu | 96 | MO000079038 | SOCS-1(m) | 0.38946 | 0.019 | 2.021 | 77 |
| ConA + Poly I:C | BDGlu | 96 | MO000079029 | Jak1(m) | 0.32185 | 0.015 | 2.3045 | 78 |
| ConA + Poly I:C | BDGlu | 96 | MO000234738 | Irak2(m) | 0.31141 | 0.015 | 2.3223 | 78 |
| ConA + Poly I:C | BDGlu | 96 | MO000020508 | HRS(m) | 0.36735 | 0.015 | 2.0748 | 79 |
| ConA + Poly I:C | BDGlu | 96 | MO000032775 | p75NTR(m) | 0.31095 | 0.013 | 2.2972 | 83 |
| ConA + Poly I:C | BDGlu | 96 | MO000088836 | NF-kappaB1(m) | 0.29608 | 0.038 | 2.3222 | 84 |
| ConA + Poly I:C | BDGlu | 96 | MO000033240 | EpoR(m) | 0.41717 | 0.009 | 1.922 | 86 |
| ConA + Poly I:C | BDGlu | 96 | MO000007259 | IGF-2(m) | 0.28163 | 0.008 | 2.3718 | 89 |
| ConA + Poly I:C | BDGlu | 96 | MO000086151 | traf6-isoform1(m) | 0.37705 | 0.033 | 1.9429 | 92 |
| ConA + Poly I:C | BDGlu | 96 | MO000156307 | FcepsilonRI beta(m) | 0.31345 | 0.018 | 2.0797 | 92 |
| ConA + Poly I:C | BDGlu | 96 | MO000001937 | Btk(m) | 0.29466 | 0.034 | 2.1257 | 94 |
| ConA + Poly I:C | BDGlu | 96 | MO000021719 | A20(m) | 0.37772 | 0.025 | 1.9264 | 94 |
| ConA + Poly I:C | BDGlu | 96 | MO000022549 | IKK-beta(m) | 0.53333 | 0.006 | 1.746 | 94 |
| ConA + Poly I:C | BDGlu | 96 | MO000006539 | Hck(m) | 0.49119 | 0.018 | 1.7729 | 96 |
| ConA + Poly I:C | BDGlu | 96 | MO000120045 | UbcH7(m) | 0.4297 | 0.036 | 1.8374 | 96 |
| ConA + Poly I:C | BDGlu | 96 | MO000021349 | RPTPalpha(m) | 0.43419 | 0.025 | 1.8181 | 97 |
| ConA + Poly I:C | BDGlu | 96 | MO000033795 | Tyk2(m) | 0.4667 | 0.026 | 1.7668 | 99 |
| ConA + Poly I:C | BDGlu | 96 | MO000037686 | Bmx(m) | 0.41111 | 0.032 | 1.8434 | 99 |
| ConA + Poly I:C | BDGlu | 96 | MO000120044 | UbcH7(m) | 0.28851 | 0.03 | 2.0924 | 100 |
| ConA + Poly I:C | BDGlu | 96 | MO000017453 | TAK1(m) | 0.46457 | 0.034 | 1.7578 | 101 |
| ConA + Poly I:C | BDGlu | 96 | MO000200602 | march5(m) | 0.36151 | 0.029 | 1.9225 | 103 |
| ConA + Poly I:C | BDGlu | 96 | MO000032104 | c-Kit(m){pY} | 0.24371 | 0.002 | 2.6002 | 105 |
| ConA + Poly I:C | BDGlu | 96 | MO000037344 | IRSp53(m) | 0.29165 | 0.021 | 2.0409 | 107 |
| ConA + Poly I:C | BDGlu | 96 | MO000098007 | Nod2(m) | 0.35486 | 0.024 | 1.9072 | 110 |
| ConA + Poly I:C | BDGlu | 96 | MO000032013 | Jak2(m){p} | 0.2786 | 0.037 | 2.0616 | 112 |
| ConA + Poly I:C | BDGlu | 96 | MO000245273 | Usp22(m) | 0.49779 | 0.034 | 1.6938 | 112 |
| ConA + Poly I:C | BDGlu | 96 | MO000256766 | PDGFRalpha(m) | 0.39895 | 0.024 | 1.7721 | 112 |
| ConA + Poly I:C | BDGlu | 96 | MO000007186 | IRF-8(m) | 0.22181 | 0.04 | 2.8027 | 115 |
| ConA + Poly I:C | BDGlu | 96 | MO000022316 | PKCiota(m) | 0.38294 | 0.026 | 1.7834 | 115 |
| ConA + Poly I:C | BDGlu | 96 | MO000032972 | IRF-3(m) | 0.21902 | 0.027 | 3.2446 | 117 |
| ConA + Poly I:C | BDGlu | 96 | MO000245279 | Usp25(m) | 0.35683 | 0.025 | 1.8449 | 117 |
| ConA + Poly I:C | BDGlu | 96 | MO000038316 | LPS:lbp:CD14:TLR4:MD-2:TIRAP:IRAK-2 | 0.38983 | 0.019 | 1.7452 | 118 |
| ConA + Poly I:C | BDGlu | 96 | MO000039080 | IRAK-4(m) | 0.24667 | 0.007 | 2.2687 | 118 |
| ConA + Poly I:C | BDGlu | 96 | MO000041437 | dsRNA:TLR3:TRIF | 0.38983 | 0.019 | 1.7457 | 118 |
| ConA + Poly I:C | BDGlu | 96 | MO000037455 | Cas(m) | 0.33248 | 0.034 | 1.8721 | 119 |
| ConA + Poly I:C | BDGlu | 96 | MO000020892 | Par3(m) | 0.23625 | 0.008 | 2.3699 | 120 |
| ConA + Poly I:C | BDGlu | 96 | MO000081385 | TAK1(m) | 0.28542 | 0.019 | 1.9666 | 120 |
| ConA + Poly I:C | BDGlu | 96 | MO000013131 | STAT6(m) | 0.21981 | 0.036 | 2.7577 | 121 |
| ConA + Poly I:C | BDGlu | 96 | MO000032786 | IRS-1(m) | 0.33528 | 0.032 | 1.8479 | 121 |
| ConA + Poly I:C | BDGlu | 96 | MO000059659 | IRAK-1(m){p} | 0.35253 | 0.031 | 1.8372 | 122 |
| ConA + Poly I:C | BDGlu | 96 | MO000119664 | IRAK-4(m) | 0.24345 | 0.012 | 2.2272 | 122 |
| ConA + Poly I:C | BDGlu | 96 | MO000056794 | PTP1B(m) | 0.62295 | 0.012 | 1.5691 | 123 |
| ConA + Poly I:C | BDGlu | 96 | MO000008302 | Syk(m) | 0.5027 | 0.047 | 1.6244 | 124 |
| ConA + Poly I:C | BDGlu | 96 | MO000121034 | CIKS(m) | 0.35247 | 0.033 | 1.8291 | 124 |
| ConA + Poly I:C | BDGlu | 96 | MO000017821 | Igalpha:Igbeta | 0.26142 | 0.016 | 2.0155 | 126 |
| ConA + Poly I:C | BDGlu | 96 | MO000038554 | Epo:(EpoR{pY})2:Lyn | 0.22281 | 0.025 | 2.4058 | 126 |
| ConA + Poly I:C | BDGlu | 96 | MO000033358 | PKCdelta(m) | 0.65741 | 0.045 | 1.3362 | 127 |
| ConA + Poly I:C | BDGlu | 96 | MO000088390 | IRF-8(m) | 0.21666 | 0.036 | 2.763 | 127 |
| ConA + Poly I:C | BDGlu | 96 | MO000003498 | Csk(m) | 0.50004 | 0.048 | 1.5814 | 128 |
| ConA + Poly I:C | BDGlu | 96 | MO000023372 | SIRT1(m) | 0.69384 | 0.046 | 1.1288 | 128 |
| ConA + Poly I:C | BDGlu | 96 | MO000056936 | SIRT1-isoform1(m) | 0.63897 | 0.046 | 1.2131 | 129 |
| ConA + Poly I:C | BDGlu | 96 | MO000336179 | TAK1(m){ub} | 0.2962 | 0.034 | 1.8553 | 129 |
| ConA + Poly I:C | BDGlu | 96 | MO000025653 | IRF-4(m) | 0.20777 | 0.034 | 2.7792 | 130 |
| ConA + Poly I:C | BDGlu | 96 | MO000253996 | TBK1(m){pS172} | 0.20437 | 0.008 | 2.8273 | 130 |
| ConA + Poly I:C | BDGlu | 96 | MO000166537 | FcgammaRIII(m) | 0.26626 | 0.023 | 1.9267 | 134 |
| ConA + Poly I:C | BDGlu | 96 | MO000200601 | march5(m) | 0.3615 | 0.044 | 1.7357 | 134 |
| ConA + Poly I:C | BDGlu | 96 | MO000017252 | IL-10R | 0.27149 | 0.024 | 1.9184 | 135 |
| ConA + Poly I:C | BDGlu | 96 | MO000019522 | IL-22R1:IL-10R2 | 0.27148 | 0.024 | 1.9184 | 135 |
| ConA + Poly I:C | BDGlu | 96 | MO000019364 | RelA-p65(m) | 0.25526 | 0.05 | 1.9768 | 136 |
| ConA + Poly I:C | BDGlu | 96 | MO000165629 | IKK-beta(m){pS177}{pS181} | 0.2337 | 0.017 | 2.0887 | 137 |
| ConA + Poly I:C | BDGlu | 96 | MO000018208 | FcgammaRI | 0.25908 | 0.016 | 1.959 | 138 |
| ConA + Poly I:C | BDGlu | 96 | MO000086968 | HPK1(m) | 0.30155 | 0.041 | 1.814 | 138 |
| ConA + Poly I:C | BDGlu | 96 | MO000180126 | PP2Cbeta(m) | 0.43874 | 0.038 | 1.5464 | 139 |
| ConA + Poly I:C | BDGlu | 96 | MO000079450 | STAT5B(m) | 0.20045 | 0.03 | 2.7484 | 140 |
| ConA + Poly I:C | BDGlu | 96 | MO000121772 | A20(m) | 0.38309 | 0.04 | 1.666 | 140 |
| ConA + Poly I:C | BDGlu | 96 | MO000084528 | STAT5A(m) | 0.20045 | 0.031 | 2.6954 | 141 |
| ConA + Poly I:C | BDGlu | 96 | MO000044233 | Jak2(m){pY1007} | 0.24326 | 0.027 | 1.9994 | 142 |
| ConA + Poly I:C | BDGlu | 96 | MO000062597 | FCRG(m) | 0.26473 | 0.027 | 1.9061 | 143 |
| ConA + Poly I:C | BDGlu | 96 | MO000170572 | IKK-beta(m){pS} | 0.22856 | 0.016 | 2.0817 | 144 |
| ConA + Poly I:C | BDGlu | 96 | MO000056839 | c-Kit(m) | 0.22971 | 0.022 | 2.0683 | 145 |
| ConA + Poly I:C | BDGlu | 96 | MO000036267 | pld2(m) | 0.20374 | 0.005 | 2.3742 | 146 |
| ConA + Poly I:C | BDGlu | 96 | MO000016669 | IFNRI | 0.27879 | 0.032 | 1.8147 | 149 |
| ConA + Poly I:C | BDGlu | 96 | MO000026727 | FXR(m) | 0.20664 | 0.027 | 2.3207 | 149 |
| ConA + Poly I:C | BDGlu | 96 | MO000038315 | LPS:lbp:CD14:TLR4:MD-2:TIRAP | 0.21687 | 0.013 | 2.1542 | 149 |
| ConA + Poly I:C | BDGlu | 96 | MO000113198 | T6BP(m) | 0.36395 | 0.038 | 1.6499 | 151 |
| ConA + Poly I:C | BDGlu | 96 | MO000041135 | tab2(m) | 0.25416 | 0.022 | 1.8856 | 153 |
| ConA + Poly I:C | BDGlu | 96 | MO000119721 | tab3(m) | 0.2542 | 0.022 | 1.8845 | 153 |
| ConA + Poly I:C | BDGlu | 96 | MO000044588 | parkin:Cul-1:Fbw7 | 0.22872 | 0.022 | 1.9854 | 155 |
| ConA + Poly I:C | BDGlu | 96 | MO000037747 | LynA(m) | 0.37252 | 0.027 | 1.548 | 157 |
| ConA + Poly I:C | BDGlu | 96 | MO000037746 | LynB(m) | 0.37252 | 0.027 | 1.548 | 159 |
| ConA + Poly I:C | BDGlu | 96 | MO000127428 | Rock-1(m) | 0.2834 | 0.041 | 1.7181 | 164 |
| ConA + Poly I:C | BDGlu | 96 | MO000084602 | IKK-gamma(m) | 0.21017 | 0.011 | 2.0548 | 165 |
| ConA + Poly I:C | BDGlu | 96 | MO000017705 | FcepsilonRI | 0.26888 | 0.035 | 1.7218 | 166 |
| ConA + Poly I:C | BDGlu | 96 | MO000095704 | TRIF(m) | 0.25066 | 0.037 | 1.776 | 170 |
| ConA + Poly I:C | BDGlu | 96 | MO000056866 | EpoR-F(m) | 0.21486 | 0.022 | 1.9775 | 171 |
| ConA + Poly I:C | BDGlu | 96 | MO000042988 | Nrdp1(m) | 0.2057 | 0.022 | 1.972 | 177 |
| ConA + Poly I:C | BDGlu | 96 | MO000036067 | TRAF3(m) | 0.2566 | 0.043 | 1.7027 | 183 |
| ConA + Poly I:C | BDGlu | 96 | MO000149349 | RNF126(m) | 0.26048 | 0.036 | 1.6885 | 184 |
| ConA + Poly I:C | BDGlu | 96 | MO000240859 | Rnf115(m) | 0.26049 | 0.036 | 1.6884 | 184 |
| ConA + Poly I:C | BDGlu | 96 | MO000151911 | IKK-alpha(m){p} | 0.26581 | 0.046 | 1.6595 | 185 |
| ConA + Poly I:C | BDGlu | 96 | MO000032836 | IFNAR2(m) | 0.23822 | 0.027 | 1.7197 | 190 |
| ConA + Poly I:C | BDGlu | 96 | MO000032852 | OSMRbeta(m) | 0.23821 | 0.027 | 1.7201 | 190 |
| ConA + Poly I:C | BDGlu | 96 | MO000083014 | Hck-isoform1(m) | 0.26066 | 0.047 | 1.6395 | 192 |
| ConA + Poly I:C | BDGlu | 96 | MO000098006 | Nod2(m) | 0.21826 | 0.026 | 1.8245 | 194 |
| ConA + Poly I:C | BDGlu | 96 | MO000057591 | PLCgamma2(m) | 0.23179 | 0.041 | 1.7227 | 195 |
| ConA + Poly I:C | BDGlu | 96 | MO000022224 | dsRNA | 0.23287 | 0.029 | 1.7181 | 197 |
| ConA + Poly I:C | BDGlu | 96 | MO000010243 | leptin(m) | 0.23462 | 0.04 | 1.7068 | 198 |
| ConA + Poly I:C | BDGlu | 96 | MO000036516 | Rac1(m):GTP | 0.23632 | 0.03 | 1.6934 | 199 |
| ConA + Poly I:C | BDGlu | 96 | MO000032784 | PLCgamma1(m) | 0.23186 | 0.036 | 1.7149 | 200 |
| ConA + Poly I:C | BDGlu | 96 | MO000043647 | Tome-1(m) | 0.2438 | 0.034 | 1.6545 | 200 |
| ConA + Poly I:C | BDGlu | 96 | MO000002525 | CD28(m) | 0.2504 | 0.05 | 1.605 | 204 |
| ConA + Poly I:C | BDGlu | 96 | MO000089140 | gp130(m) | 0.24307 | 0.044 | 1.5825 | 211 |
| ConA + Poly I:C | BDGlu | 96 | MO000032774 | RIP1(m) | 0.21919 | 0.038 | 1.6866 | 219 |
| ConA + Poly I:C | BDGlu | 96 | MO000032835 | IL-7(m) | 0.22827 | 0.05 | 1.6485 | 219 |
| ConA + Poly I:C | BDGlu | 96 | MO000130207 | Arhgef9(m) | 0.21919 | 0.038 | 1.6868 | 219 |
| ConA + Poly I:C | BDGlu | 96 | MO000199377 | Fgd1(m) | 0.2192 | 0.038 | 1.6865 | 219 |
| ConA + Poly I:C | BDGlu | 96 | MO000057490 | InsR(m){pY} | 0.22154 | 0.043 | 1.6328 | 224 |
| ConA + Poly I:C | BDGlu | 96 | MO000032843 | CrkL(m) | 0.21653 | 0.04 | 1.4873 | 243 |
| ConA + Poly I:C | BDGlu | 96 | MO000186097 | RPTPalpha(m){pS204} | 0.20115 | 0.047 | 1.5027 | 250 |
| ConA | Kag30 | 48 | MO000024810 | ER-alpha(m) | 0.37704 | 0.003 | 3.3365 | 22 |
| ConA | Kag30 | 48 | MO000045693 | Hsp90-beta(m) | 0.40449 | 0.018 | 2.0506 | 24 |
| ConA | Kag30 | 48 | MO000021511 | Nck-1(m) | 0.39906 | 0.005 | 2.1609 | 25 |
| ConA | Kag30 | 48 | MO000035474 | Cdc42(m) | 0.45829 | 0.001 | 1.7529 | 26 |
| ConA | Kag30 | 48 | MO000094162 | Hsp90-alpha(m) | 0.40425 | 0.019 | 1.9738 | 28 |
| ConA | Kag30 | 48 | MO000056800 | RXR-alpha(m) | 0.34285 | 0.022 | 3.137 | 30 |
| ConA | Kag30 | 48 | MO000044514 | CHIP(m) | 0.44113 | 0.029 | 1.6638 | 31 |
| ConA | Kag30 | 48 | MO000025590 | RXR-alpha(m) | 0.34258 | 0.033 | 2.8473 | 32 |
| ConA | Kag30 | 48 | MO000028472 | SRC-1(m) | 0.34438 | 0.014 | 2.3655 | 33 |
| ConA | Kag30 | 48 | MO000123092 | MAPKAPK2(m) | 0.42125 | 0.011 | 1.6338 | 35 |
| ConA | Kag30 | 48 | MO000245273 | Usp22(m) | 0.66929 | 0 | 1.4724 | 39 |
| ConA | Kag30 | 48 | MO000078606 | PIAS1(m) | 0.57309 | 0.017 | 1.5126 | 40 |
| ConA | Kag30 | 48 | MO000057132 | PKCalpha(m) | 0.44032 | 0.018 | 1.5766 | 41 |
| ConA | Kag30 | 48 | MO000058081 | p65PAK(m) | 0.40489 | 0.012 | 1.5586 | 46 |
| ConA | Kag30 | 48 | MO000033754 | PAK1(m) | 0.61556 | 0.013 | 1.3476 | 48 |
| ConA | Kag30 | 48 | MO000037328 | dock9(m) | 0.3127 | 0.014 | 1.8276 | 49 |
| ConA | Kag30 | 48 | MO000032774 | RIP1(m) | 0.27809 | 0.006 | 2.044 | 50 |
| ConA | Kag30 | 48 | MO000094161 | Hsp90-alpha(m) | 0.32771 | 0.022 | 1.6745 | 52 |
| ConA | Kag30 | 48 | MO000130207 | Arhgef9(m) | 0.27808 | 0.006 | 2.0033 | 52 |
| ConA | Kag30 | 48 | MO000199377 | Fgd1(m) | 0.27809 | 0.006 | 1.9596 | 52 |
| ConA | Kag30 | 48 | MO000083943 | islet1(m) | 0.23233 | 0.017 | 3.3314 | 53 |
| ConA | Kag30 | 48 | MO000085313 | PP2ACalpha(m) | 0.46393 | 0.042 | 1.3212 | 53 |
| ConA | Kag30 | 48 | MO000133670 | ALK-1(m) | 0.31052 | 0.042 | 1.6951 | 54 |
| ConA | Kag30 | 48 | MO000041437 | dsRNA:TLR3:TRIF | 0.39732 | 0.029 | 1.5021 | 55 |
| ConA | Kag30 | 48 | MO000036576 | BCAR-3(m) | 0.27714 | 0.007 | 1.8009 | 58 |
| ConA | Kag30 | 48 | MO000043038 | Caspase-2(m) | 0.38345 | 0.025 | 1.4711 | 58 |
| ConA | Kag30 | 48 | MO000057082 | AKT-1(m) | 0.60714 | 0.01 | 1.2477 | 58 |
| ConA | Kag30 | 48 | MO000137535 | geft(m) | 0.27692 | 0.011 | 1.8359 | 58 |
| ConA | Kag30 | 48 | MO000162692 | PHLPP(m) | 0.4502 | 0.017 | 1.3024 | 58 |
| ConA | Kag30 | 48 | MO000087712 | Hsp70-2(m) | 0.218 | 0.002 | 4.1789 | 60 |
| ConA | Kag30 | 48 | MO000084306 | SRC-1-isoform1(m) | 0.2282 | 0.024 | 2.4235 | 61 |
| ConA | Kag30 | 48 | MO000085227 | brca1(m) | 0.31104 | 0.05 | 1.5997 | 61 |
| ConA | Kag30 | 48 | MO000033771 | GRP78(m) | 0.21793 | 0.003 | 3.947 | 62 |
| ConA | Kag30 | 48 | MO000045322 | RIP140(m) | 0.23208 | 0.014 | 2.1393 | 62 |
| ConA | Kag30 | 48 | MO000026727 | FXR(m) | 0.22009 | 0.034 | 2.7547 | 63 |
| ConA | Kag30 | 48 | MO000059185 | PDK1(m) | 0.46321 | 0.047 | 1.2413 | 63 |
| ConA | Kag30 | 48 | MO000035094 | cGKI(m) | 0.3629 | 0.047 | 1.415 | 65 |
| ConA | Kag30 | 48 | MO000020508 | HRS(m) | 0.35201 | 0.048 | 1.4328 | 67 |
| ConA | Kag30 | 48 | MO000021497 | VDR(m) | 0.2199 | 0.047 | 2.1617 | 67 |
| ConA | Kag30 | 48 | MO000164667 | G-beta-1(m):G-gamma-2(m) | 0.30217 | 0.029 | 1.5709 | 67 |
| ConA | Kag30 | 48 | MO000279390 | AKT(m){aceK} | 0.37772 | 0.04 | 1.3083 | 69 |
| ConA | Kag30 | 48 | MO000086837 | Hsp90-beta(m) | 0.32771 | 0.022 | 1.4622 | 70 |
| ConA | Kag30 | 48 | MO000037430 | GRIT(m) | 0.2761 | 0.02 | 1.6272 | 71 |
| ConA | Kag30 | 48 | MO000022224 | dsRNA | 0.26574 | 0.021 | 1.6579 | 72 |
| ConA | Kag30 | 48 | MO000034185 | Shc-1(m) | 0.2741 | 0.034 | 1.6234 | 74 |
| ConA | Kag30 | 48 | MO000036293 | MgcRacGAP(m) | 0.27622 | 0.024 | 1.5852 | 74 |
| ConA | Kag30 | 48 | MO000106362 | PKCepsilon(m) | 0.3995 | 0.048 | 1.2262 | 74 |
| ConA | Kag30 | 48 | MO000227486 | Bnip2(m) | 0.27699 | 0.041 | 1.5622 | 74 |
| ConA | Kag30 | 48 | MO000021505 | Grb-2(m) | 0.36092 | 0.04 | 1.2904 | 75 |
| ConA | Kag30 | 48 | MO000113198 | T6BP(m) | 0.36199 | 0.038 | 1.2861 | 75 |
| ConA | Kag30 | 48 | MO000038316 | LPS:lbp:CD14:TLR4:MD-2:TIRAP:IRAK-2 | 0.39732 | 0.029 | 1.1127 | 79 |
| ConA | Kag30 | 48 | MO000314597 | mcf2l(m) | 0.27621 | 0.023 | 1.5553 | 80 |
| ConA | Kag30 | 48 | MO000007998 | M-CSF-1-R(m) | 0.22873 | 0.026 | 1.596 | 83 |
| ConA | Kag30 | 48 | MO000018482 | ADRB2R(m) | 0.2273 | 0.034 | 1.602 | 84 |
| ConA | Kag30 | 48 | MO000090078 | proCaspase-9(m) | 0.33513 | 0.027 | 1.2438 | 85 |
| ConA | Kag30 | 48 | MO000035357 | RhoGDI-1(m) | 0.28842 | 0.02 | 1.2838 | 89 |
| ConA | Kag30 | 48 | MO000036447 | Tiam-1(m) | 0.27662 | 0.037 | 1.3312 | 90 |
| ConA | Kag30 | 48 | MO000120015 | TLR3(m) | 0.24161 | 0.048 | 1.4572 | 91 |
| ConA | Kag30 | 48 | MO000122151 | ECT2(m) | 0.27609 | 0.02 | 1.2556 | 101 |
| ConA | Kag30 | 48 | MO000032306 | SHIP-isoform1(m) | 0.2105 | 0.029 | 1.3905 | 107 |
| ConA | Kag30 | 48 | MO000032304 | SHIP-isoform3(m) | 0.2105 | 0.029 | 1.3894 | 109 |
| ConA | Kag30 | 48 | MO000038315 | LPS:lbp:CD14:TLR4:MD-2:TIRAP | 0.2299 | 0.028 | 1.2402 | 111 |
| ConA | Kag30 | 48 | MO000019073 | SLP-76(m) | 0.2278 | 0.049 | 1.2014 | 116 |
| ConA | Kag30 | 48 | MO000155959 | IL-17RA(m) | 0.21867 | 0.033 | 1.1802 | 121 |
| ConA + Poly I:C | Kag30 | 48 | MO000089823 | cIAP-2(m) | 0.40921 | 0.018 | 3.2646 | 41 |
| ConA + Poly I:C | Kag30 | 48 | MO000019485 | IFNalpha, IFNbeta:IFNAR1{pY}:Tyk2{pY}:IFNAR2c{pY}:Jak1{pY} | 0.40708 | 0.001 | 3.035 | 48 |
| ConA + Poly I:C | Kag30 | 48 | MO000059714 | ErbB3(m) | 0.41471 | 0.008 | 2.8119 | 49 |
| ConA + Poly I:C | Kag30 | 48 | MO000019336 | TBK1(m) | 0.55936 | 0.001 | 2.4178 | 59 |
| ConA + Poly I:C | Kag30 | 48 | MO000007348 | IL-2Rbeta(m) | 0.46195 | 0.003 | 2.5274 | 60 |
| ConA + Poly I:C | Kag30 | 48 | MO000079029 | Jak1(m) | 0.39289 | 0.002 | 2.9889 | 61 |
| ConA + Poly I:C | Kag30 | 48 | MO000059735 | ErbB4(m) | 0.41469 | 0.021 | 2.6177 | 68 |
| ConA + Poly I:C | Kag30 | 48 | MO000149650 | TBK1(m) | 0.36431 | 0 | 2.9011 | 70 |
| ConA + Poly I:C | Kag30 | 48 | MO000017252 | IL-10R | 0.35061 | 0 | 2.992 | 75 |
| ConA + Poly I:C | Kag30 | 48 | MO000019522 | IL-22R1:IL-10R2 | 0.35061 | 0 | 2.992 | 75 |
| ConA + Poly I:C | Kag30 | 48 | MO000021320 | InsR(m) | 0.46434 | 0.005 | 2.3482 | 75 |
| ConA + Poly I:C | Kag30 | 48 | MO000033240 | EpoR(m) | 0.46512 | 0.001 | 2.2942 | 80 |
| ConA + Poly I:C | Kag30 | 48 | MO000016669 | IFNRI | 0.34998 | 0.001 | 2.8622 | 81 |
| ConA + Poly I:C | Kag30 | 48 | MO000013127 | STAT5A(m) | 0.33105 | 0.01 | 2.9942 | 89 |
| ConA + Poly I:C | Kag30 | 48 | MO000013134 | STAT5B(m) | 0.32681 | 0.015 | 3.0609 | 90 |
| ConA + Poly I:C | Kag30 | 48 | MO000034664 | c-Cbl(m) | 0.44308 | 0.003 | 2.2361 | 94 |
| ConA + Poly I:C | Kag30 | 48 | MO000079158 | Syk(m) | 0.40688 | 0.022 | 2.388 | 94 |
| ConA + Poly I:C | Kag30 | 48 | MO000114529 | Src(m){pY} | 0.46513 | 0.007 | 2.1855 | 95 |
| ConA + Poly I:C | Kag30 | 48 | MO000017162 | c-Kit(m) | 0.51101 | 0.007 | 2.1534 | 96 |
| ConA + Poly I:C | Kag30 | 48 | MO000041182 | TRIF(m) | 0.38534 | 0.002 | 2.4277 | 98 |
| ConA + Poly I:C | Kag30 | 48 | MO000059450 | Jak2(m) | 0.47699 | 0.025 | 2.1066 | 104 |
| ConA + Poly I:C | Kag30 | 48 | MO000025777 | STAT3(m) | 0.34796 | 0.025 | 2.5647 | 105 |
| ConA + Poly I:C | Kag30 | 48 | MO000256766 | PDGFRalpha(m) | 0.42214 | 0.006 | 2.2053 | 105 |
| ConA + Poly I:C | Kag30 | 48 | MO000062597 | FCRG(m) | 0.3263 | 0.003 | 2.7422 | 106 |
| ConA + Poly I:C | Kag30 | 48 | MO000084048 | SHP1-isoform1(m) | 0.43748 | 0.029 | 2.1629 | 107 |
| ConA + Poly I:C | Kag30 | 48 | MO000017705 | FcepsilonRI | 0.33445 | 0.002 | 2.6314 | 108 |
| ConA + Poly I:C | Kag30 | 48 | MO000057014 | SHP2-isoform2(m) | 0.4133 | 0.004 | 2.2238 | 108 |
| ConA + Poly I:C | Kag30 | 48 | MO000043830 | PRLR(m) | 0.41373 | 0.005 | 2.2141 | 109 |
| ConA + Poly I:C | Kag30 | 48 | MO000017821 | Igalpha:Igbeta | 0.32292 | 0.002 | 2.7304 | 110 |
| ConA + Poly I:C | Kag30 | 48 | MO000018208 | FcgammaRI | 0.32308 | 0.002 | 2.7214 | 111 |
| ConA + Poly I:C | Kag30 | 48 | MO000166537 | FcgammaRIII(m) | 0.32235 | 0.003 | 2.7267 | 112 |
| ConA + Poly I:C | Kag30 | 48 | MO000022056 | Jak1(m) | 0.65126 | 0.002 | 1.929 | 115 |
| ConA + Poly I:C | Kag30 | 48 | MO000120015 | TLR3(m) | 0.28785 | 0 | 3.1758 | 118 |
| ConA + Poly I:C | Kag30 | 48 | MO000037686 | Bmx(m) | 0.4392 | 0.01 | 2.065 | 119 |
| ConA + Poly I:C | Kag30 | 48 | MO000006539 | Hck(m) | 0.57164 | 0.005 | 1.9632 | 120 |
| ConA + Poly I:C | Kag30 | 48 | MO000085462 | c-Cbl(m) | 0.33052 | 0.006 | 2.5211 | 120 |
| ConA + Poly I:C | Kag30 | 48 | MO000117511 | TC-PTPa(m) | 0.59113 | 0.002 | 1.9274 | 120 |
| ConA + Poly I:C | Kag30 | 48 | MO000120045 | UbcH7(m) | 0.44489 | 0.017 | 2.0493 | 120 |
| ConA + Poly I:C | Kag30 | 48 | MO000032972 | IRF-3(m) | 0.27704 | 0.012 | 3.3521 | 121 |
| ConA + Poly I:C | Kag30 | 48 | MO000037747 | LynA(m) | 0.41537 | 0.002 | 2.0848 | 121 |
| ConA + Poly I:C | Kag30 | 48 | MO000056839 | c-Kit(m) | 0.2997 | 0.003 | 2.7454 | 121 |
| ConA + Poly I:C | Kag30 | 48 | MO000022202 | IRAK-1(m) | 0.37065 | 0.034 | 2.2488 | 122 |
| ConA + Poly I:C | Kag30 | 48 | MO000032836 | IFNAR2(m) | 0.31905 | 0.003 | 2.6292 | 123 |
| ConA + Poly I:C | Kag30 | 48 | MO000032852 | OSMRbeta(m) | 0.31903 | 0.003 | 2.6295 | 123 |
| ConA + Poly I:C | Kag30 | 48 | MO000037746 | LynB(m) | 0.41537 | 0.002 | 2.0848 | 123 |
| ConA + Poly I:C | Kag30 | 48 | MO000059453 | Jak2(m){pY} | 0.34011 | 0.019 | 2.4176 | 126 |
| ConA + Poly I:C | Kag30 | 48 | MO000001937 | Btk(m) | 0.34399 | 0.024 | 2.4071 | 127 |
| ConA + Poly I:C | Kag30 | 48 | MO000033795 | Tyk2(m) | 0.52937 | 0.014 | 1.9045 | 130 |
| ConA + Poly I:C | Kag30 | 48 | MO000253996 | TBK1(m){pS172} | 0.2638 | 0.002 | 3.1007 | 130 |
| ConA + Poly I:C | Kag30 | 48 | MO000032013 | Jak2(m){p} | 0.32146 | 0.024 | 2.4941 | 132 |
| ConA + Poly I:C | Kag30 | 48 | MO000044233 | Jak2(m){pY1007} | 0.29584 | 0.007 | 2.6443 | 132 |
| ConA + Poly I:C | Kag30 | 48 | MO000089319 | Caspase(m) | 0.41604 | 0.019 | 2.0036 | 133 |
| ConA + Poly I:C | Kag30 | 48 | MO000022316 | PKCiota(m) | 0.40516 | 0.001 | 2.066 | 134 |
| ConA + Poly I:C | Kag30 | 48 | MO000038554 | Epo:(EpoR{pY})2:Lyn | 0.28453 | 0.007 | 2.7623 | 134 |
| ConA + Poly I:C | Kag30 | 48 | MO000021349 | RPTPalpha(m) | 0.46507 | 0.012 | 1.9256 | 135 |
| ConA + Poly I:C | Kag30 | 48 | MO000057591 | PLCgamma2(m) | 0.29295 | 0.006 | 2.6335 | 135 |
| ConA + Poly I:C | Kag30 | 48 | MO000089140 | gp130(m) | 0.31877 | 0.004 | 2.4925 | 136 |
| ConA + Poly I:C | Kag30 | 48 | MO000120165 | IRF-3(m) | 0.24639 | 0.016 | 3.33 | 139 |
| ConA + Poly I:C | Kag30 | 48 | MO000016687 | IFNRII | 0.3508 | 0.02 | 2.191 | 140 |
| ConA + Poly I:C | Kag30 | 48 | MO000043894 | GHR(m) | 0.35688 | 0.021 | 2.1587 | 140 |
| ConA + Poly I:C | Kag30 | 48 | MO000079450 | STAT5B(m) | 0.24801 | 0.012 | 3.2341 | 140 |
| ConA + Poly I:C | Kag30 | 48 | MO000056866 | EpoR-F(m) | 0.27963 | 0.006 | 2.7111 | 143 |
| ConA + Poly I:C | Kag30 | 48 | MO000037455 | Cas(m) | 0.35437 | 0.012 | 2.1171 | 146 |
| ConA + Poly I:C | Kag30 | 48 | MO000008302 | Syk(m) | 0.58166 | 0.008 | 1.8012 | 147 |
| ConA + Poly I:C | Kag30 | 48 | MO000018489 | AT1A(m) | 0.35128 | 0.025 | 2.1187 | 147 |
| ConA + Poly I:C | Kag30 | 48 | MO000084528 | STAT5A(m) | 0.24801 | 0.02 | 2.9351 | 148 |
| ConA + Poly I:C | Kag30 | 48 | MO000020140 | TCR(m) | 0.29996 | 0.009 | 2.4131 | 149 |
| ConA + Poly I:C | Kag30 | 48 | MO000020191 | SHP-2(m) | 0.56656 | 0.017 | 1.826 | 149 |
| ConA + Poly I:C | Kag30 | 48 | MO000025653 | IRF-4(m) | 0.25144 | 0.027 | 2.7904 | 149 |
| ConA + Poly I:C | Kag30 | 48 | MO000032784 | PLCgamma1(m) | 0.28988 | 0.008 | 2.5162 | 149 |
| ConA + Poly I:C | Kag30 | 48 | MO000120044 | UbcH7(m) | 0.3115 | 0.021 | 2.3863 | 150 |
| ConA + Poly I:C | Kag30 | 48 | MO000071130 | BGPI(m) | 0.32572 | 0.009 | 2.2849 | 151 |
| ConA + Poly I:C | Kag30 | 48 | MO000083014 | Hck-isoform1(m) | 0.31207 | 0.009 | 2.3114 | 153 |
| ConA + Poly I:C | Kag30 | 48 | MO000032890 | PTP-PEST(m) | 0.40904 | 0.009 | 1.9263 | 154 |
| ConA + Poly I:C | Kag30 | 48 | MO000057490 | InsR(m){pY} | 0.27922 | 0.002 | 2.5836 | 154 |
| ConA + Poly I:C | Kag30 | 48 | MO000156307 | FcepsilonRI beta(m) | 0.34068 | 0.014 | 2.1729 | 155 |
| ConA + Poly I:C | Kag30 | 48 | MO000032010 | H-Ras(m) | 0.42389 | 0.021 | 1.8625 | 159 |
| ConA + Poly I:C | Kag30 | 48 | MO000165399 | IKK-i(m){p} | 0.23631 | 0.007 | 2.8096 | 161 |
| ConA + Poly I:C | Kag30 | 48 | MO000042840 | ptpn21(m) | 0.40154 | 0.014 | 1.9041 | 164 |
| ConA + Poly I:C | Kag30 | 48 | MO000020142 | BCR(m) | 0.28978 | 0.006 | 2.3443 | 166 |
| ConA + Poly I:C | Kag30 | 48 | MO000057446 | fer(m) | 0.40072 | 0.027 | 1.9022 | 166 |
| ConA + Poly I:C | Kag30 | 48 | MO000020192 | SHP-1(m) | 0.5672 | 0.017 | 1.7599 | 167 |
| ConA + Poly I:C | Kag30 | 48 | MO000013131 | STAT6(m) | 0.24128 | 0.015 | 2.6503 | 169 |
| ConA + Poly I:C | Kag30 | 48 | MO000059631 | fgfr1(m) | 0.34528 | 0.032 | 2.0483 | 169 |
| ConA + Poly I:C | Kag30 | 48 | MO000057306 | Src(m) | 0.40258 | 0.018 | 1.877 | 171 |
| ConA + Poly I:C | Kag30 | 48 | MO000149349 | RNF126(m) | 0.29752 | 0.011 | 2.2295 | 172 |
| ConA + Poly I:C | Kag30 | 48 | MO000240859 | Rnf115(m) | 0.29753 | 0.011 | 2.2292 | 172 |
| ConA + Poly I:C | Kag30 | 48 | MO000038037 | nosip(m) | 0.24136 | 0.002 | 2.6185 | 174 |
| ConA + Poly I:C | Kag30 | 48 | MO000032786 | IRS-1(m) | 0.34595 | 0.024 | 1.9753 | 178 |
| ConA + Poly I:C | Kag30 | 48 | MO000034766 | HEF1(m) | 0.34723 | 0.02 | 1.9696 | 178 |
| ConA + Poly I:C | Kag30 | 48 | MO000059306 | Pyk2(m) | 0.35162 | 0.036 | 1.9084 | 178 |
| ConA + Poly I:C | Kag30 | 48 | MO000032104 | c-Kit(m){pY} | 0.26843 | 0.012 | 2.3061 | 182 |
| ConA + Poly I:C | Kag30 | 48 | MO000036267 | pld2(m) | 0.23473 | 0.003 | 2.5877 | 182 |
| ConA + Poly I:C | Kag30 | 48 | MO000058103 | CD45(m) | 0.50029 | 0.036 | 1.7148 | 184 |
| ConA + Poly I:C | Kag30 | 48 | MO000056794 | PTP1B(m) | 0.66273 | 0.006 | 1.618 | 185 |
| ConA + Poly I:C | Kag30 | 48 | MO000020959 | VEGFR-2(m) | 0.33179 | 0.028 | 1.9833 | 186 |
| ConA + Poly I:C | Kag30 | 48 | MO000042988 | Nrdp1(m) | 0.23569 | 0.002 | 2.5103 | 186 |
| ConA + Poly I:C | Kag30 | 48 | MO000103567 | E2-C(m) | 0.46313 | 0.029 | 1.7213 | 187 |
| ConA + Poly I:C | Kag30 | 48 | MO000007259 | IGF-2(m) | 0.29168 | 0.014 | 2.1554 | 189 |
| ConA + Poly I:C | Kag30 | 48 | MO000178618 | TSLP(m) | 0.2348 | 0.003 | 2.4336 | 191 |
| ConA + Poly I:C | Kag30 | 48 | MO000022052 | Jak2(m) | 0.66595 | 0.025 | 1.4637 | 192 |
| ConA + Poly I:C | Kag30 | 48 | MO000103665 | SLAM(m) | 0.29018 | 0.017 | 2.1497 | 192 |
| ConA + Poly I:C | Kag30 | 48 | MO000082357 | ErbB1(m) | 0.45018 | 0.045 | 1.7012 | 194 |
| ConA + Poly I:C | Kag30 | 48 | MO000023372 | SIRT1(m) | 0.74228 | 0.023 | 1.1124 | 196 |
| ConA + Poly I:C | Kag30 | 48 | MO000044588 | parkin:Cul-1:Fbw7 | 0.2495 | 0.009 | 2.2938 | 196 |
| ConA + Poly I:C | Kag30 | 48 | MO000008801 | Lyn(m) | 0.62949 | 0.01 | 1.3992 | 199 |
| ConA + Poly I:C | Kag30 | 48 | MO000013004 | Src(m) | 0.63449 | 0.041 | 1.2984 | 199 |
| ConA + Poly I:C | Kag30 | 48 | MO000022062 | Jak3(m) | 0.60163 | 0.024 | 1.4146 | 199 |
| ConA + Poly I:C | Kag30 | 48 | MO000022549 | IKK-beta(m) | 0.50562 | 0.024 | 1.5939 | 199 |
| ConA + Poly I:C | Kag30 | 48 | MO000010973 | PDGFRbeta(m) | 0.40476 | 0.028 | 1.7589 | 202 |
| ConA + Poly I:C | Kag30 | 48 | MO000056751 | PTP-PEST(m) | 0.35893 | 0.024 | 1.7868 | 203 |
| ConA + Poly I:C | Kag30 | 48 | MO000038316 | LPS:lbp:CD14:TLR4:MD-2:TIRAP:IRAK-2 | 0.38908 | 0.017 | 1.7738 | 204 |
| ConA + Poly I:C | Kag30 | 48 | MO000041437 | dsRNA:TLR3:TRIF | 0.38907 | 0.017 | 1.7751 | 204 |
| ConA + Poly I:C | Kag30 | 48 | MO000126162 | Cd72(m) | 0.29545 | 0.018 | 2.0243 | 204 |
| ConA + Poly I:C | Kag30 | 48 | MO000034686 | CAP(m) | 0.22827 | 0.005 | 2.4228 | 205 |
| ConA + Poly I:C | Kag30 | 48 | MO000086151 | traf6-isoform1(m) | 0.36125 | 0.032 | 1.7759 | 209 |
| ConA + Poly I:C | Kag30 | 48 | MO000007687 | IRF-1(m) | 0.20524 | 0.041 | 2.7512 | 210 |
| ConA + Poly I:C | Kag30 | 48 | MO000032508 | SOCS-3(m) | 0.37403 | 0.031 | 1.7579 | 213 |
| ConA + Poly I:C | Kag30 | 48 | MO000032835 | IL-7(m) | 0.25364 | 0.017 | 2.1119 | 214 |
| ConA + Poly I:C | Kag30 | 48 | MO000088836 | NF-kappaB1(m) | 0.28861 | 0.049 | 2.0057 | 214 |
| ConA + Poly I:C | Kag30 | 48 | MO000022017 | APS(m) | 0.23644 | 0.008 | 2.2152 | 216 |
| ConA + Poly I:C | Kag30 | 48 | MO000084046 | CD19(m) | 0.3684 | 0.027 | 1.7496 | 217 |
| ConA + Poly I:C | Kag30 | 48 | MO000056726 | FRS2alpha(m) | 0.25258 | 0.021 | 2.0634 | 223 |
| ConA + Poly I:C | Kag30 | 48 | MO000114548 | FRS3(m) | 0.25258 | 0.021 | 2.061 | 223 |
| ConA + Poly I:C | Kag30 | 48 | MO000186097 | RPTPalpha(m){pS204} | 0.23325 | 0.009 | 2.2122 | 223 |
| ConA + Poly I:C | Kag30 | 48 | MO000038722 | PDGFBB:(PDGFRbeta{pY})2 | 0.22671 | 0.005 | 2.2869 | 224 |
| ConA + Poly I:C | Kag30 | 48 | MO000032785 | Yes(m) | 0.30577 | 0.032 | 1.8744 | 225 |
| ConA + Poly I:C | Kag30 | 48 | MO000004576 | EphA4(m) | 0.41281 | 0.044 | 1.5071 | 227 |
| ConA + Poly I:C | Kag30 | 48 | MO000038315 | LPS:lbp:CD14:TLR4:MD-2:TIRAP | 0.22622 | 0.005 | 2.2811 | 227 |
| ConA + Poly I:C | Kag30 | 48 | MO000032271 | SOCS-1(m) | 0.40017 | 0.045 | 1.6446 | 229 |
| ConA + Poly I:C | Kag30 | 48 | MO000039080 | IRAK-4(m) | 0.25012 | 0.021 | 2.0458 | 230 |
| ConA + Poly I:C | Kag30 | 48 | MO000007513 | IFNgamma(m) | 0.30112 | 0.047 | 1.8594 | 232 |
| ConA + Poly I:C | Kag30 | 48 | MO000121660 | alpha IIb-integrin(m):beta3-integrin(m) | 0.31642 | 0.03 | 1.8274 | 232 |
| ConA + Poly I:C | Kag30 | 48 | MO000041137 | TRAM(m) | 0.20659 | 0.001 | 2.4527 | 233 |
| ConA + Poly I:C | Kag30 | 48 | MO000107884 | Cbl-b(m) | 0.28948 | 0.029 | 1.8901 | 233 |
| ConA + Poly I:C | Kag30 | 48 | MO000239966 | Ptpn23(m) | 0.40062 | 0.036 | 1.5799 | 233 |
| ConA + Poly I:C | Kag30 | 48 | MO000082402 | MEKK1-xbb2(m) | 0.34504 | 0.02 | 1.7551 | 234 |
| ConA + Poly I:C | Kag30 | 48 | MO000005218 | flt3(m) | 0.21155 | 0.027 | 2.4093 | 235 |
| ConA + Poly I:C | Kag30 | 48 | MO000032886 | C3G(m) | 0.24807 | 0.019 | 2.0173 | 235 |
| ConA + Poly I:C | Kag30 | 48 | MO000236690 | Mad2l1bp(m) | 0.25624 | 0.018 | 1.9641 | 237 |
| ConA + Poly I:C | Kag30 | 48 | MO000075867 | MEKK1(m) | 0.34504 | 0.023 | 1.7391 | 238 |
| ConA + Poly I:C | Kag30 | 48 | MO000003800 | IL-5Rbeta(m) | 0.28209 | 0.029 | 1.8883 | 239 |
| ConA + Poly I:C | Kag30 | 48 | MO000057793 | Lck(m) | 0.34977 | 0.027 | 1.7003 | 239 |
| ConA + Poly I:C | Kag30 | 48 | MO000007186 | IRF-8(m) | 0.21197 | 0.043 | 2.3383 | 240 |
| ConA + Poly I:C | Kag30 | 48 | MO000119664 | IRAK-4(m) | 0.24577 | 0.029 | 2.0002 | 242 |
| ConA + Poly I:C | Kag30 | 48 | MO000032840 | IRS-2(m) | 0.3291 | 0.045 | 1.718 | 250 |
| ConA + Poly I:C | Kag30 | 48 | MO000054385 | Ret(m) | 0.27574 | 0.046 | 1.8623 | 253 |
| ConA + Poly I:C | Kag30 | 48 | MO000035461 | Syk(m){pY} | 0.20144 | 0.015 | 2.3062 | 254 |
| ConA + Poly I:C | Kag30 | 48 | MO000150828 | PKD1(m) | 0.32205 | 0.043 | 1.7276 | 254 |
| ConA + Poly I:C | Kag30 | 48 | MO000165441 | Syk(m){p} | 0.20256 | 0.004 | 2.3032 | 255 |
| ConA + Poly I:C | Kag30 | 48 | MO000088390 | IRF-8(m) | 0.20383 | 0.036 | 2.2925 | 257 |
| ConA + Poly I:C | Kag30 | 48 | MO000167478 | DHHC18(m) | 0.34557 | 0.047 | 1.5558 | 259 |
| ConA + Poly I:C | Kag30 | 48 | MO000254011 | Grb-14(m) | 0.21429 | 0.011 | 2.1048 | 260 |
| ConA + Poly I:C | Kag30 | 48 | MO000032829 | IFNalpha1(m) | 0.28717 | 0.037 | 1.761 | 271 |
| ConA + Poly I:C | Kag30 | 48 | MO000019478 | Grb10(m) | 0.21395 | 0.016 | 2.0234 | 274 |
| ConA + Poly I:C | Kag30 | 48 | MO000110074 | prmt2(m) | 0.21771 | 0.035 | 1.9955 | 274 |
| ConA + Poly I:C | Kag30 | 48 | MO000037344 | IRSp53(m) | 0.29623 | 0.041 | 1.6849 | 276 |
| ConA + Poly I:C | Kag30 | 48 | MO000110075 | prmt2(m) | 0.21771 | 0.035 | 1.9931 | 276 |
| ConA + Poly I:C | Kag30 | 48 | MO000057352 | PTP1B(m){pY} | 0.21427 | 0.012 | 1.9695 | 282 |
| ConA + Poly I:C | Kag30 | 48 | MO000165629 | IKK-beta(m){pS177}{pS181} | 0.23301 | 0.02 | 1.8594 | 282 |
| ConA + Poly I:C | Kag30 | 48 | MO000089405 | Jak2(m){pY} | 0.22239 | 0.021 | 1.8974 | 286 |
| ConA + Poly I:C | Kag30 | 48 | MO000042687 | CIN85(m) | 0.22567 | 0.025 | 1.8675 | 291 |
| ConA + Poly I:C | Kag30 | 48 | MO000001761 | Blk(m) | 0.25779 | 0.044 | 1.7192 | 292 |
| ConA + Poly I:C | Kag30 | 48 | MO000022224 | dsRNA | 0.243 | 0.023 | 1.7705 | 295 |
| ConA + Poly I:C | Kag30 | 48 | MO000151911 | IKK-alpha(m){p} | 0.2789 | 0.05 | 1.6556 | 297 |
| ConA + Poly I:C | Kag30 | 48 | MO000038127 | Src(m){pY424} | 0.2133 | 0.022 | 1.8882 | 299 |
| ConA + Poly I:C | Kag30 | 48 | MO000170572 | IKK-beta(m){pS} | 0.22494 | 0.026 | 1.8314 | 300 |
| ConA + Poly I:C | Kag30 | 48 | MO000115111 | Src(m){pY527} | 0.2133 | 0.022 | 1.8882 | 301 |
| ConA + Poly I:C | Kag30 | 48 | MO000034105 | fer-isoform1(m) | 0.22222 | 0.018 | 1.8578 | 302 |
| ConA + Poly I:C | Kag30 | 48 | MO000034106 | fer-isoform3(m) | 0.22223 | 0.018 | 1.8576 | 302 |
| ConA + Poly I:C | Kag30 | 48 | MO000120961 | E1:Ubc7{ub(1)} | 0.22905 | 0.027 | 1.7763 | 302 |
| ConA + Poly I:C | Kag30 | 48 | MO000130073 | PKCiota(m) | 0.21419 | 0.011 | 1.8689 | 302 |
| ConA + Poly I:C | Kag30 | 48 | MO000160215 | E1:Ubc5A{ub(1)} | 0.22905 | 0.027 | 1.7763 | 302 |
| ConA + Poly I:C | Kag30 | 48 | MO000188322 | E1{ub(1)}:Ubc13:Mms2 | 0.22905 | 0.027 | 1.7763 | 302 |
| ConA + Poly I:C | Kag30 | 48 | MO000200433 | RPTPkappa(m) | 0.26513 | 0.042 | 1.6461 | 302 |
| ConA + Poly I:C | Kag30 | 48 | MO000060232 | Src(m){p} | 0.2133 | 0.022 | 1.8882 | 303 |
| ConA + Poly I:C | Kag30 | 48 | MO000119264 | E1:UbcH7{ub(1)} | 0.22905 | 0.027 | 1.7767 | 303 |
| ConA + Poly I:C | Kag30 | 48 | MO000120050 | UBE2G2(m) | 0.22888 | 0.026 | 1.7771 | 303 |
| ConA + Poly I:C | Kag30 | 48 | MO000187670 | E1{ub(1)}:Ubc13:Uev1 | 0.22905 | 0.027 | 1.7759 | 306 |
| ConA + Poly I:C | Kag30 | 48 | MO000036516 | Rac1(m):GTP | 0.2521 | 0.031 | 1.6619 | 307 |
| ConA + Poly I:C | Kag30 | 48 | MO000010243 | leptin(m) | 0.24515 | 0.04 | 1.6726 | 314 |
| ConA + Poly I:C | Kag30 | 48 | MO000084051 | Lyn(m){pY397} | 0.21225 | 0.024 | 1.7961 | 321 |
| ConA + Poly I:C | Kag30 | 48 | MO000084053 | Lyn(m){pY397}{pY508} | 0.21225 | 0.024 | 1.7976 | 323 |
| ConA + Poly I:C | Kag30 | 48 | MO000105496 | Lyn(m){pY} | 0.21225 | 0.024 | 1.7953 | 324 |
| ConA + Poly I:C | Kag30 | 48 | MO000197545 | Lyn(m){pY508} | 0.21225 | 0.024 | 1.7953 | 324 |
| ConA + Poly I:C | Kag30 | 48 | MO000020892 | Par3(m) | 0.22543 | 0.022 | 1.7431 | 325 |
| ConA + Poly I:C | Kag30 | 48 | MO000033731 | Sprouty2(m) | 0.23079 | 0.041 | 1.6789 | 325 |
| ConA + Poly I:C | Kag30 | 48 | MO000019393 | IL-1RAcP(m) | 0.21239 | 0.039 | 1.7663 | 334 |
| ConA + Poly I:C | Kag30 | 48 | MO000032843 | CrkL(m) | 0.22362 | 0.029 | 1.713 | 334 |
| ConA + Poly I:C | Kag30 | 48 | MO000097965 | trkA(m) | 0.23126 | 0.047 | 1.5524 | 339 |
| ConA + Poly I:C | Kag30 | 48 | MO000078708 | ZAP-70(m){pY} | 0.21933 | 0.05 | 1.4454 | 361 |
| ConA + Poly I:C | Kag30 | 48 | MO000077967 | Sh2d3c(m){myr}{pY} | 0.20747 | 0.034 | 1.6449 | 366 |
| ConA + Poly I:C | Kag30 | 48 | MO000229786 | RPTP-beta(m) | 0.20407 | 0.04 | 1.665 | 368 |
| ConA + Poly I:C | Kag30 | 48 | MO000285936 | Ptpn14(m) | 0.20737 | 0.038 | 1.6398 | 369 |
| ConA + Poly I:C | Kag30 | 48 | MO000285937 | Ptpn14(m) | 0.20734 | 0.04 | 1.6185 | 371 |
| ConA + Poly I:C | Kag30 | 48 | MO000007998 | M-CSF-1-R(m) | 0.20833 | 0.047 | 1.504 | 376 |
| ConA + Poly I:C | Kag30 | 48 | MO000244783 | Trim72(m) | 0.20499 | 0.043 | 1.5609 | 378 |
| ConA + Poly I:C | Kag30 | 96 | MO000026727 | FXR(m) | 0.48937 | 0.005 | 3.9011 | 2 |
| ConA + Poly I:C | Kag30 | 96 | MO000044514 | CHIP(m) | 0.49856 | 0.023 | 1.6625 | 10 |
| ConA + Poly I:C | Kag30 | 96 | MO000056800 | RXR-alpha(m) | 0.3201 | 0.018 | 3.6409 | 21 |
| ConA + Poly I:C | Kag30 | 96 | MO000025590 | RXR-alpha(m) | 0.31998 | 0.025 | 3.2657 | 25 |
| ConA + Poly I:C | Kag30 | 96 | MO000032382 | KSR(m) | 0.4048 | 0.011 | 1.3962 | 26 |
| ConA + Poly I:C | Kag30 | 96 | MO000485088 | MEKK3(m){p} | 0.34096 | 0.02 | 1.5373 | 31 |
| ConA + Poly I:C | Kag30 | 96 | MO000480373 | MEKK2(m){p} | 0.34096 | 0.02 | 1.5188 | 33 |
| ConA + Poly I:C | Kag30 | 96 | MO000058137 | MEKK2(m) | 0.48161 | 0.008 | 1.2497 | 35 |
| ConA + Poly I:C | Kag30 | 96 | MO000101268 | cholate | 0.26711 | 0.006 | 3.5357 | 35 |
| ConA + Poly I:C | Kag30 | 96 | MO000114689 | chenodeoxycholic acid | 0.26711 | 0.006 | 3.634 | 35 |
| ConA + Poly I:C | Kag30 | 96 | MO000121303 | Raf(m){p} | 0.40412 | 0.012 | 1.2897 | 36 |
| ConA + Poly I:C | Kag30 | 96 | MO000105093 | YY1(m) | 0.27859 | 0.023 | 2.0883 | 37 |
| ConA + Poly I:C | Kag30 | 96 | MO000128521 | MEK(m){p} | 0.49386 | 0.013 | 1.1569 | 38 |
| ConA + Poly I:C | Kag30 | 96 | MO000082097 | ERK5-isoform1(m) | 0.46282 | 0.031 | 1.1959 | 39 |
| ConA + Poly I:C | Kag30 | 96 | MO000043173 | Rac1:GTP:(MLK3{p})2 | 0.31769 | 0.038 | 1.3994 | 41 |
| ConA + Poly I:C | Kag30 | 96 | MO000243058 | Stk25(m) | 0.27942 | 0.01 | 1.5861 | 41 |
| ConA + Poly I:C | Kag30 | 96 | MO000038590 | Rac1:GTP:MEKK4 | 0.34975 | 0.049 | 1.2998 | 42 |
| ConA + Poly I:C | Kag30 | 96 | MO000085073 | Mapk(m){p} | 0.24323 | 0.007 | 1.7489 | 42 |
| ConA + Poly I:C | Kag30 | 96 | MO000019403 | SITPEC(m) | 0.29101 | 0.041 | 1.5024 | 43 |
| ConA + Poly I:C | Kag30 | 96 | MO000084411 | RSK2(m) | 0.37058 | 0.049 | 1.2663 | 43 |
| ConA + Poly I:C | Kag30 | 96 | MO000120701 | p62-isoform1(m) | 0.24723 | 0.027 | 1.6944 | 43 |
| ConA + Poly I:C | Kag30 | 96 | MO000022221 | MEKK1(m){p} | 0.35951 | 0.046 | 1.2709 | 44 |
| ConA + Poly I:C | Kag30 | 96 | MO000038048 | PAK4(m) | 0.31666 | 0.045 | 1.3821 | 44 |
| ConA + Poly I:C | Kag30 | 96 | MO000038759 | GCK:(MEKK1{pT})2 | 0.35008 | 0.031 | 1.276 | 44 |
| ConA + Poly I:C | Kag30 | 96 | MO000102207 | ERK2(m){p} | 0.44221 | 0.011 | 1.1153 | 44 |
| ConA + Poly I:C | Kag30 | 96 | MO000019483 | map4k4(m) | 0.29088 | 0.04 | 1.4908 | 45 |
| ConA + Poly I:C | Kag30 | 96 | MO000120857 | CD154(m) | 0.23862 | 0.014 | 1.7386 | 45 |
| ConA + Poly I:C | Kag30 | 96 | MO000084682 | MEKK4-A(m) | 0.35955 | 0.039 | 1.2353 | 46 |
| ConA + Poly I:C | Kag30 | 96 | MO000021497 | VDR(m) | 0.21793 | 0.033 | 2.4842 | 47 |
| ConA + Poly I:C | Kag30 | 96 | MO000032847 | JIP1(m) | 0.40153 | 0.04 | 1.1497 | 47 |
| ConA + Poly I:C | Kag30 | 96 | MO000038806 | PKCdelta:DAG:Raf-1 | 0.40413 | 0.012 | 1.1126 | 47 |
| ConA + Poly I:C | Kag30 | 96 | MO000086659 | PKCbeta(m) | 0.31056 | 0.037 | 1.3185 | 48 |
| ConA + Poly I:C | Kag30 | 96 | MO000120192 | SIGIRR(m) | 0.23862 | 0.014 | 1.5656 | 48 |
| ConA + Poly I:C | Kag30 | 96 | MO000038749 | (TRAF2)2:(ASK1{p})2 | 0.34975 | 0.042 | 1.2147 | 51 |
| ConA + Poly I:C | Kag30 | 96 | MO000107964 | MEK2(m) | 0.3953 | 0.036 | 1.108 | 51 |
| ConA + Poly I:C | Kag30 | 96 | MO000032857 | MEKK4(m) | 0.37108 | 0.037 | 1.0811 | 53 |
| ConA + Poly I:C | Kag30 | 96 | MO000130373 | Phb(m) | 0.23826 | 0.023 | 1.4435 | 56 |
| ConA + Poly I:C | Kag30 | 96 | MO000198693 | periostin(m) | 0.20954 | 0.019 | 1.4539 | 61 |
| ConA + Poly I:C | Kag30 | 96 | MO000178435 | DAG:PKCdelta(m) | 0.21985 | 0.01 | 1.3699 | 62 |
| ConA + Poly I:C | Kag30 | 96 | MO000255868 | PKCbeta-Beta-II(m) | 0.31056 | 0.037 | 1.1733 | 62 |
| ConA + Poly I:C | Kag30 | 96 | MO000033460 | JIP1-isoform1(m) | 0.22232 | 0.028 | 1.2968 | 66 |
| ConA + Poly I:C | Kag30 | 96 | MO000057223 | Ras(m):GTP | 0.21984 | 0.01 | 1.309 | 66 |
| ConA + Poly I:C | Kag30 | 96 | MO000075867 | MEKK1(m) | 0.31629 | 0.048 | 1.0461 | 67 |
| ConA + Poly I:C | Kag30 | 96 | MO000158713 | KSR-isoform1(m) | 0.20976 | 0.01 | 1.3159 | 67 |
| ConA + Poly I:C | Kag30 | 96 | MO000032874 | beta-arrestin2(m) | 0.30187 | 0.05 | 1.0226 | 71 |
| ConA | Kag100 | 48 | MO000089823 | cIAP-2(m) | 0.38899 | 0.02 | 3.3388 | 15 |
| ConA | Kag100 | 48 | MO000019336 | TBK1(m) | 0.49584 | 0.001 | 2.4772 | 22 |
| ConA | Kag100 | 48 | MO000041182 | TRIF(m) | 0.37336 | 0 | 2.9436 | 26 |
| ConA | Kag100 | 48 | MO000120045 | UbcH7(m) | 0.42345 | 0.008 | 2.4226 | 29 |
| ConA | Kag100 | 48 | MO000086151 | traf6-isoform1(m) | 0.36798 | 0.007 | 2.5562 | 35 |
| ConA | Kag100 | 48 | MO000038316 | LPS:lbp:CD14:TLR4:MD-2:TIRAP:IRAK-2 | 0.37922 | 0.001 | 2.3793 | 39 |
| ConA | Kag100 | 48 | MO000041437 | dsRNA:TLR3:TRIF | 0.37922 | 0.001 | 2.3801 | 39 |
| ConA | Kag100 | 48 | MO000200602 | march5(m) | 0.34407 | 0.003 | 2.3948 | 46 |
| ConA | Kag100 | 48 | MO000232618 | beta-TrCP1-isoform1(m) | 0.30599 | 0.035 | 2.9403 | 46 |
| ConA | Kag100 | 48 | MO000234738 | Irak2(m) | 0.29459 | 0.005 | 2.894 | 50 |
| ConA | Kag100 | 48 | MO000128620 | EAC(m) | 0.40566 | 0.005 | 2.2179 | 53 |
| ConA | Kag100 | 48 | MO000019402 | traf6(m) | 0.55256 | 0.007 | 2.1117 | 54 |
| ConA | Kag100 | 48 | MO000098007 | Nod2(m) | 0.33523 | 0.005 | 2.3561 | 54 |
| ConA | Kag100 | 48 | MO000120015 | TLR3(m) | 0.25289 | 0 | 3.6034 | 55 |
| ConA | Kag100 | 48 | MO000016606 | IKK-gamma(m) | 0.34293 | 0.01 | 2.3123 | 56 |
| ConA | Kag100 | 48 | MO000021719 | A20(m) | 0.36345 | 0.007 | 2.255 | 58 |
| ConA | Kag100 | 48 | MO000059659 | IRAK-1(m){p} | 0.33252 | 0.005 | 2.3241 | 58 |
| ConA | Kag100 | 48 | MO000024810 | ER-alpha(m) | 0.2516 | 0.025 | 3.3038 | 59 |
| ConA | Kag100 | 48 | MO000032775 | p75NTR(m) | 0.28813 | 0.008 | 2.5832 | 59 |
| ConA | Kag100 | 48 | MO000149650 | TBK1(m) | 0.27174 | 0.004 | 2.7124 | 61 |
| ConA | Kag100 | 48 | MO000022316 | PKCiota(m) | 0.37922 | 0.001 | 2.1813 | 62 |
| ConA | Kag100 | 48 | MO000103567 | E2-C(m) | 0.42278 | 0.009 | 2.1223 | 62 |
| ConA | Kag100 | 48 | MO000121034 | CIKS(m) | 0.33243 | 0.005 | 2.3101 | 62 |
| ConA | Kag100 | 48 | MO000245279 | Usp25(m) | 0.33749 | 0.005 | 2.2966 | 62 |
| ConA | Kag100 | 48 | MO000036067 | TRAF3(m) | 0.27722 | 0.006 | 2.5046 | 65 |
| ConA | Kag100 | 48 | MO000113198 | T6BP(m) | 0.347 | 0.005 | 2.1898 | 67 |
| ConA | Kag100 | 48 | MO000200601 | march5(m) | 0.34406 | 0.007 | 2.2155 | 68 |
| ConA | Kag100 | 48 | MO000120702 | p62(m) | 0.3246 | 0.004 | 2.2545 | 71 |
| ConA | Kag100 | 48 | MO000245273 | Usp22(m) | 0.48144 | 0.026 | 1.731 | 74 |
| ConA | Kag100 | 48 | MO000336179 | TAK1(m){ub} | 0.2921 | 0.007 | 2.3092 | 74 |
| ConA | Kag100 | 48 | MO000017453 | TAK1(m) | 0.47266 | 0.008 | 1.8235 | 75 |
| ConA | Kag100 | 48 | MO000044588 | parkin:Cul-1:Fbw7 | 0.22418 | 0 | 2.9448 | 75 |
| ConA | Kag100 | 48 | MO000119981 | NIK(m) | 0.47538 | 0.01 | 1.7467 | 75 |
| ConA | Kag100 | 48 | MO000026727 | FXR(m) | 0.22836 | 0.012 | 2.7923 | 77 |
| ConA | Kag100 | 48 | MO000121772 | A20(m) | 0.3664 | 0.01 | 2.0881 | 77 |
| ConA | Kag100 | 48 | MO000057082 | AKT-1(m) | 0.47796 | 0.043 | 1.7168 | 80 |
| ConA | Kag100 | 48 | MO000088390 | IRF-8(m) | 0.21298 | 0.029 | 3.155 | 80 |
| ConA | Kag100 | 48 | MO000019250 | RIP(m) | 0.22816 | 0.007 | 2.5251 | 83 |
| ConA | Kag100 | 48 | MO000082402 | MEKK1-xbb2(m) | 0.31867 | 0.001 | 2.1678 | 83 |
| ConA | Kag100 | 48 | MO000023330 | E1(m) | 0.74619 | 0.037 | 1.0811 | 85 |
| ConA | Kag100 | 48 | MO000033754 | PAK1(m) | 0.51863 | 0.044 | 1.5306 | 85 |
| ConA | Kag100 | 48 | MO000056936 | SIRT1-isoform1(m) | 0.63129 | 0.046 | 1.1589 | 85 |
| ConA | Kag100 | 48 | MO000075867 | MEKK1(m) | 0.31867 | 0.002 | 2.1497 | 85 |
| ConA | Kag100 | 48 | MO000081385 | TAK1(m) | 0.27209 | 0.008 | 2.2488 | 85 |
| ConA | Kag100 | 48 | MO000022549 | IKK-beta(m) | 0.47399 | 0.019 | 1.6322 | 86 |
| ConA | Kag100 | 48 | MO000007186 | IRF-8(m) | 0.21283 | 0.038 | 2.8277 | 87 |
| ConA | Kag100 | 48 | MO000059191 | AKT(m){p} | 0.36502 | 0.009 | 1.8392 | 87 |
| ConA | Kag100 | 48 | MO000086646 | NF-kappaB2(m) | 0.20575 | 0.022 | 3.14 | 87 |
| ConA | Kag100 | 48 | MO000170572 | IKK-beta(m){pS} | 0.21318 | 0.002 | 2.6566 | 87 |
| ConA | Kag100 | 48 | MO000002525 | CD28(m) | 0.23418 | 0.003 | 2.3523 | 88 |
| ConA | Kag100 | 48 | MO000020145 | TRAF2(m) | 0.30461 | 0.019 | 2.1315 | 89 |
| ConA | Kag100 | 48 | MO000165629 | IKK-beta(m){pS177}{pS181} | 0.21436 | 0.001 | 2.5212 | 90 |
| ConA | Kag100 | 48 | MO000032858 | TRAF4(m) | 0.30263 | 0.012 | 2.1259 | 91 |
| ConA | Kag100 | 48 | MO000032572 | Rac1(m) | 0.38477 | 0.019 | 1.6406 | 92 |
| ConA | Kag100 | 48 | MO000020508 | HRS(m) | 0.32964 | 0.012 | 2.018 | 93 |
| ConA | Kag100 | 48 | MO000036021 | EDAR(m) | 0.23814 | 0.009 | 2.3089 | 94 |
| ConA | Kag100 | 48 | MO000165056 | cIAP-1(m) | 0.33175 | 0.033 | 1.9013 | 94 |
| ConA | Kag100 | 48 | MO000161481 | Cytochrome b-558:p22phox:p40phox{p}:p67phox:Rac1:GTP:JFC1:PtdIns(3,4)P2:PA:p47phox | 0.3847 | 0.021 | 1.6168 | 95 |
| ConA | Kag100 | 48 | MO000180126 | PP2Cbeta(m) | 0.41495 | 0.032 | 1.4768 | 95 |
| ConA | Kag100 | 48 | MO000037328 | dock9(m) | 0.24564 | 0.008 | 2.245 | 96 |
| ConA | Kag100 | 48 | MO000044385 | parkin(m) | 0.29111 | 0.022 | 2.1007 | 98 |
| ConA | Kag100 | 48 | MO000041135 | tab2(m) | 0.24062 | 0.005 | 2.2261 | 99 |
| ConA | Kag100 | 48 | MO000119721 | tab3(m) | 0.24066 | 0.005 | 2.2251 | 99 |
| ConA | Kag100 | 48 | MO000120168 | Ubc13(m) | 0.33918 | 0.035 | 1.7303 | 99 |
| ConA | Kag100 | 48 | MO000236690 | Mad2l1bp(m) | 0.21443 | 0.003 | 2.3186 | 100 |
| ConA | Kag100 | 48 | MO000122582 | IKK-i(m) | 0.29445 | 0.023 | 1.8891 | 104 |
| ConA | Kag100 | 48 | MO000020892 | Par3(m) | 0.20718 | 0.004 | 2.3515 | 107 |
| ConA | Kag100 | 48 | MO000095704 | TRIF(m) | 0.23392 | 0.006 | 2.1686 | 110 |
| ConA | Kag100 | 48 | MO000161841 | ube2h(m) | 0.28657 | 0.028 | 1.8621 | 110 |
| ConA | Kag100 | 48 | MO000019958 | Cul-1(m) | 0.25331 | 0.028 | 2.0299 | 111 |
| ConA | Kag100 | 48 | MO000039080 | IRAK-4(m) | 0.21126 | 0.012 | 2.2619 | 112 |
| ConA | Kag100 | 48 | MO000127428 | Rock-1(m) | 0.25447 | 0.023 | 1.8785 | 115 |
| ConA | Kag100 | 48 | MO000019364 | RelA-p65(m) | 0.22521 | 0.034 | 2.1398 | 117 |
| ConA | Kag100 | 48 | MO000120044 | UbcH7(m) | 0.25023 | 0.039 | 1.938 | 117 |
| ConA | Kag100 | 48 | MO000019483 | map4k4(m) | 0.25539 | 0.024 | 1.7957 | 119 |
| ConA | Kag100 | 48 | MO000086968 | HPK1(m) | 0.26769 | 0.038 | 1.7183 | 124 |
| ConA | Kag100 | 48 | MO000285932 | Cezanne(m) | 0.20964 | 0.015 | 2.1811 | 125 |
| ConA | Kag100 | 48 | MO000019403 | SITPEC(m) | 0.25262 | 0.029 | 1.7259 | 128 |
| ConA | Kag100 | 48 | MO000022224 | dsRNA | 0.21315 | 0.009 | 2.0983 | 130 |
| ConA | Kag100 | 48 | MO000151911 | IKK-alpha(m){p} | 0.23998 | 0.045 | 1.7268 | 133 |
| ConA | Kag100 | 48 | MO000042734 | TACE(m) | 0.21847 | 0.041 | 1.8314 | 137 |
| ConA | Kag100 | 48 | MO000119664 | IRAK-4(m) | 0.20486 | 0.023 | 2.0526 | 140 |
| ConA | Kag100 | 48 | MO000020525 | Bax(m) | 0.22862 | 0.037 | 1.681 | 142 |
| ConA | Kag100 | 48 | MO000103566 | E2-C(m) | 0.22295 | 0.036 | 1.7138 | 146 |
| ConA | Kag100 | 48 | MO000032807 | BDNF(m) | 0.21028 | 0.046 | 1.5359 | 161 |
| ConA | Kag100 | 48 | MO000137535 | geft(m) | 0.20337 | 0.05 | 1.5558 | 165 |
| ConA + Poly I:C | Kag100 | 96 | MO000026727 | FXR(m) | 0.50026 | 0.004 | 3.6928 | 0 |
| ConA + Poly I:C | Kag100 | 96 | MO000018273 | NLK(m) | 0.39914 | 0.026 | 2.665 | 18 |
| ConA + Poly I:C | Kag100 | 96 | MO000056800 | RXR-alpha(m) | 0.33079 | 0.022 | 3.406 | 22 |
| ConA + Poly I:C | Kag100 | 96 | MO000024810 | ER-alpha(m) | 0.35701 | 0.009 | 2.7498 | 26 |
| ConA + Poly I:C | Kag100 | 96 | MO000035474 | Cdc42(m) | 0.48005 | 0.003 | 1.5856 | 27 |
| ConA + Poly I:C | Kag100 | 96 | MO000025590 | RXR-alpha(m) | 0.33053 | 0.041 | 2.9878 | 28 |
| ConA + Poly I:C | Kag100 | 96 | MO000041437 | dsRNA:TLR3:TRIF | 0.42796 | 0.02 | 1.6002 | 29 |
| ConA + Poly I:C | Kag100 | 96 | MO000045693 | Hsp90-beta(m) | 0.39131 | 0.044 | 1.6305 | 30 |
| ConA + Poly I:C | Kag100 | 96 | MO000044514 | CHIP(m) | 0.478 | 0.042 | 1.5432 | 31 |
| ConA + Poly I:C | Kag100 | 96 | MO000020508 | HRS(m) | 0.40861 | 0.027 | 1.5694 | 33 |
| ConA + Poly I:C | Kag100 | 96 | MO000101268 | cholate | 0.2915 | 0.006 | 3.2468 | 33 |
| ConA + Poly I:C | Kag100 | 96 | MO000114689 | chenodeoxycholic acid | 0.29149 | 0.006 | 3.3325 | 33 |
| ConA + Poly I:C | Kag100 | 96 | MO000128620 | EAC(m) | 0.44308 | 0.038 | 1.5389 | 33 |
| ConA + Poly I:C | Kag100 | 96 | MO000028472 | SRC-1(m) | 0.3197 | 0.038 | 2.2047 | 37 |
| ConA + Poly I:C | Kag100 | 96 | MO000314409 | hipk2-isoform4(m){p} | 0.28033 | 0.028 | 3.0194 | 38 |
| ConA + Poly I:C | Kag100 | 96 | MO000121034 | CIKS(m) | 0.38671 | 0.03 | 1.5578 | 39 |
| ConA + Poly I:C | Kag100 | 96 | MO000188175 | NLK(m) | 0.28029 | 0.03 | 2.8252 | 41 |
| ConA + Poly I:C | Kag100 | 96 | MO000099002 | hipk2-isoform4(m) | 0.49128 | 0.045 | 1.212 | 42 |
| ConA + Poly I:C | Kag100 | 96 | MO000022224 | dsRNA | 0.29538 | 0.015 | 1.8436 | 44 |
| ConA + Poly I:C | Kag100 | 96 | MO000037328 | dock9(m) | 0.30979 | 0.022 | 1.6873 | 44 |
| ConA + Poly I:C | Kag100 | 96 | MO000094162 | Hsp90-alpha(m) | 0.3911 | 0.046 | 1.4507 | 45 |
| ConA + Poly I:C | Kag100 | 96 | MO000037506 | gamma-secretase(m) | 0.31125 | 0.046 | 1.6175 | 46 |
| ConA + Poly I:C | Kag100 | 96 | MO000059659 | IRAK-1(m){p} | 0.38672 | 0.032 | 1.4139 | 47 |
| ConA + Poly I:C | Kag100 | 96 | MO000087712 | Hsp70-2(m) | 0.2035 | 0.005 | 3.6125 | 47 |
| ConA + Poly I:C | Kag100 | 96 | MO000245279 | Usp25(m) | 0.38607 | 0.034 | 1.4878 | 48 |
| ConA + Poly I:C | Kag100 | 96 | MO000200602 | march5(m) | 0.3867 | 0.039 | 1.4099 | 50 |
| ConA + Poly I:C | Kag100 | 96 | MO000105093 | YY1(m) | 0.27039 | 0.03 | 1.8066 | 51 |
| ConA + Poly I:C | Kag100 | 96 | MO000113198 | T6BP(m) | 0.42582 | 0.011 | 1.1556 | 51 |
| ConA + Poly I:C | Kag100 | 96 | MO000033771 | GRP78(m) | 0.20344 | 0.015 | 3.1309 | 52 |
| ConA + Poly I:C | Kag100 | 96 | MO000200601 | march5(m) | 0.38669 | 0.045 | 1.368 | 52 |
| ConA + Poly I:C | Kag100 | 96 | MO000081385 | TAK1(m) | 0.33354 | 0.031 | 1.4744 | 53 |
| ConA + Poly I:C | Kag100 | 96 | MO000032774 | RIP1(m) | 0.26678 | 0.008 | 1.7472 | 54 |
| ConA + Poly I:C | Kag100 | 96 | MO000083943 | islet1(m) | 0.22021 | 0.031 | 2.63 | 55 |
| ConA + Poly I:C | Kag100 | 96 | MO000130207 | Arhgef9(m) | 0.26677 | 0.008 | 1.6733 | 57 |
| ConA + Poly I:C | Kag100 | 96 | MO000021497 | VDR(m) | 0.21026 | 0.044 | 2.2914 | 58 |
| ConA + Poly I:C | Kag100 | 96 | MO000041511 | traf6{ub}:TAK1{p}:TAB1{p}:tab2:PKR | 0.35772 | 0.05 | 1.1971 | 58 |
| ConA + Poly I:C | Kag100 | 96 | MO000045322 | RIP140(m) | 0.22991 | 0.019 | 2.0961 | 58 |
| ConA + Poly I:C | Kag100 | 96 | MO000119721 | tab3(m) | 0.30622 | 0.029 | 1.4905 | 58 |
| ConA + Poly I:C | Kag100 | 96 | MO000199377 | Fgd1(m) | 0.26678 | 0.008 | 1.6016 | 58 |
| ConA + Poly I:C | Kag100 | 96 | MO000084306 | SRC-1-isoform1(m) | 0.20135 | 0.025 | 2.537 | 60 |
| ConA + Poly I:C | Kag100 | 96 | MO000234738 | Irak2(m) | 0.32305 | 0.039 | 1.2858 | 62 |
| ConA + Poly I:C | Kag100 | 96 | MO000336179 | TAK1(m){ub} | 0.34361 | 0.047 | 1.1772 | 62 |
| ConA + Poly I:C | Kag100 | 96 | MO000137535 | geft(m) | 0.26563 | 0.042 | 1.5163 | 70 |
| ConA + Poly I:C | Kag100 | 96 | MO000120015 | TLR3(m) | 0.25422 | 0.041 | 1.5278 | 71 |
| ConA + Poly I:C | Kag100 | 96 | MO000036447 | Tiam-1(m) | 0.28879 | 0.029 | 1.1796 | 74 |
| ConA + Poly I:C | Kag100 | 96 | MO000041135 | tab2(m) | 0.30616 | 0.029 | 1.0926 | 75 |
| ConA + Poly I:C | Kag100 | 96 | MO000037430 | GRIT(m) | 0.26484 | 0.05 | 1.3596 | 79 |
| ConA + Poly I:C | Kag100 | 96 | MO000036576 | BCAR-3(m) | 0.26585 | 0.01 | 1.1476 | 84 |
| ConA + Poly I:C | Kag100 | 96 | MO000038315 | LPS:lbp:CD14:TLR4:MD-2:TIRAP | 0.24501 | 0.025 | 1.1382 | 89 |
| ConA | Kag300 | 48 | MO000089823 | cIAP-2(m) | 0.38743 | 0.03 | 3.1649 | 24 |
| ConA | Kag300 | 48 | MO000019336 | TBK1(m) | 0.456 | 0.001 | 2.4573 | 31 |
| ConA | Kag300 | 48 | MO000041182 | TRIF(m) | 0.37727 | 0.001 | 2.8139 | 33 |
| ConA | Kag300 | 48 | MO000017453 | TAK1(m) | 0.48148 | 0.005 | 2.1315 | 44 |
| ConA | Kag300 | 48 | MO000022316 | PKCiota(m) | 0.39928 | 0 | 2.3475 | 46 |
| ConA | Kag300 | 48 | MO000245273 | Usp22(m) | 0.49823 | 0.011 | 1.9727 | 48 |
| ConA | Kag300 | 48 | MO000232618 | beta-TrCP1-isoform1(m) | 0.30788 | 0.032 | 3.0752 | 51 |
| ConA | Kag300 | 48 | MO000059191 | AKT(m){p} | 0.39662 | 0.002 | 2.1813 | 54 |
| ConA | Kag300 | 48 | MO000120702 | p62(m) | 0.351 | 0.004 | 2.3658 | 55 |
| ConA | Kag300 | 48 | MO000036067 | TRAF3(m) | 0.28667 | 0.001 | 2.8848 | 59 |
| ConA | Kag300 | 48 | MO000119981 | NIK(m) | 0.48733 | 0.004 | 1.8609 | 60 |
| ConA | Kag300 | 48 | MO000336179 | TAK1(m){ub} | 0.30753 | 0.001 | 2.6327 | 60 |
| ConA | Kag300 | 48 | MO000024810 | ER-alpha(m) | 0.26782 | 0.03 | 3.3716 | 61 |
| ConA | Kag300 | 48 | MO000019402 | traf6(m) | 0.51414 | 0.042 | 1.7709 | 63 |
| ConA | Kag300 | 48 | MO000081385 | TAK1(m) | 0.28804 | 0.002 | 2.6272 | 63 |
| ConA | Kag300 | 48 | MO000120045 | UbcH7(m) | 0.40943 | 0.03 | 1.8841 | 65 |
| ConA | Kag300 | 48 | MO000082402 | MEKK1-xbb2(m) | 0.33129 | 0.007 | 2.2428 | 67 |
| ConA | Kag300 | 48 | MO000088836 | NF-kappaB1(m) | 0.28327 | 0.029 | 2.5384 | 67 |
| ConA | Kag300 | 48 | MO000009087 | MEKK1(m) | 0.51486 | 0.018 | 1.7048 | 68 |
| ConA | Kag300 | 48 | MO000020145 | TRAF2(m) | 0.31893 | 0.006 | 2.3643 | 69 |
| ConA | Kag300 | 48 | MO000022549 | IKK-beta(m) | 0.49427 | 0.01 | 1.7246 | 69 |
| ConA | Kag300 | 48 | MO000075867 | MEKK1(m) | 0.33129 | 0.007 | 2.2221 | 69 |
| ConA | Kag300 | 48 | MO000032858 | TRAF4(m) | 0.3166 | 0.007 | 2.3656 | 70 |
| ConA | Kag300 | 48 | MO000149650 | TBK1(m) | 0.26464 | 0.001 | 2.6345 | 72 |
| ConA | Kag300 | 48 | MO000165056 | cIAP-1(m) | 0.34164 | 0.026 | 2.0489 | 72 |
| ConA | Kag300 | 48 | MO000180126 | PP2Cbeta(m) | 0.44849 | 0.017 | 1.7293 | 73 |
| ConA | Kag300 | 48 | MO000120015 | TLR3(m) | 0.24224 | 0 | 3.2085 | 74 |
| ConA | Kag300 | 48 | MO000016606 | IKK-gamma(m) | 0.32346 | 0.023 | 2.1712 | 76 |
| ConA | Kag300 | 48 | MO000128620 | EAC(m) | 0.39506 | 0.022 | 1.8509 | 76 |
| ConA | Kag300 | 48 | MO000038316 | LPS:lbp:CD14:TLR4:MD-2:TIRAP:IRAK-2 | 0.37149 | 0.011 | 1.8717 | 77 |
| ConA | Kag300 | 48 | MO000041437 | dsRNA:TLR3:TRIF | 0.37148 | 0.011 | 1.8719 | 77 |
| ConA | Kag300 | 48 | MO000041135 | tab2(m) | 0.25862 | 0.001 | 2.5384 | 78 |
| ConA | Kag300 | 48 | MO000119721 | tab3(m) | 0.25866 | 0.001 | 2.5374 | 78 |
| ConA | Kag300 | 48 | MO000019250 | RIP(m) | 0.24646 | 0.001 | 2.8614 | 79 |
| ConA | Kag300 | 48 | MO000146524 | pkmyt1(m) | 0.43121 | 0.033 | 1.694 | 80 |
| ConA | Kag300 | 48 | MO000103567 | E2-C(m) | 0.42001 | 0.041 | 1.7007 | 82 |
| ConA | Kag300 | 48 | MO000007186 | IRF-8(m) | 0.22224 | 0.04 | 2.9247 | 83 |
| ConA | Kag300 | 48 | MO000020508 | HRS(m) | 0.33154 | 0.025 | 1.8795 | 84 |
| ConA | Kag300 | 48 | MO000020892 | Par3(m) | 0.23519 | 0 | 2.7409 | 85 |
| ConA | Kag300 | 48 | MO000045239 | Aos1(m):SAE2(m) | 0.54429 | 0.02 | 1.4151 | 85 |
| ConA | Kag300 | 48 | MO000127428 | Rock-1(m) | 0.28304 | 0.009 | 2.2745 | 85 |
| ConA | Kag300 | 48 | MO000127914 | Cdc25C(m) | 0.43113 | 0.037 | 1.6424 | 85 |
| ConA | Kag300 | 48 | MO000088390 | IRF-8(m) | 0.21959 | 0.035 | 3.0574 | 86 |
| ConA | Kag300 | 48 | MO000087611 | Cdk1(m) | 0.43236 | 0.05 | 1.5839 | 88 |
| ConA | Kag300 | 48 | MO000095704 | TRIF(m) | 0.25744 | 0.003 | 2.4388 | 88 |
| ConA | Kag300 | 48 | MO000025566 | GATA-2(m) | 0.20204 | 0.024 | 3.6236 | 89 |
| ConA | Kag300 | 48 | MO000002525 | CD28(m) | 0.24655 | 0.005 | 2.4836 | 90 |
| ConA | Kag300 | 48 | MO000022110 | IKK-alpha(m) | 0.66235 | 0.028 | 1.1342 | 90 |
| ConA | Kag300 | 48 | MO000036656 | RSK1(m) | 0.46723 | 0.034 | 1.4691 | 90 |
| ConA | Kag300 | 48 | MO000036021 | EDAR(m) | 0.25007 | 0.001 | 2.4139 | 92 |
| ConA | Kag300 | 48 | MO000086151 | traf6-isoform1(m) | 0.33746 | 0.04 | 1.8118 | 92 |
| ConA | Kag300 | 48 | MO000234738 | Irak2(m) | 0.28309 | 0.017 | 2.112 | 92 |
| ConA | Kag300 | 48 | MO000020525 | Bax(m) | 0.25795 | 0.008 | 2.3176 | 93 |
| ConA | Kag300 | 48 | MO000086968 | HPK1(m) | 0.28685 | 0.02 | 2.0466 | 93 |
| ConA | Kag300 | 48 | MO000021719 | A20(m) | 0.34045 | 0.04 | 1.7313 | 94 |
| ConA | Kag300 | 48 | MO000087375 | PIAS3-isoform2(m) | 0.32833 | 0.038 | 1.8485 | 94 |
| ConA | Kag300 | 48 | MO000200602 | march5(m) | 0.32535 | 0.025 | 1.8532 | 94 |
| ConA | Kag300 | 48 | MO000044385 | parkin(m) | 0.294 | 0.041 | 1.9163 | 95 |
| ConA | Kag300 | 48 | MO000086101 | PIASy(m) | 0.42206 | 0.042 | 1.5128 | 95 |
| ConA | Kag300 | 48 | MO000019483 | map4k4(m) | 0.27599 | 0.016 | 2.0784 | 96 |
| ConA | Kag300 | 48 | MO000285932 | Cezanne(m) | 0.22007 | 0.005 | 2.5356 | 97 |
| ConA | Kag300 | 48 | MO000044588 | parkin:Cul-1:Fbw7 | 0.2155 | 0.001 | 2.5357 | 99 |
| ConA | Kag300 | 48 | MO000021312 | PP2Cbeta1(m) | 0.40386 | 0.033 | 1.4439 | 100 |
| ConA | Kag300 | 48 | MO000019403 | SITPEC(m) | 0.27354 | 0.016 | 2.0393 | 102 |
| ConA | Kag300 | 48 | MO000098007 | Nod2(m) | 0.31798 | 0.035 | 1.7542 | 104 |
| ConA | Kag300 | 48 | MO000026727 | FXR(m) | 0.20573 | 0.025 | 2.4954 | 105 |
| ConA | Kag300 | 48 | MO000057073 | GSK3beta(m) | 0.38085 | 0.04 | 1.4014 | 107 |
| ConA | Kag300 | 48 | MO000059659 | IRAK-1(m){p} | 0.31557 | 0.045 | 1.7097 | 110 |
| ConA | Kag300 | 48 | MO000084602 | IKK-gamma(m) | 0.2012 | 0.003 | 2.4629 | 111 |
| ConA | Kag300 | 48 | MO000245279 | Usp25(m) | 0.31998 | 0.04 | 1.6885 | 111 |
| ConA | Kag300 | 48 | MO000057087 | AKT-1(m){pS473} | 0.37851 | 0.043 | 1.358 | 112 |
| ConA | Kag300 | 48 | MO000151911 | IKK-alpha(m){p} | 0.2546 | 0.037 | 1.9045 | 114 |
| ConA | Kag300 | 48 | MO000032775 | p75NTR(m) | 0.27479 | 0.037 | 1.8472 | 115 |
| ConA | Kag300 | 48 | MO000121034 | CIKS(m) | 0.3155 | 0.046 | 1.6917 | 115 |
| ConA | Kag300 | 48 | MO000128191 | AKT-1(m){pS473} | 0.35991 | 0.05 | 1.3808 | 115 |
| ConA | Kag300 | 48 | MO000167333 | Laforin(m) | 0.35992 | 0.05 | 1.3806 | 115 |
| ConA | Kag300 | 48 | MO000339319 | AKT-1(m){p} | 0.35991 | 0.05 | 1.3808 | 115 |
| ConA | Kag300 | 48 | MO000037328 | dock9(m) | 0.24845 | 0.029 | 1.9065 | 116 |
| ConA | Kag300 | 48 | MO000113198 | T6BP(m) | 0.32792 | 0.046 | 1.5692 | 118 |
| ConA | Kag300 | 48 | MO000165629 | IKK-beta(m){pS177}{pS181} | 0.21053 | 0.009 | 2.205 | 119 |
| ConA | Kag300 | 48 | MO000170572 | IKK-beta(m){pS} | 0.20533 | 0.012 | 2.2559 | 119 |
| ConA | Kag300 | 48 | MO000236690 | Mad2l1bp(m) | 0.21989 | 0.016 | 2.0994 | 119 |
| ConA | Kag300 | 48 | MO000019958 | Cul-1(m) | 0.25364 | 0.038 | 1.8636 | 120 |
| ConA | Kag300 | 48 | MO000019364 | RelA-p65(m) | 0.21527 | 0.045 | 1.9624 | 128 |
| ConA | Kag300 | 48 | MO000122582 | IKK-i(m) | 0.27365 | 0.049 | 1.598 | 134 |
| ConA | Kag300 | 48 | MO000043647 | Tome-1(m) | 0.22593 | 0.037 | 1.6133 | 150 |
| ConA | Kag300 | 48 | MO000004929 | Fas(m) | 0.22183 | 0.038 | 1.6419 | 151 |
| ConA | Kag300 | 48 | MO000337385 | Cdk1(m){p} | 0.23657 | 0.04 | 1.5804 | 151 |
| ConA | Kag300 | 48 | MO000199484 | Cdk1(m){pY15} | 0.23657 | 0.04 | 1.5804 | 153 |
| ConA | Kag300 | 48 | MO000171736 | AKT-2(m){pS} | 0.21275 | 0.032 | 1.6772 | 155 |
| ConA | Kag300 | 48 | MO000022224 | dsRNA | 0.20571 | 0.044 | 1.6125 | 162 |
| ConA | Lent | 48 | MO000044514 | CHIP(m) | 0.48634 | 0.017 | 1.9211 | 21 |
| ConA | Lent | 48 | MO000232618 | beta-TrCP1-isoform1(m) | 0.36245 | 0.021 | 2.4593 | 24 |
| ConA | Lent | 48 | MO000245279 | Usp25(m) | 0.41407 | 0.021 | 1.9285 | 26 |
| ConA | Lent | 48 | MO000024810 | ER-alpha(m) | 0.32592 | 0.017 | 2.8919 | 27 |
| ConA | Lent | 48 | MO000019957 | Skp1(m) | 0.3496 | 0.013 | 2.3612 | 28 |
| ConA | Lent | 48 | MO000234738 | Irak2(m) | 0.36133 | 0.026 | 2.1143 | 28 |
| ConA | Lent | 48 | MO000098007 | Nod2(m) | 0.40921 | 0.025 | 1.9151 | 29 |
| ConA | Lent | 48 | MO000113198 | T6BP(m) | 0.41875 | 0.026 | 1.8443 | 29 |
| ConA | Lent | 48 | MO000032775 | p75NTR(m) | 0.36083 | 0.024 | 2.1022 | 30 |
| ConA | Lent | 48 | MO000086151 | traf6-isoform1(m) | 0.41961 | 0.025 | 1.8409 | 30 |
| ConA | Lent | 48 | MO000059659 | IRAK-1(m){p} | 0.40388 | 0.032 | 1.8423 | 33 |
| ConA | Lent | 48 | MO000057082 | AKT-1(m) | 0.58665 | 0.024 | 1.672 | 34 |
| ConA | Lent | 48 | MO000059185 | PDK1(m) | 0.51984 | 0.019 | 1.7484 | 34 |
| ConA | Lent | 48 | MO000019402 | traf6(m) | 0.59212 | 0.041 | 1.6142 | 37 |
| ConA | Lent | 48 | MO000121034 | CIKS(m) | 0.40382 | 0.032 | 1.8177 | 38 |
| ConA | Lent | 48 | MO000245273 | Usp22(m) | 0.57521 | 0.026 | 1.6147 | 38 |
| ConA | Lent | 48 | MO000058349 | C/EBPalpha(m) | 0.31895 | 0.049 | 1.8904 | 40 |
| ConA | Lent | 48 | MO000099002 | hipk2-isoform4(m) | 0.49504 | 0.014 | 1.6417 | 41 |
| ConA | Lent | 48 | MO000098006 | Nod2(m) | 0.26938 | 0.018 | 2.0466 | 42 |
| ConA | Lent | 48 | MO000200602 | march5(m) | 0.40387 | 0.039 | 1.7655 | 45 |
| ConA | Lent | 48 | MO000025588 | RAR-gamma(m) | 0.22408 | 0.043 | 2.2761 | 48 |
| ConA | Lent | 48 | MO000056936 | SIRT1-isoform1(m) | 0.7269 | 0.015 | 1.1363 | 49 |
| ConA | Lent | 48 | MO000058424 | Nrf2(m) | 0.20277 | 0.02 | 4.6969 | 49 |
| ConA | Lent | 48 | MO000072832 | CHIP(m) | 0.38944 | 0.05 | 1.7518 | 49 |
| ConA | Lent | 48 | MO000080266 | ER-beta(m) | 0.21455 | 0.015 | 3.2873 | 49 |
| ConA | Lent | 48 | MO000119664 | IRAK-4(m) | 0.27903 | 0.041 | 1.834 | 49 |
| ConA | Lent | 48 | MO000021455 | AR(m) | 0.25005 | 0.025 | 1.9551 | 50 |
| ConA | Lent | 48 | MO000155959 | IL-17RA(m) | 0.2478 | 0.017 | 1.9767 | 51 |
| ConA | Lent | 48 | MO000180126 | PP2Cbeta(m) | 0.50078 | 0.031 | 1.4518 | 51 |
| ConA | Lent | 48 | MO000021312 | PP2Cbeta1(m) | 0.47726 | 0.038 | 1.4931 | 52 |
| ConA | Lent | 48 | MO000009417 | MKK6(m) | 0.50186 | 0.046 | 1.2333 | 53 |
| ConA | Lent | 48 | MO000034493 | PRK2(m) | 0.48726 | 0.05 | 1.4187 | 55 |
| ConA | Lent | 48 | MO000079423 | GSK3alpha(m) | 0.4304 | 0.05 | 1.4838 | 55 |
| ConA | Lent | 48 | MO000039080 | IRAK-4(m) | 0.2792 | 0.041 | 1.7662 | 56 |
| ConA | Lent | 48 | MO000162692 | PHLPP(m) | 0.47901 | 0.035 | 1.4441 | 56 |
| ConA | Lent | 48 | MO000032774 | RIP1(m) | 0.26253 | 0.017 | 1.8086 | 57 |
| ConA | Lent | 48 | MO000130207 | Arhgef9(m) | 0.26252 | 0.017 | 1.8089 | 57 |
| ConA | Lent | 48 | MO000199377 | Fgd1(m) | 0.26253 | 0.017 | 1.8086 | 57 |
| ConA | Lent | 48 | MO000022530 | TIRAP(m) | 0.29693 | 0.041 | 1.5757 | 66 |
| ConA | Lent | 48 | MO000022141 | traf6(m){ub{K63}(n)} | 0.22079 | 0.025 | 1.7856 | 67 |
| ConA | Lent | 48 | MO000134786 | ANG-1(m) | 0.25569 | 0.015 | 1.7554 | 67 |
| ConA | Lent | 48 | MO000128025 | traf6(m){ub}n | 0.22075 | 0.025 | 1.7829 | 69 |
| ConA | Lent | 48 | MO000022224 | dsRNA | 0.27357 | 0.041 | 1.5419 | 71 |
| ConA | Lent | 48 | MO000035357 | RhoGDI-1(m) | 0.28542 | 0.05 | 1.4906 | 71 |
| ConA | Lent | 48 | MO000036576 | BCAR-3(m) | 0.26158 | 0.029 | 1.6451 | 71 |
| ConA | Lent | 48 | MO000037328 | dock9(m) | 0.30865 | 0.045 | 1.4793 | 71 |
| ConA | Lent | 48 | MO000120886 | traf6(m){ub} | 0.22075 | 0.025 | 1.7829 | 71 |
| ConA | Lent | 48 | MO000032819 | NGF(m) | 0.24967 | 0.038 | 1.7379 | 72 |
| ConA | Lent | 48 | MO000137535 | geft(m) | 0.27311 | 0.043 | 1.5059 | 73 |
| ConA | Lent | 48 | MO000338496 | AKT-1(m){sumo} | 0.21495 | 0.04 | 1.5766 | 84 |
| ConA | Lent | 48 | MO000089823 | cIAP-2(m) | 0.40801 | 0.004 | 3.2224 | 19 |
| ConA | Lent | 48 | MO000149650 | TBK1(m) | 0.40356 | 0 | 2.8016 | 39 |
| ConA | Lent | 48 | MO000036067 | TRAF3(m) | 0.3693 | 0 | 2.999 | 43 |
| ConA | Lent | 48 | MO000128620 | EAC(m) | 0.48442 | 0.001 | 2.559 | 50 |
| ConA | Lent | 48 | MO000200602 | march5(m) | 0.36339 | 0 | 2.8121 | 56 |
| ConA | Lent | 48 | MO000021719 | A20(m) | 0.3841 | 0 | 2.695 | 58 |
| ConA | Lent | 48 | MO000285932 | Cezanne(m) | 0.36867 | 0.001 | 2.7773 | 58 |
| ConA | Lent | 48 | MO000016606 | IKK-gamma(m) | 0.43533 | 0.001 | 2.5264 | 59 |
| ConA | Lent | 48 | MO000121772 | A20(m) | 0.38379 | 0 | 2.6633 | 60 |
| ConA | Lent | 48 | MO000098007 | Nod2(m) | 0.34763 | 0 | 2.8164 | 61 |
| ConA | Lent | 48 | MO000245279 | Usp25(m) | 0.3525 | 0 | 2.7827 | 63 |
| ConA | Lent | 48 | MO000120015 | TLR3(m) | 0.29081 | 0 | 3.9096 | 68 |
| ConA | Lent | 48 | MO000234738 | Irak2(m) | 0.30083 | 0 | 3.099 | 70 |
| ConA | Lent | 48 | MO000020145 | TRAF2(m) | 0.35467 | 0 | 2.6556 | 72 |
| ConA | Lent | 48 | MO000059659 | IRAK-1(m){p} | 0.34229 | 0 | 2.7187 | 72 |
| ConA | Lent | 48 | MO000253996 | TBK1(m){pS172} | 0.29135 | 0.001 | 3.2024 | 72 |
| ConA | Lent | 48 | MO000200601 | march5(m) | 0.36338 | 0.001 | 2.6272 | 73 |
| ConA | Lent | 48 | MO000036021 | EDAR(m) | 0.34263 | 0 | 2.6952 | 74 |
| ConA | Lent | 48 | MO000086151 | traf6-isoform1(m) | 0.36628 | 0.007 | 2.6021 | 74 |
| ConA | Lent | 48 | MO000121034 | CIKS(m) | 0.34217 | 0 | 2.7105 | 74 |
| ConA | Lent | 48 | MO000081647 | IFNgamma(m) | 0.35443 | 0.008 | 2.6174 | 76 |
| ConA | Lent | 48 | MO000019373 | RelB(m) | 0.27516 | 0.018 | 3.3116 | 77 |
| ConA | Lent | 48 | MO000041182 | TRIF(m) | 0.43779 | 0 | 2.3411 | 77 |
| ConA | Lent | 48 | MO000019336 | TBK1(m) | 0.59247 | 0 | 2.2457 | 78 |
| ConA | Lent | 48 | MO000120045 | UbcH7(m) | 0.4306 | 0.007 | 2.3312 | 80 |
| ConA | Lent | 48 | MO000032775 | p75NTR(m) | 0.29394 | 0 | 2.9066 | 82 |
| ConA | Lent | 48 | MO000165056 | cIAP-1(m) | 0.40037 | 0.005 | 2.3671 | 84 |
| ConA | Lent | 48 | MO000081385 | TAK1(m) | 0.31437 | 0 | 2.6993 | 85 |
| ConA | Lent | 48 | MO000084602 | IKK-gamma(m) | 0.28865 | 0 | 2.8867 | 87 |
| ConA | Lent | 48 | MO000038316 | LPS:lbp:CD14:TLR4:MD-2:TIRAP:IRAK-2 | 0.37212 | 0.001 | 2.4343 | 88 |
| ConA | Lent | 48 | MO000041437 | dsRNA:TLR3:TRIF | 0.37212 | 0.001 | 2.4361 | 88 |
| ConA | Lent | 48 | MO000086646 | NF-kappaB2(m) | 0.25543 | 0.013 | 4.0346 | 89 |
| ConA | Lent | 48 | MO000165629 | IKK-beta(m){pS177}{pS181} | 0.26345 | 0 | 3.1579 | 91 |
| ConA | Lent | 48 | MO000170572 | IKK-beta(m){pS} | 0.26392 | 0 | 3.094 | 92 |
| ConA | Lent | 48 | MO000022316 | PKCiota(m) | 0.42764 | 0 | 2.2377 | 93 |
| ConA | Lent | 48 | MO000142292 | SIR2L6(m) | 0.32999 | 0.013 | 2.5064 | 94 |
| ConA | Lent | 48 | MO000019402 | traf6(m) | 0.52234 | 0.015 | 1.9105 | 95 |
| ConA | Lent | 48 | MO000086680 | TNF-alpha(m) | 0.36902 | 0.011 | 2.3437 | 96 |
| ConA | Lent | 48 | MO000336179 | TAK1(m){ub} | 0.3273 | 0 | 2.5036 | 96 |
| ConA | Lent | 48 | MO000142293 | SIR2L6(m) | 0.26074 | 0.001 | 3.0967 | 97 |
| ConA | Lent | 48 | MO000120165 | IRF-3(m) | 0.23547 | 0.017 | 3.2699 | 101 |
| ConA | Lent | 48 | MO000127428 | Rock-1(m) | 0.3083 | 0.003 | 2.5597 | 101 |
| ConA | Lent | 48 | MO000044385 | parkin(m) | 0.3128 | 0.004 | 2.5004 | 105 |
| ConA | Lent | 48 | MO000113198 | T6BP(m) | 0.34201 | 0 | 2.3876 | 107 |
| ConA | Lent | 48 | MO000119981 | NIK(m) | 0.5589 | 0.003 | 1.7326 | 107 |
| ConA | Lent | 48 | MO000022549 | IKK-beta(m) | 0.51932 | 0.01 | 1.7539 | 108 |
| ConA | Lent | 48 | MO000079630 | bcl10(m) | 0.23442 | 0 | 3.1203 | 108 |
| ConA | Lent | 48 | MO000120168 | Ubc13(m) | 0.35 | 0.011 | 2.3276 | 108 |
| ConA | Lent | 48 | MO000180126 | PP2Cbeta(m) | 0.46283 | 0.001 | 1.761 | 111 |
| ConA | Lent | 48 | MO000201247 | sharpin-isoform1(m):HOIP-isoform1(m) | 0.26261 | 0 | 2.7837 | 111 |
| ConA | Lent | 48 | MO000120169 | Ubc13(m) | 0.43806 | 0.013 | 1.766 | 112 |
| ConA | Lent | 48 | MO000122582 | IKK-i(m) | 0.37292 | 0.009 | 2.0906 | 112 |
| ConA | Lent | 48 | MO000017453 | TAK1(m) | 0.48195 | 0 | 1.723 | 113 |
| ConA | Lent | 48 | MO000019250 | RIP(m) | 0.26272 | 0.003 | 2.7359 | 114 |
| ConA | Lent | 48 | MO000041137 | TRAM(m) | 0.23941 | 0 | 2.901 | 115 |
| ConA | Lent | 48 | MO000059191 | AKT(m){p} | 0.38233 | 0.004 | 1.996 | 115 |
| ConA | Lent | 48 | MO000151911 | IKK-alpha(m){p} | 0.30391 | 0.009 | 2.4667 | 115 |
| ConA | Lent | 48 | MO000032858 | TRAF4(m) | 0.33097 | 0.001 | 2.305 | 117 |
| ConA | Lent | 48 | MO000130073 | PKCiota(m) | 0.25324 | 0 | 2.7762 | 118 |
| ConA | Lent | 48 | MO000025653 | IRF-4(m) | 0.23155 | 0.018 | 2.9746 | 119 |
| ConA | Lent | 48 | MO000068857 | Omi(m) | 0.26404 | 0.001 | 2.6081 | 121 |
| ConA | Lent | 48 | MO000103567 | E2-C(m) | 0.40012 | 0.02 | 1.7788 | 121 |
| ConA | Lent | 48 | MO000044588 | parkin:Cul-1:Fbw7 | 0.23378 | 0.001 | 2.8546 | 123 |
| ConA | Lent | 48 | MO000082402 | MEKK1-xbb2(m) | 0.32206 | 0.002 | 2.2863 | 123 |
| ConA | Lent | 48 | MO000020892 | Par3(m) | 0.2471 | 0 | 2.7275 | 125 |
| ConA | Lent | 48 | MO000075867 | MEKK1(m) | 0.32206 | 0.002 | 2.2722 | 125 |
| ConA | Lent | 48 | MO000081629 | E1(m) | 0.51784 | 0.009 | 1.5409 | 127 |
| ConA | Lent | 48 | MO000088390 | IRF-8(m) | 0.21742 | 0.014 | 3.2253 | 127 |
| ConA | Lent | 48 | MO000032972 | IRF-3(m) | 0.22374 | 0.014 | 3.0055 | 129 |
| ConA | Lent | 48 | MO000119721 | tab3(m) | 0.27849 | 0 | 2.4523 | 130 |
| ConA | Lent | 48 | MO000041135 | tab2(m) | 0.27844 | 0 | 2.4482 | 132 |
| ConA | Lent | 48 | MO000021312 | PP2Cbeta1(m) | 0.39194 | 0.011 | 1.64 | 134 |
| ConA | Lent | 48 | MO000161841 | ube2h(m) | 0.30593 | 0.004 | 2.2512 | 135 |
| ConA | Lent | 48 | MO000009087 | MEKK1(m) | 0.46139 | 0.044 | 1.4243 | 138 |
| ConA | Lent | 48 | MO000083492 | RelA-p65(m) | 0.21613 | 0.016 | 3.0042 | 138 |
| ConA | Lent | 48 | MO000146524 | pkmyt1(m) | 0.40212 | 0.012 | 1.5426 | 138 |
| ConA | Lent | 48 | MO000031373 | Usp4(m) | 0.32565 | 0.015 | 1.9296 | 139 |
| ConA | Lent | 48 | MO000127914 | Cdc25C(m) | 0.40205 | 0.015 | 1.5253 | 141 |
| ConA | Lent | 48 | MO000007186 | IRF-8(m) | 0.21731 | 0.019 | 2.8734 | 144 |
| ConA | Lent | 48 | MO000042734 | TACE(m) | 0.23538 | 0.013 | 2.5549 | 145 |
| ConA | Lent | 48 | MO000169114 | 20S proteasome(m) | 0.2967 | 0.012 | 2.1942 | 145 |
| ConA | Lent | 48 | MO000095704 | TRIF(m) | 0.27428 | 0.001 | 2.3144 | 147 |
| ConA | Lent | 48 | MO000131412 | E1:Ubc5C{ub(1)} | 0.20499 | 0 | 2.9924 | 150 |
| ConA | Lent | 48 | MO000165399 | IKK-i(m){p} | 0.22633 | 0.018 | 2.6274 | 150 |
| ConA | Lent | 48 | MO000165055 | cIAP-1(m) | 0.28155 | 0.011 | 2.2432 | 151 |
| ConA | Lent | 48 | MO000187933 | Wee1(m) | 0.40156 | 0.036 | 1.3434 | 151 |
| ConA | Lent | 48 | MO000086968 | HPK1(m) | 0.28271 | 0.005 | 2.2154 | 152 |
| ConA | Lent | 48 | MO000152187 | AKT-1(m){p} | 0.31466 | 0.027 | 1.7672 | 153 |
| ConA | Lent | 48 | MO000019364 | RelA-p65(m) | 0.28511 | 0.01 | 2.148 | 154 |
| ConA | Lent | 48 | MO000233616 | Gprc5b(m) | 0.26389 | 0 | 2.3069 | 154 |
| ConA | Lent | 48 | MO000087611 | Cdk1(m) | 0.38963 | 0.049 | 1.3113 | 156 |
| ConA | Lent | 48 | MO000188322 | E1{ub(1)}:Ubc13:Mms2 | 0.22913 | 0.001 | 2.4927 | 159 |
| ConA | Lent | 48 | MO000120961 | E1:Ubc7{ub(1)} | 0.22913 | 0.001 | 2.4883 | 161 |
| ConA | Lent | 48 | MO000119264 | E1:UbcH7{ub(1)} | 0.22913 | 0.001 | 2.4728 | 164 |
| ConA | Lent | 48 | MO000160215 | E1:Ubc5A{ub(1)} | 0.22914 | 0.001 | 2.4438 | 165 |
| ConA | Lent | 48 | MO000120050 | UBE2G2(m) | 0.22893 | 0.001 | 2.4672 | 166 |
| ConA | Lent | 48 | MO000179234 | USP10(m) | 0.26376 | 0.015 | 2.036 | 171 |
| ConA | Lent | 48 | MO000187670 | E1{ub(1)}:Ubc13:Uev1 | 0.22913 | 0.001 | 2.4129 | 172 |
| ConA | Lent | 48 | MO000042840 | ptpn21(m) | 0.31413 | 0.041 | 1.5783 | 173 |
| ConA | Lent | 48 | MO000031928 | TNF-alpha(m) | 0.29689 | 0.035 | 1.6517 | 174 |
| ConA | Lent | 48 | MO000098006 | Nod2(m) | 0.21045 | 0.001 | 2.5716 | 174 |
| ConA | Lent | 48 | MO000032890 | PTP-PEST(m) | 0.31935 | 0.04 | 1.5086 | 175 |
| ConA | Lent | 48 | MO000120044 | UbcH7(m) | 0.25209 | 0.022 | 2.1484 | 176 |
| ConA | Lent | 48 | MO000038315 | LPS:lbp:CD14:TLR4:MD-2:TIRAP | 0.21219 | 0 | 2.5439 | 177 |
| ConA | Lent | 48 | MO000019483 | map4k4(m) | 0.2585 | 0.015 | 1.9818 | 179 |
| ConA | Lent | 48 | MO000022224 | dsRNA | 0.22272 | 0.005 | 2.4219 | 180 |
| ConA | Lent | 48 | MO000087375 | PIAS3-isoform2(m) | 0.29477 | 0.045 | 1.6109 | 181 |
| ConA | Lent | 48 | MO000019403 | SITPEC(m) | 0.25301 | 0.026 | 1.9052 | 185 |
| ConA | Lent | 48 | MO000062597 | FCRG(m) | 0.24504 | 0.018 | 2.0147 | 185 |
| ConA | Lent | 48 | MO000120702 | p62(m) | 0.28489 | 0.024 | 1.6362 | 185 |
| ConA | Lent | 48 | MO000085229 | cyclinB1(m) | 0.30116 | 0.038 | 1.4819 | 186 |
| ConA | Lent | 48 | MO000030839 | Wee1(m) | 0.26443 | 0.021 | 1.6606 | 189 |
| ConA | Lent | 48 | MO000037747 | LynA(m) | 0.30882 | 0.037 | 1.2844 | 190 |
| ConA | Lent | 48 | MO000023295 | Cdk6(m) | 0.24762 | 0.043 | 1.8142 | 191 |
| ConA | Lent | 48 | MO000037746 | LynB(m) | 0.30882 | 0.037 | 1.2841 | 192 |
| ConA | Lent | 48 | MO000020525 | Bax(m) | 0.2298 | 0.023 | 1.8749 | 200 |
| ConA | Lent | 48 | MO000057591 | PLCgamma2(m) | 0.22558 | 0.01 | 2.0483 | 202 |
| ConA | Lent | 48 | MO000119664 | IRAK-4(m) | 0.20935 | 0.017 | 2.3291 | 202 |
| ConA | Lent | 48 | MO000039080 | IRAK-4(m) | 0.20947 | 0.015 | 2.3029 | 206 |
| ConA | Lent | 48 | MO000156307 | FcepsilonRI beta(m) | 0.26414 | 0.041 | 1.4479 | 206 |
| ConA | Lent | 48 | MO000018208 | FcgammaRI | 0.22614 | 0.023 | 1.8213 | 209 |
| ConA | Lent | 48 | MO000046028 | NF-YA-isoform1(m) | 0.26477 | 0.033 | 1.3505 | 209 |
| ConA | Lent | 48 | MO000236690 | Mad2l1bp(m) | 0.21403 | 0.01 | 2.1914 | 210 |
| ConA | Lent | 48 | MO000095010 | pawr(m) | 0.24865 | 0.05 | 1.6097 | 211 |
| ConA | Lent | 48 | MO000017821 | Igalpha:Igbeta | 0.22604 | 0.023 | 1.7998 | 212 |
| ConA | Lent | 48 | MO000017705 | FcepsilonRI | 0.23138 | 0.036 | 1.6868 | 214 |
| ConA | Lent | 48 | MO000019413 | cd40(m) | 0.21764 | 0.032 | 1.79 | 221 |
| ConA | Lent | 48 | MO000086689 | NRG2(m) | 0.23425 | 0.04 | 1.5604 | 222 |
| ConA | Lent | 48 | MO000166537 | FcgammaRIII(m) | 0.22555 | 0.032 | 1.6377 | 230 |
| ConA | Lent | 48 | MO000103566 | E2-C(m) | 0.21442 | 0.039 | 1.7143 | 234 |
| ConA | Lent | 48 | MO000020140 | TCR(m) | 0.21929 | 0.031 | 1.6187 | 235 |
| ConA | Lent | 48 | MO000032784 | PLCgamma1(m) | 0.20096 | 0.029 | 1.7799 | 236 |
| ConA | Lent | 48 | MO000020142 | BCR(m) | 0.20666 | 0.021 | 1.7089 | 243 |
| ConA | Lent | 48 | MO000043647 | Tome-1(m) | 0.21366 | 0.021 | 1.587 | 247 |
| ConA | Lent | 48 | MO000337385 | Cdk1(m){p} | 0.21846 | 0.029 | 1.4329 | 248 |
| ConA | Lent | 48 | MO000199484 | Cdk1(m){pY15} | 0.21846 | 0.029 | 1.3522 | 252 |
| ConA | Lent | 48 | MO000087723 | Cdk1(m):cyclinB1(m) | 0.21313 | 0.048 | 1.4259 | 258 |
| ConA | Lent | 96 | MO000089823 | cIAP-2(m) | 0.39711 | 0.042 | 3.0786 | 25 |
| ConA | Lent | 96 | MO000019336 | TBK1(m) | 0.46858 | 0.001 | 2.5474 | 27 |
| ConA | Lent | 96 | MO000232618 | beta-TrCP1-isoform1(m) | 0.34587 | 0.031 | 3.2747 | 41 |
| ConA | Lent | 96 | MO000200602 | march5(m) | 0.37049 | 0.003 | 2.5558 | 44 |
| ConA | Lent | 96 | MO000041182 | TRIF(m) | 0.36264 | 0.004 | 2.6005 | 51 |
| ConA | Lent | 96 | MO000234738 | Irak2(m) | 0.32163 | 0.006 | 2.7292 | 52 |
| ConA | Lent | 96 | MO000089319 | Caspase(m) | 0.44184 | 0.038 | 2.2326 | 54 |
| ConA | Lent | 96 | MO000098007 | Nod2(m) | 0.36475 | 0.005 | 2.4476 | 55 |
| ConA | Lent | 96 | MO000024810 | ER-alpha(m) | 0.27602 | 0.038 | 3.158 | 59 |
| ConA | Lent | 96 | MO000020508 | HRS(m) | 0.37057 | 0.005 | 2.2989 | 61 |
| ConA | Lent | 96 | MO000059659 | IRAK-1(m){p} | 0.3628 | 0.007 | 2.4131 | 61 |
| ConA | Lent | 96 | MO000086151 | traf6-isoform1(m) | 0.37761 | 0.019 | 2.2759 | 61 |
| ConA | Lent | 96 | MO000120045 | UbcH7(m) | 0.44169 | 0.019 | 2.1834 | 61 |
| ConA | Lent | 96 | MO000200601 | march5(m) | 0.37048 | 0.006 | 2.2997 | 62 |
| ConA | Lent | 96 | MO000245279 | Usp25(m) | 0.36631 | 0.007 | 2.3446 | 62 |
| ConA | Lent | 96 | MO000032775 | p75NTR(m) | 0.31675 | 0.009 | 2.5938 | 63 |
| ConA | Lent | 96 | MO000121034 | CIKS(m) | 0.36275 | 0.007 | 2.3971 | 63 |
| ConA | Lent | 96 | MO000020145 | TRAF2(m) | 0.34437 | 0.012 | 2.4274 | 64 |
| ConA | Lent | 96 | MO000013127 | STAT5A(m) | 0.26873 | 0.023 | 2.8218 | 66 |
| ConA | Lent | 96 | MO000017453 | TAK1(m) | 0.48105 | 0.007 | 1.9754 | 68 |
| ConA | Lent | 96 | MO000038316 | LPS:lbp:CD14:TLR4:MD-2:TIRAP:IRAK-2 | 0.39992 | 0.005 | 2.1744 | 69 |
| ConA | Lent | 96 | MO000041437 | dsRNA:TLR3:TRIF | 0.39992 | 0.005 | 2.1748 | 69 |
| ConA | Lent | 96 | MO000117511 | TC-PTPa(m) | 0.49204 | 0.016 | 1.9281 | 69 |
| ConA | Lent | 96 | MO000128620 | EAC(m) | 0.425 | 0.01 | 2.0763 | 69 |
| ConA | Lent | 96 | MO000021719 | A20(m) | 0.38124 | 0.01 | 2.1858 | 70 |
| ConA | Lent | 96 | MO000022056 | Jak1(m) | 0.51848 | 0.008 | 1.8922 | 70 |
| ConA | Lent | 96 | MO000120015 | TLR3(m) | 0.2597 | 0 | 3.2806 | 70 |
| ConA | Lent | 96 | MO000022202 | IRAK-1(m) | 0.36676 | 0.026 | 2.2547 | 71 |
| ConA | Lent | 96 | MO000013134 | STAT5B(m) | 0.26646 | 0.028 | 2.69 | 72 |
| ConA | Lent | 96 | MO000113198 | T6BP(m) | 0.37017 | 0.008 | 2.2289 | 73 |
| ConA | Lent | 96 | MO000019402 | traf6(m) | 0.56652 | 0.032 | 1.8262 | 74 |
| ConA | Lent | 96 | MO000022316 | PKCiota(m) | 0.39312 | 0.011 | 2.1303 | 74 |
| ConA | Lent | 96 | MO000025777 | STAT3(m) | 0.31818 | 0.03 | 2.3594 | 74 |
| ConA | Lent | 96 | MO000007348 | IL-2Rbeta(m) | 0.41149 | 0.012 | 2.0043 | 75 |
| ConA | Lent | 96 | MO000086968 | HPK1(m) | 0.31771 | 0.008 | 2.3287 | 78 |
| ConA | Lent | 96 | MO000119981 | NIK(m) | 0.4812 | 0.011 | 1.8529 | 79 |
| ConA | Lent | 96 | MO000149650 | TBK1(m) | 0.26356 | 0.003 | 2.6217 | 80 |
| ConA | Lent | 96 | MO000032271 | SOCS-1(m) | 0.41365 | 0.012 | 1.9209 | 81 |
| ConA | Lent | 96 | MO000059714 | ErbB3(m) | 0.36108 | 0.03 | 2.2289 | 82 |
| ConA | Lent | 96 | MO000008302 | Syk(m) | 0.49267 | 0.028 | 1.7456 | 84 |
| ConA | Lent | 96 | MO000036067 | TRAF3(m) | 0.27717 | 0.006 | 2.3548 | 84 |
| ConA | Lent | 96 | MO000044588 | parkin:Cul-1:Fbw7 | 0.24253 | 0.003 | 2.8773 | 84 |
| ConA | Lent | 96 | MO000119664 | IRAK-4(m) | 0.25123 | 0.005 | 2.7105 | 84 |
| ConA | Lent | 96 | MO000120044 | UbcH7(m) | 0.29519 | 0.027 | 2.32 | 84 |
| ConA | Lent | 96 | MO000121772 | A20(m) | 0.39058 | 0.015 | 1.9497 | 85 |
| ConA | Lent | 96 | MO000079038 | SOCS-1(m) | 0.38107 | 0.029 | 1.9581 | 86 |
| ConA | Lent | 96 | MO000245273 | Usp22(m) | 0.48349 | 0.034 | 1.7499 | 86 |
| ConA | Lent | 96 | MO000016606 | IKK-gamma(m) | 0.34152 | 0.024 | 2.185 | 88 |
| ConA | Lent | 96 | MO000081385 | TAK1(m) | 0.29528 | 0.01 | 2.2719 | 88 |
| ConA | Lent | 96 | MO000336179 | TAK1(m){ub} | 0.31255 | 0.01 | 2.2626 | 89 |
| ConA | Lent | 96 | MO000039080 | IRAK-4(m) | 0.24952 | 0.006 | 2.5142 | 93 |
| ConA | Lent | 96 | MO000079029 | Jak1(m) | 0.31301 | 0.016 | 2.1955 | 94 |
| ConA | Lent | 96 | MO000022549 | IKK-beta(m) | 0.48721 | 0.016 | 1.693 | 95 |
| ConA | Lent | 96 | MO000088836 | NF-kappaB1(m) | 0.28742 | 0.042 | 2.2341 | 95 |
| ConA | Lent | 96 | MO000038315 | LPS:lbp:CD14:TLR4:MD-2:TIRAP | 0.22167 | 0.004 | 2.7408 | 99 |
| ConA | Lent | 96 | MO000019250 | RIP(m) | 0.24546 | 0.009 | 2.4302 | 100 |
| ConA | Lent | 96 | MO000032972 | IRF-3(m) | 0.2054 | 0.034 | 3.4171 | 100 |
| ConA | Lent | 96 | MO000041135 | tab2(m) | 0.26605 | 0.004 | 2.2722 | 100 |
| ConA | Lent | 96 | MO000119721 | tab3(m) | 0.2661 | 0.004 | 2.2709 | 101 |
| ConA | Lent | 96 | MO000022062 | Jak3(m) | 0.59539 | 0.032 | 1.5166 | 102 |
| ConA | Lent | 96 | MO000013131 | STAT6(m) | 0.2177 | 0.045 | 2.7282 | 104 |
| ConA | Lent | 96 | MO000165629 | IKK-beta(m){pS177}{pS181} | 0.23653 | 0.004 | 2.4562 | 104 |
| ConA | Lent | 96 | MO000045239 | Aos1(m):SAE2(m) | 0.54926 | 0.028 | 1.3604 | 105 |
| ConA | Lent | 96 | MO000019485 | IFNalpha, IFNbeta:IFNAR1{pY}:Tyk2{pY}:IFNAR2c{pY}:Jak1{pY} | 0.28924 | 0.02 | 2.1078 | 106 |
| ConA | Lent | 96 | MO000098006 | Nod2(m) | 0.22575 | 0.006 | 2.4675 | 109 |
| ConA | Lent | 96 | MO000170572 | IKK-beta(m){pS} | 0.22768 | 0.006 | 2.4324 | 109 |
| ConA | Lent | 96 | MO000017162 | c-Kit(m) | 0.4314 | 0.05 | 1.5918 | 111 |
| ConA | Lent | 96 | MO000032104 | c-Kit(m){pY} | 0.23153 | 0.007 | 2.4246 | 111 |
| ConA | Lent | 96 | MO000033240 | EpoR(m) | 0.40731 | 0.027 | 1.6525 | 111 |
| ConA | Lent | 96 | MO000088390 | IRF-8(m) | 0.20815 | 0.044 | 2.6293 | 111 |
| ConA | Lent | 96 | MO000006539 | Hck(m) | 0.45194 | 0.045 | 1.5207 | 112 |
| ConA | Lent | 96 | MO000032858 | TRAF4(m) | 0.31953 | 0.03 | 1.8291 | 117 |
| ConA | Lent | 96 | MO000082402 | MEKK1-xbb2(m) | 0.33194 | 0.033 | 1.7655 | 119 |
| ConA | Lent | 96 | MO000002525 | CD28(m) | 0.25844 | 0.022 | 2.0637 | 125 |
| ConA | Lent | 96 | MO000075867 | MEKK1(m) | 0.33194 | 0.035 | 1.743 | 125 |
| ConA | Lent | 96 | MO000007259 | IGF-2(m) | 0.26613 | 0.028 | 1.9019 | 128 |
| ConA | Lent | 96 | MO000032013 | Jak2(m){p} | 0.26955 | 0.047 | 1.8663 | 129 |
| ConA | Lent | 96 | MO000059191 | AKT(m){p} | 0.36603 | 0.037 | 1.5932 | 129 |
| ConA | Lent | 96 | MO000016669 | IFNRI | 0.27455 | 0.027 | 1.8129 | 131 |
| ConA | Lent | 96 | MO000036021 | EDAR(m) | 0.23663 | 0.016 | 2.0629 | 139 |
| ConA | Lent | 96 | MO000017252 | IL-10R | 0.26401 | 0.028 | 1.7934 | 140 |
| ConA | Lent | 96 | MO000089140 | gp130(m) | 0.25168 | 0.018 | 1.8778 | 141 |
| ConA | Lent | 96 | MO000019522 | IL-22R1:IL-10R2 | 0.26401 | 0.028 | 1.7934 | 142 |
| ConA | Lent | 96 | MO000022224 | dsRNA | 0.23743 | 0.016 | 1.9617 | 142 |
| ConA | Lent | 96 | MO000019364 | RelA-p65(m) | 0.23622 | 0.041 | 1.9289 | 148 |
| ConA | Lent | 96 | MO000020892 | Par3(m) | 0.22061 | 0.013 | 2.0448 | 151 |
| ConA | Lent | 96 | MO000166537 | FcgammaRIII(m) | 0.25631 | 0.034 | 1.7453 | 152 |
| ConA | Lent | 96 | MO000127428 | Rock-1(m) | 0.27311 | 0.049 | 1.6007 | 153 |
| ConA | Lent | 96 | MO000130073 | PKCiota(m) | 0.20337 | 0.008 | 2.1355 | 153 |
| ConA | Lent | 96 | MO000062597 | FCRG(m) | 0.25527 | 0.044 | 1.7363 | 157 |
| ConA | Lent | 96 | MO000017705 | FcepsilonRI | 0.26182 | 0.044 | 1.6775 | 159 |
| ConA | Lent | 96 | MO000017821 | Igalpha:Igbeta | 0.24576 | 0.036 | 1.7272 | 162 |
| ConA | Lent | 96 | MO000032836 | IFNAR2(m) | 0.23874 | 0.03 | 1.7402 | 164 |
| ConA | Lent | 96 | MO000032852 | OSMRbeta(m) | 0.23872 | 0.03 | 1.7406 | 164 |
| ConA | Lent | 96 | MO000018208 | FcgammaRI | 0.2438 | 0.039 | 1.6657 | 170 |
| ConA | Lent | 96 | MO000032843 | CrkL(m) | 0.22121 | 0.043 | 1.7534 | 171 |
| ConA | Lent | 96 | MO000285932 | Cezanne(m) | 0.20283 | 0.032 | 1.8719 | 172 |
| ConA | Lent | 96 | MO000057591 | PLCgamma2(m) | 0.22707 | 0.05 | 1.7096 | 175 |
| ConA | Lent | 96 | MO000036576 | BCAR-3(m) | 0.22285 | 0.04 | 1.6852 | 181 |
| ConA | Lent | 96 | MO000032784 | PLCgamma1(m) | 0.22675 | 0.046 | 1.6468 | 183 |
| ConA | Lent | 96 | MO000056866 | EpoR-F(m) | 0.20441 | 0.049 | 1.7065 | 189 |
| ConA | Lent | 96 | MO000032774 | RIP1(m) | 0.21679 | 0.049 | 1.581 | 196 |
| ConA | Lent | 96 | MO000130207 | Arhgef9(m) | 0.21678 | 0.049 | 1.5813 | 196 |
| ConA | Lent | 96 | MO000199377 | Fgd1(m) | 0.21679 | 0.049 | 1.5808 | 196 |
| ConA + Poly I:C | Lent | 48 | MO000032382 | KSR(m) | 0.43176 | 0.019 | 1.4587 | 21 |
| ConA + Poly I:C | Lent | 48 | MO000280542 | (angiotensin II)2:(AT2 receptor)2 | 0.40388 | 0.034 | 1.5071 | 24 |
| ConA + Poly I:C | Lent | 48 | MO000033710 | ACLP-isoform1(m) | 0.28571 | 0.027 | 1.6712 | 26 |
| ConA + Poly I:C | Lent | 48 | MO000056021 | C/EBPbeta(m) | 0.27053 | 0.027 | 2.4667 | 26 |
| ConA + Poly I:C | Lent | 48 | MO000084411 | RSK2(m) | 0.44476 | 0.034 | 1.3964 | 26 |
| ConA + Poly I:C | Lent | 48 | MO000131365 | ACLP(m) | 0.47698 | 0.048 | 1.3629 | 27 |
| ConA + Poly I:C | Lent | 48 | MO000196229 | ERK1(m){isg} | 0.32333 | 0.018 | 1.4968 | 29 |
| ConA + Poly I:C | Lent | 48 | MO000281378 | (angiotensin II)2:(AT2 receptor)2:(ATIP-isoform3)2 | 0.3388 | 0.023 | 1.4628 | 30 |
| ConA + Poly I:C | Lent | 48 | MO000056800 | RXR-alpha(m) | 0.2411 | 0.048 | 2.6277 | 32 |
| ConA + Poly I:C | Lent | 48 | MO000059832 | PTP-SL(m) | 0.51379 | 0.023 | 1.3048 | 33 |
| ConA + Poly I:C | Lent | 48 | MO000239976 | Ptpn7(m) | 0.54642 | 0.017 | 1.2698 | 33 |
| ConA + Poly I:C | Lent | 48 | MO000026350 | C/EBPbeta-LIP(m) | 0.23255 | 0.014 | 2.8153 | 35 |
| ConA + Poly I:C | Lent | 48 | MO000077897 | ERK1(m){pY} | 0.32333 | 0.035 | 1.4252 | 36 |
| ConA + Poly I:C | Lent | 48 | MO000281382 | (ATIP-isoform3)2:SHP-1 | 0.40392 | 0.034 | 1.3907 | 36 |
| ConA + Poly I:C | Lent | 48 | MO000038590 | Rac1:GTP:MEKK4 | 0.41982 | 0.045 | 1.341 | 37 |
| ConA + Poly I:C | Lent | 48 | MO000121303 | Raf(m){p} | 0.43105 | 0.028 | 1.3379 | 37 |
| ConA + Poly I:C | Lent | 48 | MO000007960 | Cot(m) | 0.56246 | 0.048 | 1.2127 | 39 |
| ConA + Poly I:C | Lent | 48 | MO000032857 | MEKK4(m) | 0.43186 | 0.031 | 1.2811 | 39 |
| ConA + Poly I:C | Lent | 48 | MO000085073 | Mapk(m){p} | 0.26425 | 0.045 | 1.443 | 39 |
| ConA + Poly I:C | Lent | 48 | MO000239971 | Ptpn5(m) | 0.54513 | 0.026 | 1.2291 | 39 |
| ConA + Poly I:C | Lent | 48 | MO000243058 | Stk25(m) | 0.28028 | 0.044 | 1.4072 | 41 |
| ConA + Poly I:C | Lent | 48 | MO000038749 | (TRAF2)2:(ASK1{p})2 | 0.41982 | 0.045 | 1.3166 | 42 |
| ConA + Poly I:C | Lent | 48 | MO000043173 | Rac1:GTP:(MLK3{p})2 | 0.37914 | 0.043 | 1.3385 | 42 |
| ConA + Poly I:C | Lent | 48 | MO000021709 | TNFR1(m) | 0.22487 | 0.024 | 1.6374 | 43 |
| ConA + Poly I:C | Lent | 48 | MO000024714 | c-Ets-1(m) | 0.22337 | 0.039 | 1.8098 | 43 |
| ConA + Poly I:C | Lent | 48 | MO000120857 | CD154(m) | 0.24191 | 0.043 | 1.4337 | 43 |
| ConA + Poly I:C | Lent | 48 | MO000038806 | PKCdelta:DAG:Raf-1 | 0.43106 | 0.028 | 1.2479 | 45 |
| ConA + Poly I:C | Lent | 48 | MO000128521 | MEK(m){p} | 0.51278 | 0.05 | 1.1735 | 45 |
| ConA + Poly I:C | Lent | 48 | MO000281381 | (angiotensin II)2:(AT2 receptor)2:(ATIP-isoform3)2:SHP-1 | 0.53182 | 0.047 | 1.1447 | 45 |
| ConA + Poly I:C | Lent | 48 | MO000281371 | AT2 receptor:ATIP-isoform3 | 0.22591 | 0.012 | 1.4967 | 46 |
| ConA + Poly I:C | Lent | 48 | MO000084682 | MEKK4-A(m) | 0.42094 | 0.049 | 1.252 | 47 |
| ConA + Poly I:C | Lent | 48 | MO000034282 | CD91(m) | 0.21269 | 0.03 | 1.5561 | 49 |
| ConA + Poly I:C | Lent | 48 | MO000120192 | SIGIRR(m) | 0.24191 | 0.043 | 1.3494 | 49 |
| ConA + Poly I:C | Lent | 48 | MO000038623 | Rac1:GTP:POSH:MLK3:MKK4, MKK7:JNK1, JNK2 | 0.2273 | 0.046 | 1.418 | 53 |
| ConA + Poly I:C | Lent | 48 | MO000079663 | CKIP1(m) | 0.21718 | 0.033 | 1.4504 | 54 |
| ConA + Poly I:C | Lent | 48 | MO000016807 | Ras:GTP:Raf{p} | 0.43106 | 0.028 | 1.0763 | 55 |
| ConA + Poly I:C | Lent | 48 | MO000043351 | RANKL(m):RANK(m) | 0.21036 | 0.042 | 1.4703 | 55 |
| ConA + Poly I:C | Lent | 48 | MO000178435 | DAG:PKCdelta(m) | 0.24112 | 0.03 | 1.3189 | 55 |
| ConA + Poly I:C | Lent | 48 | MO000057223 | Ras(m):GTP | 0.24112 | 0.03 | 1.282 | 60 |
| ConA + Poly I:C | Lent | 48 | MO000198693 | periostin(m) | 0.23356 | 0.039 | 1.3058 | 62 |
| ConA + Poly I:C | Lent | 48 | MO000033460 | JIP1-isoform1(m) | 0.25658 | 0.048 | 1.2225 | 64 |
| ConA + Poly I:C | Lent | 48 | MO000280350 | Ptpn7(m){pS44} | 0.23387 | 0.024 | 1.1696 | 74 |
| ConA + Poly I:C | Lent | 48 | MO000100787 | CaSR(m) | 0.21677 | 0.017 | 1.2585 | 75 |
| ConA + Poly I:C | Lent | 48 | MO000158713 | KSR-isoform1(m) | 0.22474 | 0.036 | 1.2374 | 75 |
| ConA + Poly I:C | Lent | 48 | MO000102593 | Itk(m) | 0.23436 | 0.045 | 1.0645 | 77 |
| ConA + Poly I:C | Lent | 48 | MO000018020 | MOR(m) | 0.21175 | 0.049 | 1.0916 | 87 |
| ConA + Poly I:C | Lent | 96 | MO000022450 | PKCzeta(m) | 0.49933 | 0.013 | 2.0665 | 19 |
| ConA + Poly I:C | Lent | 96 | MO000019336 | TBK1(m) | 0.40617 | 0.008 | 2.3534 | 20 |
| ConA + Poly I:C | Lent | 96 | MO000041182 | TRIF(m) | 0.34584 | 0.002 | 2.8238 | 22 |
| ConA + Poly I:C | Lent | 96 | MO000127428 | Rock-1(m) | 0.29907 | 0.003 | 2.9102 | 28 |
| ConA + Poly I:C | Lent | 96 | MO000336179 | TAK1(m){ub} | 0.28342 | 0.007 | 2.3919 | 40 |
| ConA + Poly I:C | Lent | 96 | MO000095704 | TRIF(m) | 0.25524 | 0.001 | 2.9076 | 41 |
| ConA + Poly I:C | Lent | 96 | MO000059191 | AKT(m){p} | 0.35509 | 0.008 | 1.9572 | 43 |
| ConA + Poly I:C | Lent | 96 | MO000022549 | IKK-beta(m) | 0.47028 | 0.011 | 1.7614 | 44 |
| ConA + Poly I:C | Lent | 96 | MO000036067 | TRAF3(m) | 0.25918 | 0.006 | 2.67 | 45 |
| ConA + Poly I:C | Lent | 96 | MO000120702 | p62(m) | 0.3112 | 0.018 | 2.0236 | 45 |
| ConA + Poly I:C | Lent | 96 | MO000200602 | march5(m) | 0.31194 | 0.015 | 2.0051 | 46 |
| ConA + Poly I:C | Lent | 96 | MO000022316 | PKCiota(m) | 0.34544 | 0.009 | 1.9455 | 47 |
| ConA + Poly I:C | Lent | 96 | MO000149650 | TBK1(m) | 0.24918 | 0.002 | 2.7983 | 47 |
| ConA + Poly I:C | Lent | 96 | MO000020145 | TRAF2(m) | 0.29031 | 0.023 | 2.0973 | 48 |
| ConA + Poly I:C | Lent | 96 | MO000078606 | PIAS1(m) | 0.42916 | 0.033 | 1.7518 | 48 |
| ConA + Poly I:C | Lent | 96 | MO000081385 | TAK1(m) | 0.25992 | 0.006 | 2.2721 | 49 |
| ConA + Poly I:C | Lent | 96 | MO000119981 | NIK(m) | 0.43802 | 0.024 | 1.7083 | 49 |
| ConA + Poly I:C | Lent | 96 | MO000016606 | IKK-gamma(m) | 0.29112 | 0.037 | 2.0171 | 52 |
| ConA + Poly I:C | Lent | 96 | MO000032934 | ILK(m) | 0.4657 | 0.037 | 1.6671 | 52 |
| ConA + Poly I:C | Lent | 96 | MO000020525 | Bax(m) | 0.24181 | 0.008 | 2.3838 | 53 |
| ConA + Poly I:C | Lent | 96 | MO000128620 | EAC(m) | 0.37447 | 0.027 | 1.7771 | 54 |
| ConA + Poly I:C | Lent | 96 | MO000232618 | beta-TrCP1-isoform1(m) | 0.25592 | 0.04 | 2.1836 | 54 |
| ConA + Poly I:C | Lent | 96 | MO000032858 | TRAF4(m) | 0.28686 | 0.019 | 2.0112 | 55 |
| ConA + Poly I:C | Lent | 96 | MO000151911 | IKK-alpha(m){p} | 0.24999 | 0.017 | 2.1873 | 55 |
| ConA + Poly I:C | Lent | 96 | MO000019250 | RIP(m) | 0.22087 | 0.005 | 2.8269 | 57 |
| ConA + Poly I:C | Lent | 96 | MO000035076 | CnAalpha(m) | 0.40262 | 0.017 | 1.6887 | 57 |
| ConA + Poly I:C | Lent | 96 | MO000098007 | Nod2(m) | 0.3034 | 0.03 | 1.8746 | 58 |
| ConA + Poly I:C | Lent | 96 | MO000146524 | pkmyt1(m) | 0.42327 | 0.038 | 1.6347 | 59 |
| ConA + Poly I:C | Lent | 96 | MO000179244 | SKB1(m) | 0.28186 | 0.03 | 1.9777 | 59 |
| ConA + Poly I:C | Lent | 96 | MO000200601 | march5(m) | 0.31193 | 0.035 | 1.8153 | 59 |
| ConA + Poly I:C | Lent | 96 | MO000120015 | TLR3(m) | 0.21495 | 0.004 | 2.8889 | 60 |
| ConA + Poly I:C | Lent | 96 | MO000036021 | EDAR(m) | 0.22371 | 0.007 | 2.3776 | 62 |
| ConA + Poly I:C | Lent | 96 | MO000045239 | Aos1(m):SAE2(m) | 0.49563 | 0.049 | 1.3558 | 62 |
| ConA + Poly I:C | Lent | 96 | MO000127914 | Cdc25C(m) | 0.4232 | 0.041 | 1.5906 | 63 |
| ConA + Poly I:C | Lent | 96 | MO000337385 | Cdk1(m){p} | 0.2337 | 0.014 | 2.1793 | 63 |
| ConA + Poly I:C | Lent | 96 | MO000059659 | IRAK-1(m){p} | 0.30098 | 0.033 | 1.8124 | 64 |
| ConA + Poly I:C | Lent | 96 | MO000120045 | UbcH7(m) | 0.39253 | 0.047 | 1.6226 | 64 |
| ConA + Poly I:C | Lent | 96 | MO000245279 | Usp25(m) | 0.3056 | 0.036 | 1.7899 | 64 |
| ConA + Poly I:C | Lent | 96 | MO000017453 | TAK1(m) | 0.42365 | 0.044 | 1.4835 | 65 |
| ConA + Poly I:C | Lent | 96 | MO000199484 | Cdk1(m){pY15} | 0.2337 | 0.014 | 2.1793 | 65 |
| ConA + Poly I:C | Lent | 96 | MO000020892 | Par3(m) | 0.21081 | 0 | 2.7154 | 66 |
| ConA + Poly I:C | Lent | 96 | MO000044385 | parkin(m) | 0.27832 | 0.037 | 1.9101 | 66 |
| ConA + Poly I:C | Lent | 96 | MO000058796 | Bax-alpha(m) | 0.22137 | 0.011 | 2.2498 | 66 |
| ConA + Poly I:C | Lent | 96 | MO000121034 | CIKS(m) | 0.30095 | 0.035 | 1.7952 | 66 |
| ConA + Poly I:C | Lent | 96 | MO000095010 | pawr(m) | 0.26618 | 0.049 | 1.8606 | 69 |
| ConA + Poly I:C | Lent | 96 | MO000021719 | A20(m) | 0.31503 | 0.045 | 1.6411 | 70 |
| ConA + Poly I:C | Lent | 96 | MO000057073 | GSK3beta(m) | 0.37239 | 0.026 | 1.5212 | 71 |
| ConA + Poly I:C | Lent | 96 | MO000088836 | NF-kappaB1(m) | 0.22383 | 0.044 | 2.033 | 72 |
| ConA + Poly I:C | Lent | 96 | MO000057087 | AKT-1(m){pS473} | 0.37125 | 0.02 | 1.4896 | 73 |
| ConA + Poly I:C | Lent | 96 | MO000035203 | Rad23A(m) | 0.37882 | 0.036 | 1.2993 | 74 |
| ConA + Poly I:C | Lent | 96 | MO000117817 | PKA-RI-alpha(m) | 0.24001 | 0.028 | 1.9205 | 75 |
| ConA + Poly I:C | Lent | 96 | MO000002525 | CD28(m) | 0.21766 | 0.018 | 2.0383 | 76 |
| ConA + Poly I:C | Lent | 96 | MO000020927 | FAP-1(m) | 0.35384 | 0.042 | 1.4018 | 77 |
| ConA + Poly I:C | Lent | 96 | MO000106371 | CaMKII(m) | 0.22815 | 0.042 | 1.8518 | 84 |
| ConA + Poly I:C | Lent | 96 | MO000119721 | tab3(m) | 0.21767 | 0.014 | 1.9251 | 84 |
| ConA + Poly I:C | Lent | 96 | MO000041135 | tab2(m) | 0.21764 | 0.014 | 1.9192 | 88 |
| ConA + Poly I:C | Lent | 96 | MO000030839 | Wee1(m) | 0.26139 | 0.048 | 1.5971 | 90 |
| ConA + Poly I:C | Lent | 96 | MO000016793 | CaMKII{p} | 0.23661 | 0.041 | 1.6898 | 92 |
| ConA + Poly I:C | Lent | 96 | MO000019483 | map4k4(m) | 0.24384 | 0.04 | 1.6349 | 94 |
| ConA + Poly I:C | Lent | 96 | MO000086970 | CnAalpha-isoform1(m) | 0.22433 | 0.034 | 1.686 | 98 |
| ConA + Poly I:C | Lent | 96 | MO000171736 | AKT-2(m){pS} | 0.20111 | 0.022 | 1.8415 | 98 |
| ConA + Poly I:C | Lent | 96 | MO000043647 | Tome-1(m) | 0.21263 | 0.03 | 1.7369 | 103 |
| ConA + Poly I:C | Lent | 96 | MO000154875 | p110gamma(m) | 0.20878 | 0.047 | 1.5692 | 117 |
